# Mapping the Surface Microbiome and Metabolome of Brown Seaweed *Fucus vesiculosus* by Amplicon Sequencing, Integrated Metabolomics and Imaging Techniques

**DOI:** 10.1038/s41598-018-37914-8

**Published:** 2019-01-31

**Authors:** Delphine Parrot, Martina Blümel, Caroline Utermann, Giuseppina Chianese, Stefan Krause, Alexander Kovalev, Stanislav N. Gorb, Deniz Tasdemir

**Affiliations:** 1 GEOMAR Centre for Marine Biotechnology, Research Unit Marine Natural Products Chemistry, GEOMAR Helmholtz Centre for Ocean Research Kiel, Am Kiel-Kanal 44, Kiel, 24106 Germany; 2 Research Unit Marine Geosystems, GEOMAR Helmholtz Centre for Ocean Research Kiel, Wischhofstrasse 1-3, Kiel, 24148 Germany; 3 Department of Functional Morphology and Biomechanics, Institute of Zoology, Kiel University, Am Botanischen Garten 9, Kiel, 24118 Germany; 4 Kiel University, Christian-Albrechts-Platz 4, Kiel, 24118 Germany

## Abstract

The brown alga *Fucus vesiculosus* is a keystone marine species, which is subject to heavy surface colonisation. This study was designed to analyse the surface epibiome of *F*. *vesiculosus* in conjunction with the composition and spatial distribution of its surface metabolome. The amplicon sequencing, SEM and CARD-FISH imaging studies showed Alphaproteobacteria to predominate the epibiotic bacteria. Fungi of the class Eurotiomycetes were visualised for the first time on an algal surface. An untargeted metabolomics approach using molecular networks, *in silico* prediction and manual dereplication showed the differential metabolome of the surface and the whole tissue extracts. In total, 50 compounds were putatively dereplicated by UPLC-MS/MS, 37 of which were previously reported from both seaweeds and microorganisms. Untargeted spatial metabolomics by DESI-Imaging MS identified the specific localisation and distribution of various primary and secondary metabolites on surface imprints and in algal cross sections. The UPLC-MS, DESI-IMS and NMR analyses failed to confirm the presence of any surface-associated metabolite, except for mannitol, which were previously reported from *F*. *vesiculosus*. This is the first study analysing the seaweed surface microbiome in conjunction with untargeted surface metabolomics and spatial metabolomics approaches.

## Introduction

Macroalgal surfaces represent a physical interface to the surrounding seawater and are prone to rapid colonisation resulting in fouling and overgrowth^1,2^. Particularly bacteria play major structural and functional roles as epibionts and are the primary colonisers^2^. Macroalgae host a dense bacterial epibiome forming surface biofilms^2–4^. Although the bacterial colonisers are recruited from the surrounding water and vary with season^4,5^, the composition of the macroalgal biofilms differs from that of the seawater^6,7^. The algae-associated bacterial communities are host-species-specific; they differ between algal species^4,7^ and show temporal variation^5^. The epibiosis has both beneficial and detrimental effects on the host. The epiphytic bacterial community modulates algal growth, morphogenesis, reproduction and chemical defense against other micro- and macrofoulers^1,2,8^. However, they may also enhance the secondary colonisation of other bacteria, or the larvae and spores of macrofoulers^9^. Uncontrolled fouling may also impede the access to light, gas exchange, photosynthetic activity and alter interactions of the alga with pathogens and consumers^2,10^. Macroalgae use both mechanical and chemical methods to control and to shape the composition of their surface biofilm. The latter involves secondary metabolites (SMs) that are produced in the outermost tissues of the seaweed and exuded onto the surface^3,10,11^. For example, the furanones produced in the central vesicle glandular cells under the thallus surface of the red alga *Delisea pulchra* inhibit bacterial N-acyl homoserine lactone (AHL)-mediated quorum sensing for influencing the bacterial community composition^12^. In contrast to the well-studied bacteria-algae surface interactions, there is only little evidence on fungi and their role on macroalgal surfaces^3^.

The surface epibiota of algal thalli is considered as a miniature ecosystem - a seaweed holobiont^3^, in which the surface represents a crucial site for chemical signalling. However, our understanding on surface-mediated signalling processes and the mediator molecules is still very limited due to methodological difficulties. Current surface chemistry studies mostly rely on classical “selective” surface solvent extraction methods^13–16^ while a recent method used solid phase C18 adsorption^17^. Both methods are laborious, associated with low yield, poor reproducibility and destruction risk of outer membranes of epidermal cells^18^ and fail providing information on specific localisation or distribution of the surface-associated metabolites and were mostly used for targeted isolation of few alga-derived compounds, rather than a detailed metabolome analysis. Recently developed chemical imaging techniques, such as Desorption Electrospray Ionisation - Imaging Mass Spectrometry (DESI-IMS), circumvent destructive and laborious extraction or sample preparation allowing direct spatial localisation of metabolites on the organism’s surface or cross-sections at a µm-scale resolution^19,20^. Since the pioneering work of Lane *et al*. (2009) on the red alga *Callophycus serratus*^21^, however, only two studies have employed DESI-IMS on seaweed surfaces^22,23^.

Liquid chromatography coupled with mass spectrometry (LC-MS) has become the backbone of modern metabolomics studies for identification of global chemical profile of biological organisms. However, classical LC-MS-based untargeted metabolomics efforts suffer from low annotation rates (<2%)^24^, high frequency of molecular formula match to numerous compounds in databases and failing to provide conclusive hints on chemical relatedness of the individual metabolites. The recently established open-access platform Global Natural Product Social Molecular Networking (GNPS)^25^ uses an automated database comparison of LC-MS/MS fragments to cluster and visualise related molecules as spectral networks, providing a quantum leap in annotation and dereplication. By combining the high speed and the sensitivity of the MS with spatial and temporal chemical information, imaging mass spectrometry (IMS) enables visualisation of the site of production/location and the spatial distribution of metabolites on biological surfaces or cross-sections. IMS provides unsurpassed chemical detail^20^, hence appears as “spatial metabolomics” to complement the classical and molecular networking (MN) based in-depth metabolomics studies.

The bladder wrack *Fucus vesiculosus* is a brown alga widely distributed along the coastlines of the North Atlantic Ocean, Baltic and North Seas where it serves as primary producer, carbon sink and nursery ground for invertebrates and fish^26^. With a biofilm density reaching up to 10^8^ bacterial cells/cm^2^ on its surface^27^, it is the most heavily fouled Baltic *Fucus* species^28^. The fouling stress is highest in summer months, particularly in July^16^. *F*. *vesiculosus* shapes its epibiotic community by oxidative bursts as non-specific defense^29^. Several, presumably alga-derived compounds, i.e. dimethylsulfopropionate (DMSP), the amino acids proline and alanine, and the carotenoid pigment fucoxanthin have been reported to be involved in the chemical control of the surface fouling^30,31^, but a detailed surface metabolome study has not been carried out yet. Earlier studies have shown the bacterial community on *F*. *vesiculosus* biofilm to be rich in Proteobacteria and Bacteroidetes^11,32,33^, but to date no putative microbial metabolite has ever been reported or visualized from its surface. The aim of this study was to map the fine scale composition and distribution of the surface metabolome of Baltic Sea *F*. *vesiculosus* and to perform a high spatial resolution analysis of surface microbial community composition. Towards this aim, we first investigated the surface microbiome using (i) 16S rRNA gene amplicon sequencing (MiSeq) for identifying the bacterial community composition, (ii) scanning electron microscopy (SEM) to identify the biofilm density, (iii) CARD-FISH to detect specific distributions of selected surface-associated microorganisms (Actinobacteria, Firmicutes, Alphaproteobacteria and fungi). The metabolome of the surface extracts, the whole algal extracts and the surface-free extracts (obtained from the remaining algal material after surface extractions) were analysed comparatively by HRMS/MS molecular networking (MN) based metabolomics combined with *in silico* prediction of fragmentation patterns (ISDB)^34^ and manual dereplication. Finally, the surface imprints and cross sections of the alga were analysed by DESI-IMS-based untargeted spatial metabolomics approach, localising many compounds on different parts of the alga. This is the first study simultaneously analysing the microbiome and metabolome of the miniature surface ecosystem of *F*. *vesiculosus*.

## Results

### Analysis of surface microbiome combined with microscopic imaging

**Figure 1:**
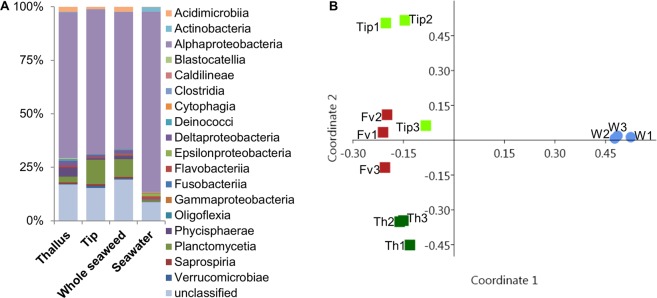
The surface microbiome of Baltic *F*. *vesiculosus* in comparison to the surrounding seawater reference. (**A**) Relative abundance of bacterial classes detected on the algal surface and in the ambient seawater. All reads corresponding to one bacterial class were summed up for each respective sample type (n = 3 for all sample types). (**B**) Bray-Curtis similarity index based PCoA plot (dark green: surface thallus sample of *F*. *vesiculosus* (Th1–3), light green: surface tip sample of *F*. *vesiculosus* (Tip1–3), red: whole seaweed surface sample of *F*. *vesiculosus* (Fv1–3), blue: seawater reference sample (W1–3)).

**Figure 2:**
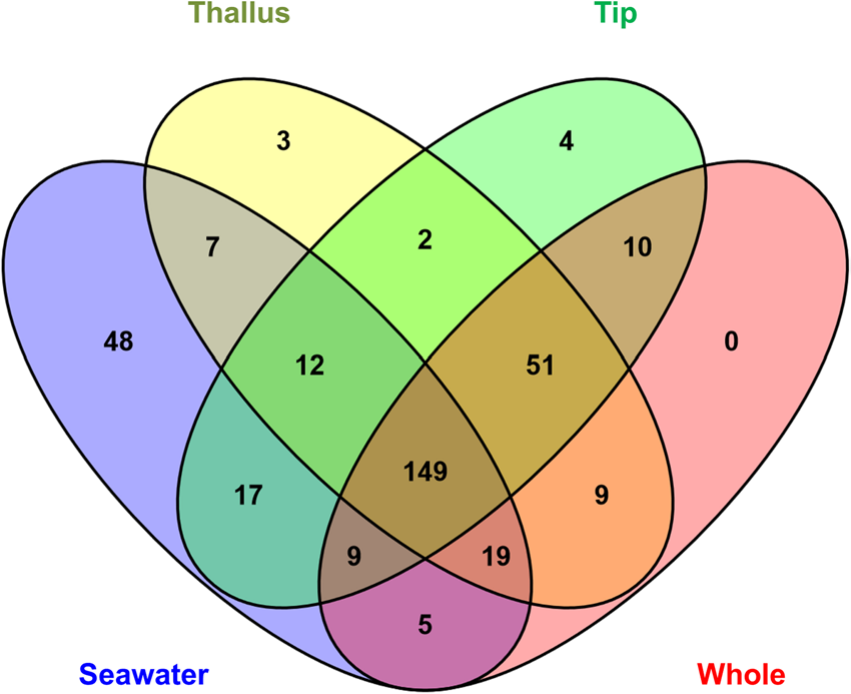
Venn diagram comparing the OTUs detected in all replicates. The Venn diagram was calculated with the online tool VENNY v2.1. The data matrix used for constructing this Venn diagram was a presence/absence of all 345 OTUs in the four respective samples (Thallus, Tip, Whole seaweed and Seawater samples).

The surface microbiome of the tips, thallus and the whole algal surfaces (incl. both tip and thallus regions) of *F*. *vesiculosus* was analysed comparatively by amplicon sequencing. Seawater was used as reference. Sequencing the V3-V4 hypervariable region of the bacterial 16S rRNA gene of the samples yielded 61226 (Tip#2) to 66230 (Tip#3) raw sequence reads per sample. Initial filtering resulted in 30518 (Tip#2) to 33011 (Tip#3) read pairs and between 1965 (Seawater#1) and 4060 (Whole seaweed#1) contigs passed several subsequent filtering steps. Filtered contigs were transformed to relative abundances and binned into operational taxonomic units (OTUs) at a similarity level of 97%, which generated 345 unique OTUs (Table S1). The microbial community comprised 13 bacterial phyla, divided into 18 classes (Fig. 1, Table S2). The majority of microbial OTUs (relative abundances) in all samples were assigned to Alphaproteobacteria. In *F*. *vesiculosus*, 64% to 68% of OTUs represented Alphaproteobacteria and their share in the seawater bacterial community was even higher (84%). Consequently, alphaproteobacterial families showed the highest relative abundance, also at family level, namely Rhodobacteraceae (21–27%), Erythrobacteraceae (10–31%) and Hyphomonadaceae (8–20%) in the seaweed samples (Table S3), while Rhodobacteraceae (35%) and Pelagibacteraceae (31%) were most common in the seawater reference (Table S3). At the genus level, we identified representatives of Erythrobacter and Altererythrobacter, as well as several members of the ubiquituous marine Roseobacter clade of the Alphaproteobacteria. The family Pelagibacteraceae was represented only in seawater samples mainly by an OTU affiliated to *Cand*. *Pelagibacter ubique*, the only currently cultivated member of the SAR11 cluster that reportedly dominates marine surface waters^35,36^. According to the relative abundances, Planctomycetia that represented the second most abundant bacterial group were particularly common in tip (11%) and whole seaweed samples (8%). The 10 Planctomycetia OTUs were affiliated mostly to yet unclassified genera (5 OTUs) but also to the genera *Algisphaera* (3 OTUs), *Bythopirellula* and *Gimesia* (1 OTU each). Cyanobacteria were also more pronounced in *F*. *vesiculosus* surface microbiome (9–13%) than in seawater samples (6%). Both calculated Shannon (H) and Simpson’s (1-D) diversity indices indicated a higher bacterial diversity in the surface microbiome (H: 4.01–4.1, 1-D: 0.95–0.96) than in the seawater (H: 3.5, 1-D: 0.89), but no notable difference among tips, thallus or whole seaweed sample was observed (Fig. S1). Comparative and statistical analysis of the microbiome samples via principal coordinate analysis (PCoA, Fig. 1) and PERMANOVA corroborated the findings of the amplicon sequencing above. The PCoA showed a close clustering of all *F*. *vesiculosus* surface microbiome samples and a clear differentiation from seawater reference. Permutational analysis of variance (PERMANOVA) analysis, i.e. the comparison of algal surface/seawater yielded a highly significant (p < 0.01) F value (16.24). However, no significant differences in the associated bacterial community were observed between the alga-derived samples (tip, thallus, whole seaweed) (F: 1.18–4.84, *p* > 0.05). Seventy-nine OTUs were exclusively obtained from *F*. *vesiculosus* surface (Fig. 2, Table S4), while the seawater samples contained 48 exclusive OTUs. Five OTUs (50: Deltaproteobacteria, 92: *Parvularcula flava*, 157: Synechococcales, 161: Haliscomenobacteraceae, 205: Saprospirales) were detected in all *F*. *vesiculosus* surface samples but not in the seawater sample (Table S4). Erythrobacteraceae showed elevated abundances (relative) in thallus samples, while Hyphomonadaceae and Planctomycetaceae were more abundant on the surface of the tips. A similarity percentage (SIMPER) analysis showed several OTUs to contribute equally to the bacterial community composition differences between the tip and the thallus surface as well as the difference of the latter to seawater samples by 10–15% each (Table S5). To improve the robustness, SIMPER analysis was repeated on the higher taxonomic level of bacterial classes. Alphaproteobateria and Planctomycetia accounted for the major variation between almost all sample types (>40%, Table S6) except for that between thallus/whole seaweed samples compared to the seawater sample. The differences between those (thallus/whole seaweed and seawater) are related to the abundance of Alphaproteobacteria and unclassified Cyanobacteria, instead of Planctomycetia (Table S6).

**Figure 3:**
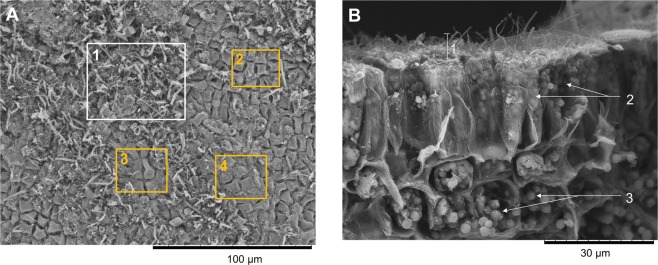
SEM image of (**A**) Patchy distribution of the morphologically variable surface-associated microbiota (white box 1) with small surface areas not covered by the biofilm (orange boxes 2, 3, 4, magnification: 1000×), and (**B**) Cross-section showing algal epidermis cells covered by epibionts. 1. Microbial biofilm thickness was measured to be ca. 10–15 µm (magnification: 2000×). 2. Elongated epidermal cells partly filled with plastids. 3. Plastids in epidermal and sub-epidermal cells.

**Figure 4:**
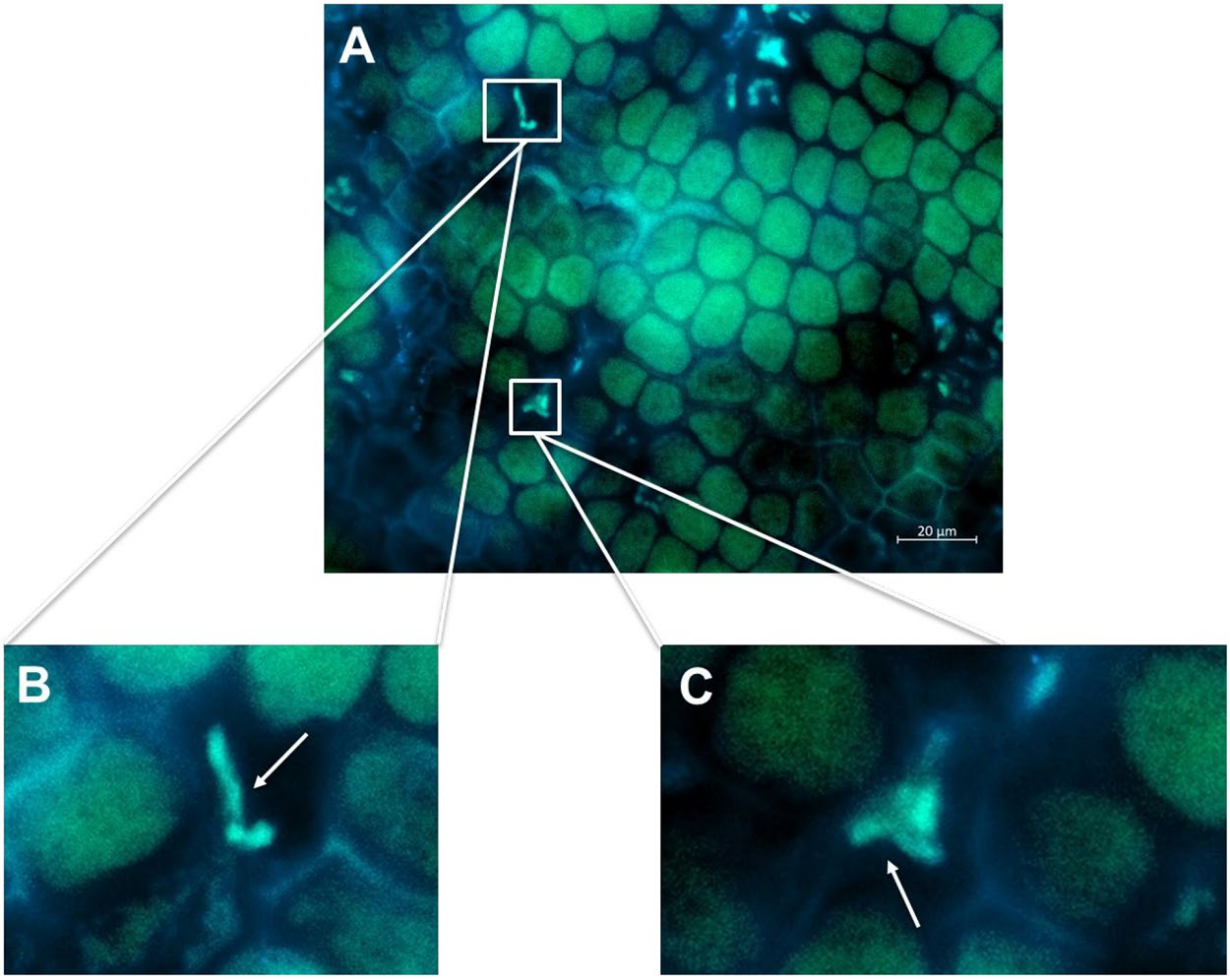
(**A**) Epifluorescence microscopic images of *F*. *vesiculosus* surface using biotin-labelled probes for CARD-FISH targeting the fungal class Eurotiomycetes (probe Eur1108). Composite image of eGFP and DAPI filters. (**B**,**C**) Close-ups of fungi marked by white boxes in A. White arrows point to the cells specifically stained by the respective probes. Scale bar: 20 µm.

Scanning electron microscopy (SEM) was used to analyse the density of the surface microbial biofilm. It showed a lower biofilm thickness at the uppermost tip than at the older thallus regions (Fig. S2A). A dense biofilm with a thickness of ca. 10–15 µm was apparent already approx. 100 µm below the tip region (Figs S2A and 3B). Various bacterial morphologies (cocci, rods) as well as pennate diatoms and long unidentified filaments were observed in the biofilms (Fig. S2B). A few small regions appeared to be free of epibionts (Fig. 3A) indicating the patchiness of the biofilm. We further investigated the surface microbiome by CARD-FISH to obtain spatial information of the phylogenetic composition of the microbial groups present on the surface (Fig. 4 for fungi of the class Eurotiomycetes, Fig. S3A for Actinobacteria, S3B for Firmicutes and S3C for Alphaproteobacteria). No surface area-specific distribution of the investigated phylogenetic groups was observed by CARD-FISH, exemplified by abundant representatives of Alphaproteobacteria that were distributed evenly on the thallus surface (Fig. S3C). Beyond the latter, we detected only few sparsely distributed Actinobacteria (Fig. S3A) and Firmicutes (Fig. S3B), supporting the findings from the amplicon sequencing (Fig. 1, Table S1). Moreover, CARD-FISH revealed positive signals for fungi of the class Eurotiomycetes (Fig. 4). CARD-FISH controls using pure cultures of Actinobacteria, Alphaproteobacteria, Firmicutes, Eurotiomycetes and Saccharomycetes (negative control for EUR1108 probe) showed no false-positive staining (Fig. S4). This is the first application of CARD-FISH imaging for surface analysis of *F*. *vesiculosus* and the first detection of fungi on algal surfaces by this technique.

### UPLC-MS/MS analysis of all *F*. *vesiculosus* extracts

Since no surface extraction method is capable of extracting the total surface metabolome and this study aimed at an untargeted metabolomics approach, two different surface extraction methods were used to maximize the numbers of metabolites in extracts. The ‘dipping’ method involved immersion of the algal fronds into a constantly stirred *n*-hexane:MeOH mixture (1:1, v/v) (FVAI) for 4 sec^13,16,30,31,37^. The second method is based on the adsorption of the surface exometabolites on a solid-phase C-18 material, followed by extraction with MeOH (FVAII)^17^. The whole (untreated) algal extract (FVC) was obtained by extracting the whole seaweed biomass with MeOH. Intactness of the algal epidermis was monitored by means of SEM. No damage or structural differences in density or thickness of the microbial biofilm was observed after extractions (Fig. S5) confirming the non-destructive nature of both surface extraction techniques. We also prepared ‘surface-free’ methanolic extracts, separately, from the residual alga after the surface treatment by ‘dipping’ (FVBI) or C-18 adsorption (FVBII). They served as a ‘control’ to assess the residual surface metabolites after surface extraction.

**Figure 5:**
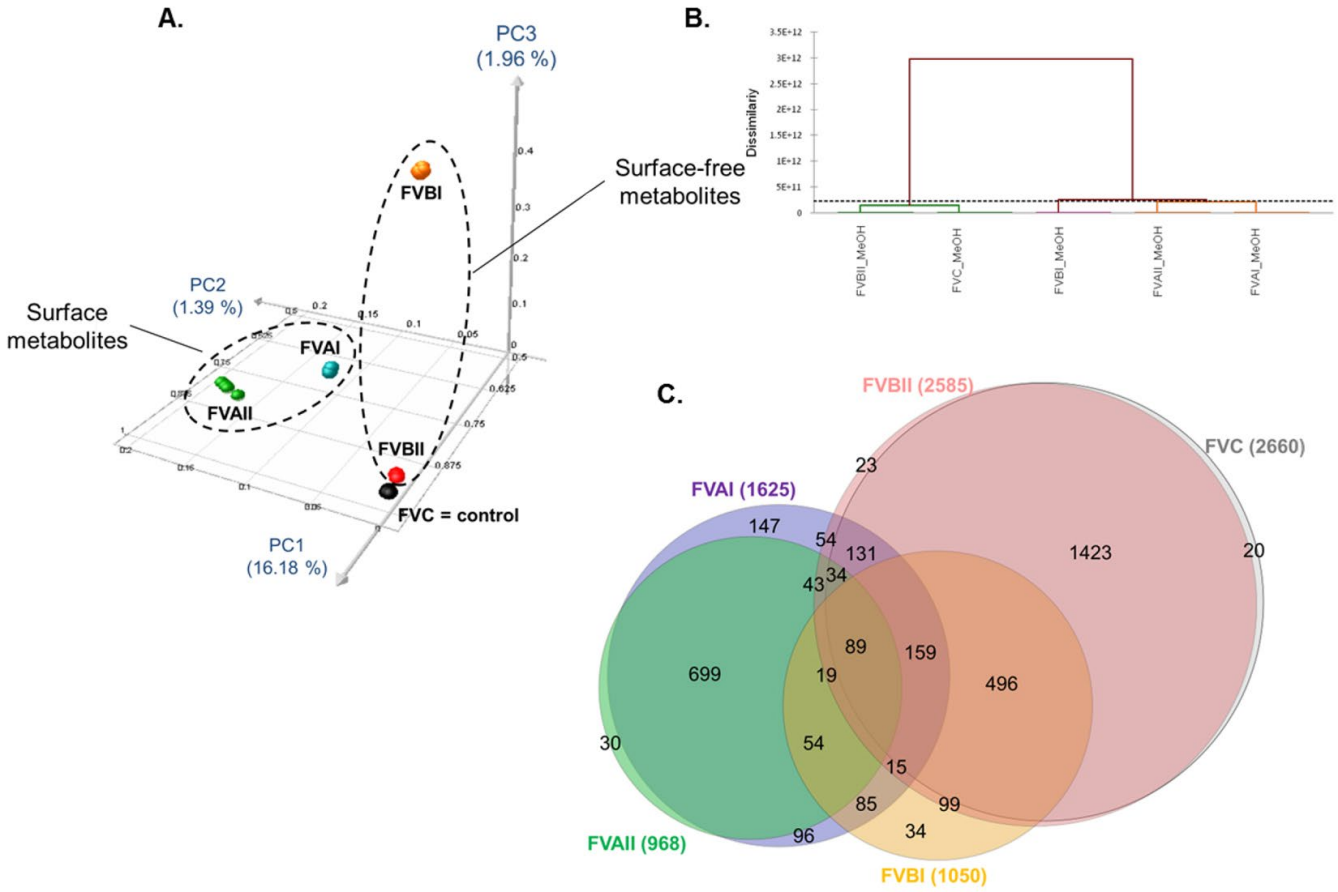
(**A**) 3D-PCoA plot and (**B**) Hierarchical Ascendant Classification (HAC) of the *F*. *vesiculosus* extracts based on LC-MS data. (**C**) Euler diagrams of unique and shared MS/MS spectra between different extraction methods. FVAI: Surface solvent extract. FVAII: Surface C18 extract. FVBI: Surface-free extract after solvent dipping. FVBII: Surface-free extract after C18 adsorption. FVC: Untreated (i.e. whole algal) extract. Numbers present on circle’s edges correspond to the smaller intersection areas between samples (e.g. 20 on the right corresponds to nodes specifically found in FVC).

An UPLC-QToF-MS/MS based metabolomics study (in positive ionisation mode) was conducted on each extract using four technical replicates. Figure S6 shows the comparative base peak chromatograms of all extracts. To visualise the differential global metabolomic profile of the extracts, the MS data were used to generate a 3D PCoA plot and Hierarchical Ascendant Classification (HAC) analysis (Fig. 5A,B). The PCoA and HAC showed that the surface extracts (FVAI and FVAII) form moderately related clusters indicating limited similarities of the metabolic signatures. The surface extracts clustered distantly from the whole *F*. *vesiculosus* (FVC) and the surface-free (FVBI, FVBII) extracts (Fig. 5A,B). The surface-free extracts FVBI (after solvent dipping) and FVBII (after C-18 extraction) were dissimilar, with the latter showing a similar profile to that of FVC (Figs 5A,B and S6).

**Figure 6:**
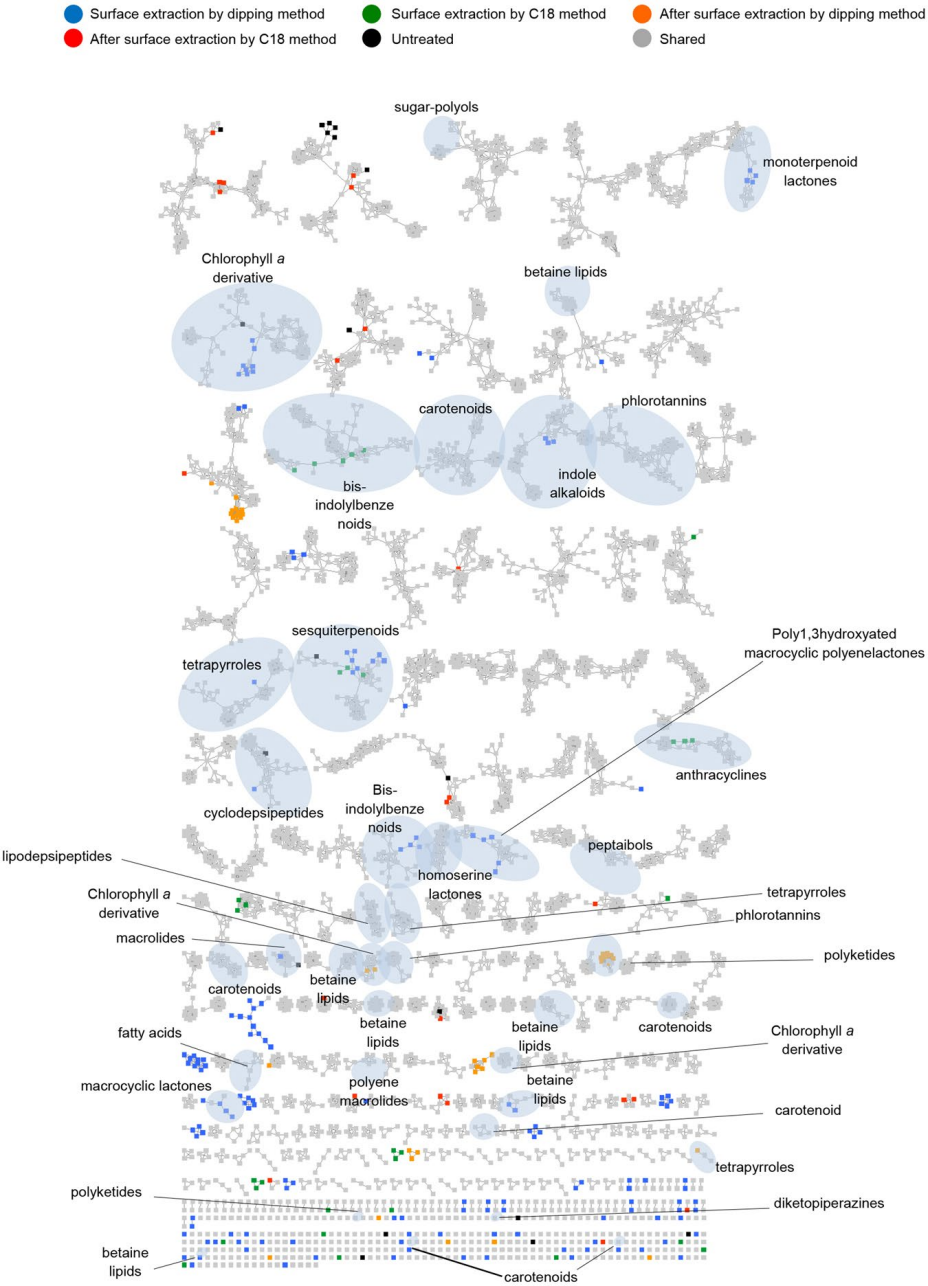
Visualization of the metabolome of different *F*. *vesiculosus* extracts by molecular networks: Putatively known metabolites were dereplicated and highlighted. Node colours represent different extracts (Blue: FVAI, Green: FVAII, Orange: FVBI, Red: FVBII, Black: FVC and Grey: shared metabolites). Nodes originating from solvent blanks (i.e. MeOH or hexane:MeOH mixture that was evaporated, dissolved in MeOH and injected to UPLC-MS/MS under the same conditions as the algal extract samples) were subtracted.

In order to annotate the individual metabolome of each extract, we performed a global molecular network (MN) analysis based on LC-MS/MS data using the GNPS platform^25^ combined with an *in silico* MS/MS database of the Universal Natural Product Database (ISDB-UNPD)^34^. This automatic dereplication was complemented by manual dereplication using multiple databases. The global MN analysis of all *F*. *vesiculosus* extracts consisted of 3750 nodes, which resulted in 656 clusters, after subtraction of background nodes. Overall, 39 chemical clusters belonging to various primary and secondary metabolite classes were annotated (Fig. 6 and Table S7). In total, 50 metabolites belonging to 21 chemical families were putatively identified (Figs 6, S7 and Table S7). Thirty metabolites were putatively annotated at compound level while the remaining 20 (mostly complex lipids) were only annotated at chemical family level based on their fragmentation pattern similarities using the analogue search function in GNPS^25^. Although our integrated dereplication effort improved annotation rate, many ions belonging to known and unknown clusters remained unannotated (Fig. 6).

The Euler diagram (Fig. 5C) generated from the MN showed similarities to the PCoA and HAC (Fig. 5A,B). With similar numbers of nodes, the FVC (2660 nodes) and FVBII (2585 nodes) represented the most diverse chemistry. The surface extracts FVAI and FVAII contained a total of 1625 and 968 nodes, respectively, with 243 nodes being exclusive to FVAI and 30 being exclusive to FVAII (Fig. 5C). 938 nodes were in common to both surface extracts. FVAI shares 413 nodes with FVC while FVAII extract has only 123 nodes common with FVC. The Euler diagram revealed 2095 nodes to originate from the algal tissue (i.e. nodes not shared with both surface extracts) while 972 being specific surface metabolites (Fig. 5C). These results suggest that the number of the metabolites present in inner tissues exceed 2-fold of that found in the surface extracts.

As for the surface-free extracts, 1050 and 2585 nodes were detected in the FVBI and FVBII extracts, respectively. FVBI shared 421 nodes with both surface extracts, 877 with FVBII and 812 nodes with FVC. FVBII shared approx. 544 nodes with both surface extracts.

### Putative metabolite annotation of *F*. *vesiculosus* extracts

The simple putative sugar mannitol was present in all extracts, including both surface extracts (Table S7). In total 37 metabolites were putatively dereplicated from the surface extracts FVAI and FVAII, 21 being common to both extracts, namely phospholipids (8, 12, 14, 20–23, 26), sulfoglycolipids (16, 17, 19, 24), the chlorophyll pigment 4,5-secopyropheophorbide *a* (30) and various SMs, i.e. peptaibol emerimicin IV (7), bis-indolyl benzenoid ochrindole C (13), anthracycline obelmycin F (25), lipodepsipeptide acremolide D (29), macrocyclic lactone filipin II (31) and mycoticin B (34), carotenoid fucoxanthinol (32) and phlorotannin hydroxyfucodiphlorethol (47) (Table S7, Fig. S7). Several secondary surface metabolites (1, 3, 32, 47, 49) were previously isolated from macroalgae including *F*. *vesiculosus*. This may indicate that the biosynthetic producer is the seaweed that exudes these SMs onto surface. Thirty-three compounds were annotated in FVAI dipping extract, five of which being exclusive to FVAI, i.e. monoterpenoid lactone loliolide (or epiloliolide) (3), betaine lipid (28), macrocyclic lactone bahamaolide A (33), bis-indolyl benzenoid ochrindole B (35), and fatty acid amide palmitamide (37) (Table S7, Fig. S7). Altogether 26 metabolites were identified, putatively, in the C18 surface extract (FVAII), some of which were absent in FVAI, e.g. (N-(3-oxooctanoyl) homoserine lactone (4), polyketide dihydrosorbicillin (38), the linear aminolipid stockerine (49) and the fungal alkaloid pestalamide B (9) (Table S7). The non-polar nature of these compounds may underlie their preference for the C18 material that is also highly lipophilic. Among all putatively annotated surface SMs, 16 metabolites were traced back to a putative fungal (7, 9, 13, 29, 35, 38, 39, 43) or bacterial origin (4, 5, 6, 15, 25, 31, 33, 34).

The UPLC-MS/MS analysis of the whole algal extract FVC enabled putative annotation of altogether 17 compounds. With eight putatively identified members, the most dominant natural product class identified in the FVC extract was betaine lipids. In the case of the betaine lipid 44, the MS/MS data comparison permitted us to annotate it as a DGTS/A potentially containing two myristic acid esters (C14:0) in *sn*−1 and *sn*-2 positions of the glycerol backbone (Table S7, Fig. S7). The remaining whole algal metabolites included phlorotannins (2), carotenoids fucoxanthin (40) and its dehydrated derivative (27, which was identified based on its MS data corresponding to a loss of a H_2_O molecule) and the porphyrine derivative pheophorbide *a* (42). One bacterial metabolite indolmycin (5) and three fungal metabolites, i.e. pestalamide B (9), acremolide D (29) and scopularide A (39) were also putatively identified from the FVC extract.

There was low overlap between the putatively identified metabolites in total algal and surface extracts. Altogether six putatively annotated FVC metabolites were common to both total and both surface-free extracts. Five putatively annotated surface metabolites were shared with FVAI, i.e. mannitol (1), indolmycin (5), betaine lipid (18), fungal lipodepsipeptide acremolide D (29) and cyclodepsipetide scopularide A (39). Besides mannitol (1), the fungal alkaloids pestalamide B (9) and acremolide D (29) were the only metabolites identified in both FVC and FVAII surface extracts. The only metabolite exclusive to FVC extract was the linear sesquiterpenoid farnesylacetone (36), some derivatives of which were previously isolated from brown algae^38^.

Altogether 23 different metabolites were putatively annotated in the surface-free extracts (15 in FVBI and 16 FVBII) (Table S7). Eight compounds were common to both extracts, including mannitol (1), a phlorotannin (2), three betaine lipids (18, 44, 46), the carotenoids (40, 27) and a porphyrine derivative (42) (Table S7, Fig. S7). Notably, all eight compounds were also detected in the FVC extract. The porphyrine derivatives 10S-hydroxypheophytin *a* (41) and pheophytin *a* (50) were exclusive to FVBI only. No metabolite unique to FVBII was putatively identified, but eight FVBII metabolites (5, 10, 11, 15, 29, 43, 45, 48) were absent in FVBI. Surface-free extracts also shared metabolites putatively identified from surface extracts (Table S7).

### ^1^H-NMR analyses on the surface extracts

^1^H-NMR analyses were performed to inspect the presence of the nine previously reported surface-associated compounds^11,17,30,31,37^ that were not detected in our surface extracts by LC-MS. The water and MeOH-soluble portions of the both surface extracts were analysed individually by ^1^H-NMR spectroscopy (Fig. S8). The comparison of the ^1^H-NMR spectra of the surface extracts with those of the commercially available standard compounds (alanine, proline, serine, threonine, citric acid and fucoxanthin) and the predicted ^1^H-NMR spectrum of DMSP, hydroxypropanoic acid and β-*D*-galactofuranose (Fig. S8) reconfirmed the absence of these compounds in both surface extracts.

Fucoxanthin has been reported as a surface associated metabolite of *F*. *vesiculosus*. In the current study, we detected fucoxanthin only in the surface-free (FVBI and FVBII) and the whole algal (FVC) extracts by LC-MS (Fig. S9, Table S7). Both surface extracts lacked fucoxanthin, which is in accordance with its subcellular location (i.e. chloroplast). Fucoxanthin, which is produced by the alga and released into the surface^39^ was reported to occur on thalli in larger concentrations (0.7–9 μg/cm² range)^30,31^. Similar amounts of fucoxanthin (40) were quantified in this study by UPLC-MS/MS (in mg/g of dried seaweed material), i.e. 1.09 ± 0.02 mg/g (FVBI), 1.19 ± 0.04 mg/g (FVBII) and 1.22 ± 0.07 mg/g (FVC) (Fig. S9), corresponding to surface concentrations of 5.86 µg/cm² (FVBI), 7.4 µg/cm² (FVBII), and 7.04 µg/cm² (FVC), respectively^31^. Interestingly, fucoxanthinol (32), the deacetylated form of fucoxanthin was detected and putatively annotated in both surface extracts.

The discrepancy regarding the absence of these nine metabolites in our surface extracts prompted us to perform a DESI-IMS analysis to further investigate the presence and spatial distribution of the putatively annotated surface (and internal) metabolites of *F*. *vesiculosus*.

### Localisation and spatial distribution of the surface-associated and inner tissue metabolites by DESI-IMS

Regular surface analyses by DESI-IMS require flat and preferably hard surfaces^20,40,41^. *F*. *vesiculosus* contains air bladders, receptacles and an elevated midrib rendering its surface non-flat. Therefore, the spatial distribution of the surface and the inner tissue metabolites across surface tips and thalli were investigated after preparing surface imprints and cross-sections by DESI-IMS (Fig. S10) using a MeOH:H_2_O (98:2) mixture as spray solvent. As the DESI-IMS data are voluminous, only the major and putatively annotated metabolites are presented here. The DESI-IMS analysis of the surface imprints (tips and thalli) revealed the presence of in total six major small molecules that could not be annotated to any compounds in any databases used (*m/z* 146.118; 173.081; 217.108; 233.081; 261.134 and 277.108) (Figs S11 and S12). Some of these metabolites, detected in positive mode, are located at the “lamina margin” (e.g. *m/z* 173.081) or in the “middle lamina” (e.g. *m/z* 277.108 and 146.118) regions of the tips and thalli, while others (e.g. *m/z* 233.081, 217.108 and 261.134) show a broader distribution (Figs S11 and S12).

**Figure 7:**
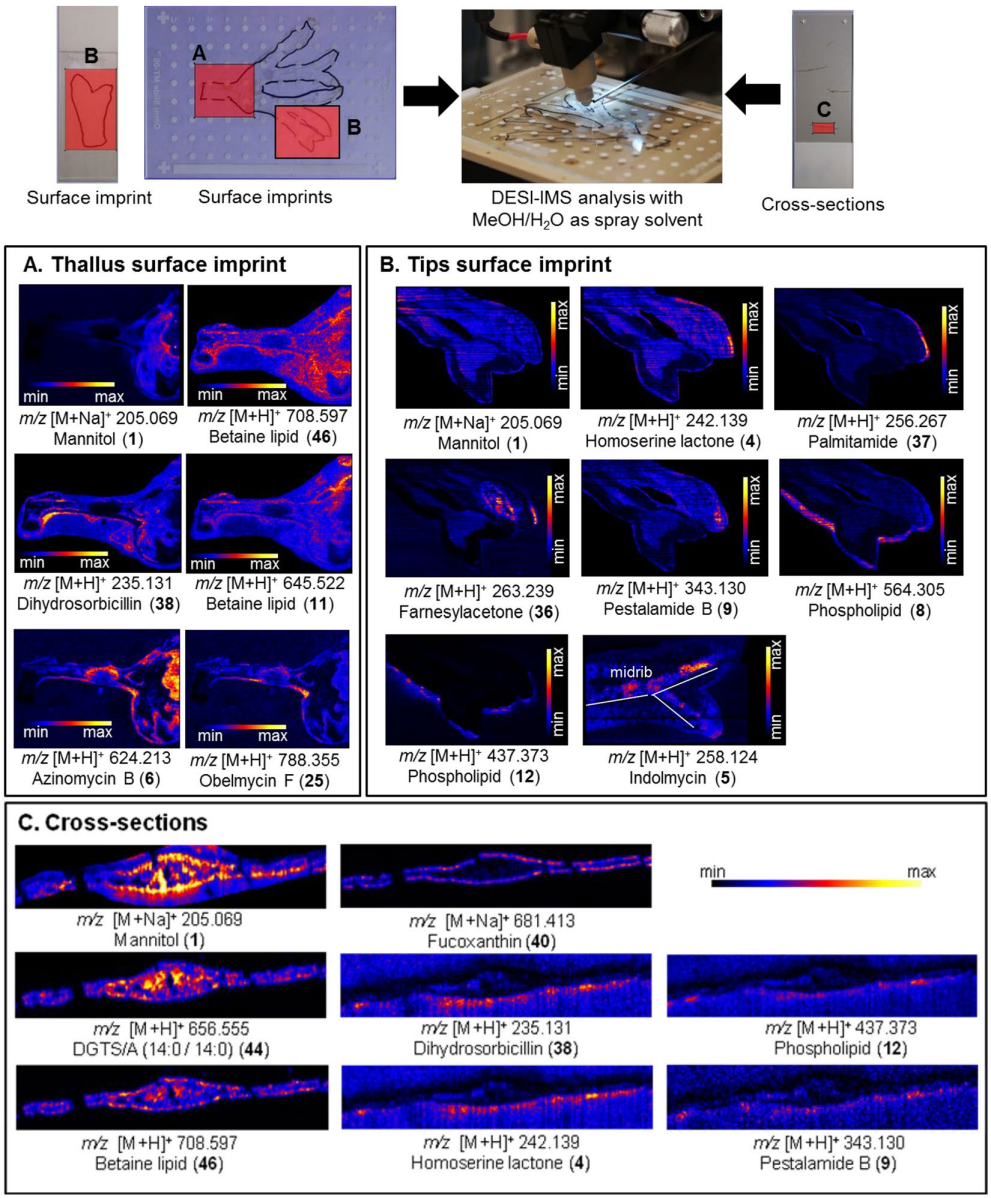
Spatial distribution of the putatively annotated metabolites localized on the surface imprints of (**A**) thallus, (**B**) tips and (**C**) on cross-sections of *F*. *vesiculosus* by DESI-IMS in positive ionization mode. Numbers correspond to annotated compounds reported in the Table S7.

In addition to these major unknown metabolites, DESI-IMS also revealed the spatial distribution of 13 less abundant surface metabolites, which were positively annotated by LC-MS^n^ (Fig. 7 and Table S7). Only mannitol (1) was observed both in thallus and tip surface imprints, showing a low intensity in the tip regions but a higher accumulation around the mid thallus region (Fig. 7A,B). DESI-IMS allowed visualisation of altogether six putatively annotated metabolites (1, 6, 11, 25, 38, 46) on the thallus surface imprints (Fig. 7A). The betaine lipid (46) was spread all over on the thallus surface. The other betaine lipid (11) and particularly the polyketide azinomycin B (6) showed high concentrations on the upper thallus, while the microbial metabolites 25 and 38 exhibited a higher presence in the basal part (Fig. 7A). Eight metabolites (1, 4, 5, 8, 9, 12, 36, 37) were mapped specifically on the tips of the surface imprints (Fig. 7B), four (4, 8, 12, 37) being observed mainly on the lamina margin, two on the lamina (9, 36), one on the midrib (5) and mannitol at the lower tip region (Fig. 7B). Six out of the 13 putative surface metabolites observed by DESI-IMS have been previously reported from bacteria (4–6, 25) and fungi (9, 38) (Fig. 7A and B).

In order to provide information on the spatio-chemical distribution of *F*. *vesiculosus* internal tissue metabolites, direct DESI-IMS analyses on algal cross sections were performed. A small algal piece was sectioned using a cryostat to reveal the spatial distribution of 8 metabolites previously annotated by UPLC/MS (Fig. 7C). Mannitol (1) and the betaine lipid DGTS/A (44) showed a broad distribution across the inner tissue with a higher intensity around the midrib and medulla (Fig. 7C). The second betaine lipid (46) showed a narrower and less abundant distribution mainly along the midrib medulla, the epidermis and meristoderm. The carotenoid pigment fucoxanthin (40) was clearly aligned on the epidermal cells (Figs 7C and S8). Interestingly, four metabolites (4, 9, 12, 38) previously observed on the surface imprints of the tips and thallus appeared to be diffused from the epidermis to the surface (Fig. 7C). Six compounds (1, 4, 9, 12, 38, 46) were putatively identified in both imprint and cross section analyses.

## Discussion

A few early studies investigated the surface-associated microbiota of *F*. *vesiculosus* (collected in spring in the North Sea^32^, or in autumn in the Kiel Fjord^33,42^ or cultivated in aquaria in North Sea water^33^) by using Sanger sequencing of excised DGGE bands^5^, 454 pyrosequencing^33,42^ and Illumina MiSeq^32^, using different hypervariable 16S rRNA gene regions V1-V2^32,33,42^, V3^5^, and showing *F*. *vesiculosus* surfaces to be rich in Proteobacteria (esp. Alphaproteobacteria) and Bacteroidetes^32,33,42^. We used *F*. *vesiculosus* samples collected in July based on the literature reporting the highest fouling pressure and the host-mediated fouling reduction strength to be in this month^16^. Herein we used Illumina MiSeq amplicon sequencing of the widely applied 16S rRNA V3-V4 hypervariable gene region of the bacterial epibionts. V4-based phylogeny reflects the full-length 16rRNA tree topology best^43^ and was therefore together with V3 selected for microbiome analysis. The current study revealed the surface microbiome of tip and thallus to be dominated by the Gram-negative Alphaproteobacteria (families Rhodobacteraceae, Erythrobacteraceae), which was confirmed by CARD-FISH analysis (Fig. S3C). All Rhodobacteraceae OTUs identified to genus level were affiliated to the ubiquitous marine Roseobacter clade, of which some representatives are known to produce AHL^44^, or have the genetic repertoire to produce a variety of SMs (NRPS or PKS genes^44^ or DMSP degradation^45^). Other detected bacterial phyla such as Bacteroidetes, Cyanobacteria and Planctomycetes are also commonly reported from marine macrophytes including *F*. *vesiculosus*^32,33,46^. In this study, Planctomycetes were the second most abundant phylum, which is in accordance with the associated microbiota of other brown algae *F*. *spiralis*, *Laminaria* sp. and *Sargassum muticum*^47^. Planctomycetes are known for their involvement in degradation of organic matter of plant and algal cell walls such as *L*-fucose, *L*-rhamnose, mannitol and sulfated polysaccharides^48^. Planctomycetes, a phylum still under taxonomic revision and currently classified as Gram-negative bacteria^49^, are present on a variety of macroalgae and may also be involved in degrading polysaccharides from the extracellular matrix of microbial biofilms. The high proportion of Planctomycetes observed herein however conflicts with the previous studies on Baltic Sea *F*. *vesiculosus*^32,33,42^, where Proteobacteria represented the most frequently detected OTUs, along with the second most abundant phyla Actinobacteria and Bacteroidetes. The low abundance of Actinobacteria in our samples was corroborated by CARD-FISH analysis (Fig. S3A). It remains to be proven, whether these differences are related to spatial and temporal variability, or are due to technical variation.

The surface microbiome showed a clear differentiation and higher microbial diversity in comparison to seawater microbiome (Figs 1 and S1), thus further supporting the hypothesis of host-specific microbial associations. We only detected 5 common bacterial OTUs among all algal samples that were absent in the seawater. This is similar to previous findings of Burke *et al*.^6^ who identified only 6 core OTUs present consistently among different individuals of the green alga *Ulva australis*. Such low numbers of common OTUs speak against core microbial-algae associations on OTU level but provide further support for the concept of higher core microbial taxa such as phyla or classes as already proposed by Egan *et al*.^3^. Although statistically insignificant, the surface microbiome analysis showed a lower bacterial diversity on two of three tip samples. This may indicate that bacteria have just been freshly recruited from the seawater^46^ and the observed differential microbiome may reflect different stages of initial bacterial colonization of the tips. SEM analysis also revealed a small region at the algal tip being almost free of epibionts (Fig. S2A). This region represents the youngest algal tissue harbouring the apical meristem that was exposed to fouling pressure for a shorter time than the older thalli^16^. Our SEM observations on older thallus surfaces (Figs 3 and S2B) are in line with previous studies^16^ reporting a dense but patchy prokaryotic assemblage consisting of cocci, diplococci and tetrads as well as filamentous cells and diatoms attached.

Current surface chemistry studies largely rely on solvent extraction that requires optimisation of critical parameters, e.g. the selection the solvent(s), exposure time and specific traits of the algal surface^18^ for the seaweed of interest. The impact of the solvent on the algal cell wall should be evaluated to avoid cell lysis^50^, many organic solvents and their mixtures have been used to specifically extract surface-associated metabolites^11,14,28,30,31,37^. In the present study, we used *n*-hexane:MeOH (1:1, v/v) mixture and dipping for 4 sec, which has previously been optimised for brown algae including *F*. *vesiculosus*^13,16,28^. For comparison, we performed a C18 solid phase adsorption as described by Cirri and coworkers^17^. The post-extraction visualisation of the algal surfaces by SEM did not detect any difference in the density of the microbial biofilm (Fig. S5) or damage on the algal cell walls, indicating the stability of the biofilm and the non-destructive character of surface extraction methods employed. Nevertheless, we observed significant amounts of residual C18 powder (easily distinguished by its larger particle size compared to the microbial cells) visible in SEM analyses. UPLC-MS analyses indicated, putatively, the presence of varying polarity of primary and secondary metabolites in both surface extracts, spanning from the very polar sugar polyol mannitol, middle polarity lipids (phospho-, sulfoglyco- and betaine lipids) to highly lipophilic carotene fucoxantinol. Among the dereplicated compounds (or classes), the primary metabolites (phospho- and sulfoglycolipids, but not betaine lipids) were mostly common to both surface extracts, but there were notable variations in their secondary metabolite constituents. A few additional highly lipophilic compounds were putatively identified in FVAII, including the surface signalling compound N-(3-oxooctanoyl) homoserine lactone (4), indicating the complementary nature of C18 absorption technique to solvent dipping.

Previous studies on *F*. *vesiculosus* surfaces have reported the presence of mainly primary metabolites, i.e. amino acids (alanine, proline, serine, threonine), sugars (mannitol, β-D-glucofuranose, uncharacterised sugar alcohols) and the organosulfur compound DMSP^30,37^. A recent GC-MS-based study describing temporal patterns in the chemical surface composition of *F*. *vesiculosus* showed the presence of citric acid, hydroxypropanoic acid and a number of uncharacterised mono- and disaccharides in the surface solvent extract^13^. Except for mannitol, our LC-MS, NMR and DESI-IMS studies failed to prove the presence of any of these metabolites in the surface extracts, although we applied the same surface extraction protocols^17,37^. To our knowledge, the only secondary metabolite surface reported from the surface *F*. *vesiculosus* is the carotenoid fucoxanthin, obtained by both dipping and C18 surface extraction^11,17,31^. Proline, alanine, DMSP and fucoxanthin were previously reported as antifouling metabolites to control the abundance and composition of fouling bacteria on *F*. *vesiculosus*^30,31,37,51^. The absence of DMSP on the surface may be attributed to the high abundance of bacteria affiliated to the Roseobacter clade of Rhodobacteraceae with known capacities to degrade DMSP^45^. Differences in the sampling period and/or sampling location, weather conditions (as 2017 was relatively cold and rainy throughout the whole year), or individual phenotypic plasticity may also contribute to these contradictory results. Moreover, our metabolomics study only represents a snapshot (one sample at a fixed time point) in comparison to, for example, the seasonal variation study performed by Rickert *et al*.^13^.

The MN-derived UPLC-MS/MS-based untargeted metabolomics study on *F*. *vesiculosus* extracts revealed a diverse and differential metabolome (Fig. 6). The application of manual and automatic dereplication efforts enabled putative annotation of 50 metabolites belonging to 21 chemical families (Fig. 6, Table S7). The surface extracts showed significant variations to whole algal extracts. The dereplication efforts indicated the surface extracts to possess a more diverse metabolome dominated by SMs, some of which are presumably of algal origin (see below). Putatively identified surface metabolome  also contained a higher number and diversity of SMs previously reported from marine/terrestrial bacteria and filamentous fungi. Only a few (putatively) identified compounds were common to both surface and whole tissue extracts. To our knowledge this is the first in-depth study applying untargeted metabolomics approach on any (algal) surface extract and the first metabolomics approach mapping the differential chemistry of the surface versus whole algal tissues. The only related study^14^ compared the chemical fingerprints of the surface extracts to those of pure compounds previously isolated from the whole tissue extract of the brown alga *Taonia atomaria*. In the current study, many compounds that were putatively identified by the UPLC-MS/MS based metabolomics were localised on algal surfaces or inner tissues by DESI-IMS. In few cases, DESI-IMS failed to detect compounds (e.g. 3, 30, 32, 47) annotated from the surface extracts by LC-MS. Their absence in DESI-IMS imprints can be explained either by their low concentration below the detection limit of the DESI, or by the observed patchiness of the biofilm leading to quantitative variations across the surface. To our knowledge, this is the first untargeted DESI-IMS study that spatially localised the surface and the inner tissue metabolites of a seaweed.

Detailed LC-MS and/or DESI-IMS studies putatively identified several compounds (e.g. mannitol, fucoxanthinol, phlorotannin derivatives) that apparently originate from the seaweed and released onto the surface of *F*. *vesiculosus*. The polyol sugar mannitol (1) was consistently identified in all algal extracts by LC-MS and on all surface imprints by DESI-IMS. In cross-section imaging (Fig. 7, Table S7) it showed a more intensive distribution in the algal midrib and medulla. Mannitol makes up 20–30% of the dry weight of brown algae^52^ where it plays manifold roles, i.e. carbon storage, osmo-protection and oxygen radical scavenging^53^. This sugar was previously reported from the surface extract of *F*. *vesiculosus*^16,28^, where it may represent a nutritional source especially for the members of the Planctomycetes that possess genetic capacity for degrading mannitol^49^. This fact may underlie the high abundance of Planctomycetes on the surface of *F*. *vesiculosus* as shown by our amplicon sequencing study and the low abundance of mannitol in surface imprints deduced by DESI-IMS.

Another *F*. *vesiculosus*-derived compound is fucoxanthin (40), a carotenoid with reported antifouling activity^31^. Although absent from the surface extracts, we detected fucoxanthin in the whole algal extracts. Fucoxanthin is a photosynthetic pigment occurring in the chloroplasts of brown macroalgae, in particular in the cellular tissues that are rich in plastids, i.e. epidermis and the cortical area, where the majority of photosynthesis occurs due to maximum light exposure at the ocean surface^54,55^. The DESI-IMS analysis of the algal cross-sections showed the clear distribution of fucoxanthin throughout the epidermal cells and its complete absence in surface imprints. Tandem MS analysis of the extracts also confirmed these results. The quantification of fucoxanthin in surface-free and whole algal extracts revealed approx. two-fold higher quantities (1.09–1.22 mg/g) in comparison to that obtained with the whole tissue *F*. *vesiculosus* acetone extract (0.696 ± 0.02 mg/g)^55^. Saha *et al*.^31^ reported fucoxanthin at a concentration range between 0.7 and 9 µg/cm² on the *F*. *vesiculosus* surface. This difference and its absence in both surface extracts can be attributed to seasonal and geographic variations, nutrient availability, exposure to sunlight, the extraction method and its duration (10 sec^31^. instead of 4 sec). Diatoms, including pennate diatoms, are known to be rich in fucoxanthin, hence diatoms attached to a more heavily fouled surface of *F*. *vesiculosus*^16^ may be the source of fucoxanthin isolated in the previous studies. Although fucoxanthin was absent, its diacetyl form fucoxanthinol (32) was detected in both surface extracts by UPLC-MS. Fucoxanthinol has previously been isolated from *F*. *vesiculosus* whole cell extracts^56^.

Additional compounds presumably of algal origin include phlorotannins that are common components of brown algae with multiple ecological functions^57–59^. In the current study we have putatively identified two phlorethol type phlorotannins, one from the algal tissue extracts (2), and another (47) from the surface extracts. Stockerine (49) is an interesting linear aminolipid reported from the brown alga *Stockeyia indica* together with loliolide^60^. Loliolide and epiloliolide (3) are very common monoterpene lactone norisoprenoids reported from many brown seaweeds, marine invertebrates, sediment as well as numerous terrestrial plants and animals (insects)^61^. These degraded lactonic carotenoids with a common occurrence in nature have never been reported from the genus *Fucus* before. Farnesylacetone (36) was the only compound detected exclusively in the whole algal extract. Farnesylacetone type linear sesquiterpenoids have been reported from Fucales^38^, thus it is likely that 36 has a truly algal origin.

In the surface extracts, we putatively annotated 16 metabolites that have been previously reported from marine or terrestrial bacteria and fungi. Many of microbial metabolites were also detected in imprints or cross sections by DESI-IMS. For example, N-(3-oxooctanoyl)homoserine-lactone (4) was detected in the surface (FVAII) and surface-free extract (FVBI) of *F*. *vesiculosus* by LC-MS and localised to the apical region of the tip lamina by DESI-IMS (Fig. 7). N-acyl-homoserine lactones (AHLs) are quorum-sensing signals in certain genera of Gram-negative bacteria that control the activation of genes involved in bacterial cell-to-cell communication, biofilm development, regulation and virulence^62,63^. Potential candidate for the production of the AHL (4) is the Roseobacter clade (Rhodobacteraceae), the most abundant bacterial family according to the results from the microbiome study. This study showed the surface epibiome of *F. vesiculosus* to be clearly dominated by Gram-negative bacteria, including Alphaproteobacteria and Planctomycetetes. However, our metabolomics work putatively annotated only one compound, N-(3-oxooctanoyl)homoserine-lactone (4), with a Gram-negative bacterial origin. This is possibly due to underexplored chemical machinery of Gram-negative bacteria, which is underrepresented in databases. Indeed, many of the surface metabolites putatively identified in the surface extracts and imprint images have their origin in Actinobacteria, e.g. *Streptomyces* sp. isolated from marine or terrestrial environments. The annotation of many streptomycete metabolites may be due to their over-representation in the databases. Our amplicon sequencing-based microbiome study indicated the low abundance of Actinobacteria, but it must be noted that amplicon sequencing and CARD-FISH can only give qualitative evidence on the presence and abundance of a phylogenetic group, but not on their metabolic activity. Application of specific probes directly targeting the identified genera or additional techniques such as BONCAT-FISH^64,65^ or direct-geneFISH^66^, can link phylogenetic identity and metabolic activity. Such approaches may be necessary to prove if the observed metabolites derive from the observed taxa, although their application in environmental samples remains yet to be proven.

The microbiome is not just composed of bacteria but also includes fungi. Indeed many fungal metabolites were putatively annotated from the surface extracts and successfully visualised by DESI-IMS in this study. CARD-FISH identified fungi from the class Eurotiomycetes on the algal surface confirming the potential production and putative presence of ochrindole type metabolites (35, 13) on surface extracts. The presence of fungal groups other than Eurotiomycetes on the algal surface is also very likely, since the observed CARD-FISH signals only reflect a proportion of the entire fungal community. Notably, some of the fungal metabolites had specific surface distribution. The antifungal alkaloid pestalamide B (9), previously isolated from the fungus *Pestalopsis* sp^67^, was distributed on the lamina surface tips imprints by DESI-IMS with a higher abundance on the apical tips region. The real fungal diversity associated with the *F*. *vesiculosus* surface could not be observed by CARD-FISH, as no suitable probes are currently available. Further quantitative and ecological studies are needed to understand the potential ecological role of fungal metabolites in surface microbial composition.

LC-MS/MS and DESI-IMS also mapped many surface-associated primary metabolites. The majority (12 out of 21) of the metabolites dereplicated commonly in both surface extracts belong to amphiphilic lipid classes, i.e. phospholipids and sulfoglycolipids that are major components of cell membranes of both macro- and microorganisms and play role in chemical signaling^68,69^. Two surface phospholipids (8, 12) were localized to the margin of the tip region by DESI-IMS (Fig. 7). Only two members (18, 28) of the third class of polar lipids, i.e. betaine lipids, were detected in surface extracts by LC-MS, while the majority were present in tissue extracts. DESI-IMS localised two of them (44, 46) to the central algal region (cross sections). The latter, together with the third betaine lipid (11) were also visualised on thallus surface imprints where 46 presented a broad distribution while 11 was more located on the upper part of the thalli (Fig. 7). Finally, we putatively identified a few molecules that have been reported from haptophyte type microalgae (kingdom Chromista). The fatty acid amide (FAA) palmitamide (37) detected in the surface solvent extract FVAI and on the tips by DESI imaging (Fig. 7) was previously isolated from *Prymnesium parvum*, and is known to be toxic to fish and other aquatic animals^70^. Fatty acid amides represent a large class of endogenous signalling lipids^70^. Further derivatives of pheophytins (41, 42), i.e. porphyrine type chlorophyll molecules lacking a central Mg^2+^ ion were identified in tissue extracts by DESI-IMS.

## Conclusion

In this study we combined several powerful and state-of-the-art techniques to analyse the surface microbiome and metabolome of the marine foundation species *F*. *vesiculosus*. The employment of amplicon sequencing, three different microbiological and chemical imaging techniques and a detailed untargeted metabolomics study revealed the rich and differential chemical composition of the surface and the tissue extracts of *F*. *vesiculosus*. For the first time bacteria and fungi were visualised directly on the surface of *F*. *vesiculosus* by CARD-FISH technique. Besides putative primary and secondary metabolites with algal origin, a number of putative bacterial and fungal secondary metabolites were mapped onto algal surface (or inner tissues) by DESI-IMS. This is the first study reporting an untargeted metabolomics approach applied to a surface extract and first comparative metabolomics study analysing different phylloid layers of a seaweed. This work provides valuable information on the surface microbiology and chemistry of the Baltic brown alga *F*. *vesiculosus* at µm resolution and may assist future chemical ecology studies.

## Methods

### Sample collection

*F*. *vesiculosus* was collected at Bülker Leuchtturm (54°27′15.6′N and 10°11′55.0′E, Kiel, Germany) in July 2017. The algal material was immediately placed in plastic bags with ambient seawater and rapidly transported to the laboratory in a cooling box. The samples were carefully rinsed with artificial seawater (1.8% Instant Ocean, Virginia, USA) to remove loosely attached macrofoulers.

### Solvents and reagents

Solvents for extraction, i.e. MeOH and *n*-hexane were HPLC grade and purchased from VWR (Pennsylvania, USA). UPLC/MS grade solvents (acetonitrile, MeOH and water) for sample preparation and LC-MS/MS analysis were from Biosolve (Dieuze, France). Ultra-purified water was obtained by an Arium water purification system (Sartorius, Germany). All standard compounds (i.e. proline, alanine, serine, threonine, citric acid and fucoxanthin) were purchased at Sigma Aldrich (Saint-Louis, Missouri, USA) and VWR (Dresden, Germany).

### Sampling for surface microbiome analysis

Samples were taken aseptically and prior to all other samples following a previously described protocol^32^. Briefly, water was sampled in sterile 1 L Schott glass bottles in immediate vicinity to the collected *F*. *vesiculosus* thalli (3 algal replicates). Before sampling, algal thalli were rinsed with seawater previously sterile-filtered through 0.2 µm cellulose acetate syringe filters (VWR International GmbH, Darmstadt, Germany). For sampling of algal surface-associated microbiota, approximately 15 cm^2^ of algal surface per replicate were swabbed using sterile cotton swabs. We sampled 3 different regions of the sporophyte, i.e. tip, thallus and the whole surface sample (incl. both tip and thallus regions). After sampling, all samples were immediately placed on ice and the cotton swabs were frozen at −80 °C upon arrival in the lab. 0.5 L of all 3 water samples were filtered through cellulose nitrate filters (pore size 0.45 µm, Whatman) and filters were immediately stored at −80 °C freezer.

### DNA extraction and 16S rRNA gene amplicon sequencing for microbiome analysis

Filters and cotton swabs (3 replicates for each sample type) were transferred to 5 mL PowerWater Bead Tubes (MoBioPowerWater Kit). 1 ml of Inhibitex buffer (QIAamp Fast DNA Stool Mini Kit, Qiagen) was added followed by a mechanical cell disruption step (2 × 10 min) using a bead-beating device (TissueLyzer, Qiagen). After centrifugation for 5 min (4000 × g, RT) the supernatant was transferred to a new 1.5 mL reaction tube. The lysate was further processed according to manufacturer’s instructions (QIAamp Fast DNA Stool Mini Kit, Qiagen). DNA concentration was determined by PicoGreen measurement (Quant-iT PicoGreen dsDNA Assay Kit, Thermo Fisher) and DNA integrity was checked by agarose gel electrophoresis. 16S rRNA gene amplicon libraries were prepared using a Nextera two-step PCR using the primers 341F_ill and 802R_ill^71^, for amplification of the 16S rRNA gene V3-V4 hypervariable region. Sequencing was performed on an Illumina MiSeq platform using the Illumina MiSeq Reagent Kit v2 (2 × 250 bp). Raw amplicon sequences are accessible in the Sequence Read Archive database of NCBI (Accession No. PRJNA508939).

### Bioinformatic processing

Amplicon sequences were processed with the bioinformatics tool set mothur v.1.39.5^72,73^. Filtered contigs (generated from demultiplexed and quality filtered reads pairs, removal of contigs with <10 bp read overlap or ambiguous bases) were aligned to a trimmed (V3-V4) and dereplicated in-house curated reference alignment consisting of 128118 bacterial 16S rRNA gene sequences. Several subsequent filtering steps were performed to remove artefacts or chimeric sequences by pre-clustering^74^ of properly aligned contigs and UCHIME v4.2.40^75^, respectively. Taxonomic sequence classification was enabled by comparison to a reference database (NCBI RefSeq Targeted Loci Project (Access Date: 2017–12–11) comprising 18659 bacterial and 897 archaeal 16S rRNA gene sequences amended with 119 mitochondrial and 96 chloroplast 16S rRNA gene sequences (extracted from: mothur RDP 16S rRNA reference (PDS) v16) to allow for contaminant removal. Taxonomic contig assignment was performed using the Wang method^76^ at a bootstrap threshold of 60% followed by removal of contigs not assigned to any bacterial taxon. Contigs were finally clustered into operational taxonomic units (OTUs) at 97% similarity level by using ‘OptiClust’^77^. Pre-binning of contigs according to their taxonomic classification (order level) was followed by removal of low-abundance OTUs using an in-house R script^78^. Subsequently, contig counts were transformed to relative abundances (based on the total number of contigs per sample), followed by ordering of OTUs by decreasing mean percentage across samples. OTUs with a cumulative mean percentage amounting to 95% were retained. To account for multiple 16S rRNA gene copies, we used rrnDB v5.4^79^ to determine a copy number norm factor (CNNF) for each filtered OTU, which was then applied to normalise contig counts of filtered OTUs. A filtered OTU table combining OTU taxonomy and per-sample counts was generated (Table S1).

### Statistical analysis of the 16S rRNA amplicon sequencing dataset

If not stated otherwise, the filtered OTU table was the basis for statistical analysis by Past3.12 software (https://folk.uio.no/ohammer/past/^80^). Alpha diversity was estimated by calculating Simpson’s (1-D) and Shannon’s (H) diversity indices. A PCoA plot based on a Bray-Curtis distance matrix was generated to visualise similarities between samples types. Statistical differences in the microbial community composition of *F*. *vesiculosus* and the water samples were assessed by one-way PERMANOVA (Bray-Curtis similarity index). Those OTUs (as well as the taxonomic rank family) contributing most to the microbiome differences were detected by SIMPER analysis (Bray-Curtis similarity index). Additionally, a Venn diagram showing shared OTUs among samples was created using the online tool VENNY v2.1 (http://bioinfogp.cnb.csic.es/tools/venny/^81^).

### Scanning Electron Microscopy (SEM)

Directly after sampling, small pieces were cut from the algal thalli using sterile forceps and scalpels and fixed in 4% formaldehyde. Prior to SEM analyses, samples were first subjected to an ascending ethanol series for dehydration (10 min each at 50%, 80%, 90% and 98% v/v EtOH). During critical point drying (CPD), CO_2_ was incubated for 5 min in the critical point dryer (E3000 Critical Point Drying, Quorum Technologies, New Haven, UK) in 8 cycles to achieve a serial displacement of ethanol. Afterwards, preparations were visualised in a TM3000 tabletop scanning electron microscope (Hitachi, Mannheim, Germany).

### Catalyzed Reporter Deposition Fluorescence *in situ* hybridization (CARD-FISH)

The CARD-FISH study was performed following a modified protocol from Pernthaler *et al*.^82^ using the following oligonucleotide probes: HGC236 for Actinobacteria, ALF968 for Alphaproteobacteria, Eur1108 for Eurotiomycetes and a combination of LGC354A,B,C for Firmicutes. 5′-Biotin labelled probes were purchased at biomers.net (Ulm, Germany). Immediately after sampling, samples for CARD-FISH were incubated for 3 h in 4% paraformaldehyde and then washed twice in 1 × PBS. Samples were then stored in 1:1 PBS/EtOH at 4 °C until further analysis. Further methodological details on CARD-FISH sample preparation are provided in the Supplementary Information. Prior to microscopic analysis, samples were counter-stained by 4′,6-diamidino-2-phenylindole (DAPI). For CARD-reaction TSA kit 22 (Thermo Fisher Scientific, Schwerte, Germany) was used. Microscopy was performed on a Zeiss Imager M2 epifluorescence microscope using DAPI and EGFP filter sets. Image acquisition was carried out with a Zeiss AxiocamMRm (Rev 2) using the filter sets for eGFP (488 nm/509 nm) and DAPI (358 nm/463 nm) and the ZEN 2.3 software package. To exclude non-specific probe binding, control experiments were performed using pure cultures of *Streptomyces globisporus* DSM 41647 (positive control for probe HGC236), *Bacillus subtilis* DSM 347 (positive control for probe LGC354A,B,C), *Gluconobacter oxydans* DSM7145 (positive control for probe ALF968), *Penicillium* sp. (positive control for probe Eur1108) and *Saccharomyces cerevisiae* DSM70449 (negative control for probe Eur1108). Further, all non-target strains were used as negative controls for all probes (Fig. S4).

### Preparation of the surface extracts

Two different extraction methods (solvent dipping and solid phase C18 adsorption) were used in parallel to recover the surface metabolites of *F*. *vesiculosus*. The solvent extraction was performed as described by Rickert *et al*.^13,16,28^. The *F*. *vesiculosus* thalli and tips (8 kg wet weight) were dipped into stirring *n*-hexane:MeOH mixture (1000 rpm, 1:1, v/v) for 4 sec. The organic extract was paper-filtered (cellulose, diameter: 250 mm, particle retention: 10–20 µm, VWR, Pennsylvania, USA) and evaporated to dryness under vacuum at 40 °C to give the so-called solvent dipping extract (FVAI) (4.5 g, 0.06% yield). The C18 surface extraction method employed the protocol reported by Cirri *et al*.^17^. Briefly, the *F*. *vesiculosus* thalli and tips (0.9 kg wet weight) were stirred for 2 min with C18 material (Sepra C18-E; 50 µm, 65A, Phenomenex, Torrance, USA) and left at RT for 10 min. The C18 material adsorbed on the algal surface was removed with artificial seawater (1.8% Instant Ocean, Virginia, USA) and packed directly into a glass column. It was first washed with artificial seawater, subsequently with MilliQ water (900 mL), and finally with 100% MeOH. The water phases were combined and stored at 4 °C. The MeOH phase was evaporated to dryness under vacuum to afford the so-called C18 surface extract (FVAII) (63.8 mg, 0.007% yield).

### Preparation of the whole algal extracts

Algal material was freeze-dried and ground by a pulverisette system. 100 g of algal powder was extracted with MeOH as described for the surface-free extracts above to afford the so-called whole (untreated) algal extract (23 g, 23% yield).

### Preparation of the surface-free extracts

The algal materials remained after both dipping and C18 solid phase surface extraction processes were first air-dried at ambient temperature. Subsequently they were separately freeze-dried and powdered by a pulverisette system (0.2 mm pore size; Fritsch, Idar-Oberstein, Germany). Each algal powder (100 g residue after solvent dipping and 140 g after C18 surface extraction) was separately extracted, successively, for 4 times overnight with MeOH (1:4 ratio, w/v) under continuous stirring (2000 rpm, IKA RH basic 2, Staufen, Germany). The filtered extracts were combined and evaporated under vacuum to afford the so-called surface-free algal extracts FVBI (15.7 g, 15.7% yield) and FVBII (25.32 g, 18.1% yield).

### LC-MS/MS analysis

The metabolome of the *F*. *vesiculosus* extracts was analysed by an Acquity UPLC I-Class System coupled to an Acquity UPLC-PDA detector and the Xevo G2-XS QTof Mass Spectrometer (Waters, Milford, MA, USA). Before injection, all extracts were dissolved in MeOH and filtered through 0.2 µm PTFE syringe filters (Carl Roth, Karlsruhe, Germany). Extracts were prepared at a concentration of 1 mg/mL and injected (0.6 µL) in quadruplicate (technical replicate) onto an Acquity UPLC HSS T3 column (High Strength Silica C18, 1.8 µm, 2.1 × 100 mm, Waters) operating at 40 °C. A binary mobile phase system (A: 0.1% formic acid in water UPLC/MS grade and B: 0.1% formic acid in acetonitrile) was pumped at a flow rate of 0.6 mL/min at the following gradient: initial, 99% A; 0–2.5 min, 60% A linear; 2.5–13.5 min, 0% A linear; 13.5–14.5 min, 0% A, following by washing and reconditioning of the column. Total run time was 18 min. The MS and MS^n^ spectra, in positive and negative mode, were recorded during the UPLC run with the following conditions: capillary voltage: 0.8 kV (positive mode) and 1 kV (negative mode), cone voltage: 40 V, source temperature: 150 °C, cone gas flow: 50 L/h, desolvation gas flow: 1200 L/h and a collision energy ramp with low collision energy: 6–60 eV and a high collision energy: 9–80 eV. Scan times were 0.1 sec and the acquisition range was *m/z* 50–1600. MassLynx Software (version 4.1) was used for data acquisition and analysis. Although the spectra were recorded in positive and negative mode, only the data obtained in positive mode has been taken into account based on their higher complex profiles. The Metabolomics4Workflow platform was used for the preprocessing of the LC/MS data^83,84^. The dereplication workflow available at the online GNPS platform (https://gnps.ucsd.edu/^25^) was combined with ISDB-UNPD^34^ and manual dereplication using Antibase 2012 (Wiley-VCH), Dictionary of Marine Natural Products^85^, MarinLit^86^ and Reaxys (https://new.reaxys.com) was used for the chemical identification of putative known metabolites.

A calibration curve for fucoxanthin^11,87^ was recorded by UPLC-MS/MS in order to quantify the amount of fucoxanthin present in surface-free and whole algal extracts. 8 different concentrations of fucoxanthin (0.1 µg/mL, 0.25 µg/mL, 0.5 µg/mL, 0.75 µg/mL, 1 µg/mL, 1.5 µg/mL, 2 µg/mL and 3 µg/mL) were injected in triplicate (0.6 µL) into the UPLC-MS/MS system using the same conditions as for analysis of the extracts. The peak intensity of the *m/z* [M + Na]^+^ 681.4134 corresponding to the fucoxanthin peak was extracted with MassLynx software and a calibration curve was calculated using MS Excel 2010 (*y* = 1e^6^*x* and R² = 0.995).

### Molecular network analysis

All MS data were converted to mzXML format using msconvert, a part of the ProteoWizard package^88^ and subsequently imported into the GNPS platform. Molecular networks, for positive ionisation mode, were created using the online workflow at GNPS. The data were filtered by removing all MS/MS peaks within +/−17 Da of the precursor *m/z*. MS/MS spectra were window-filtered by choosing only the top 6 peaks in an +/−50 Da window throughout the spectrum. The data were then clustered with MS-Cluster with a parent mass tolerance of 0.02 Da and a MS/MS fragment ion tolerance of 0.02 Da to create consensus spectra. Consensus spectra containing less than 2 spectra were discarded. Networks were then created where edges were filtered to have a cosine score above 0.5 and more than 4 matched peaks. Further edges between two nodes were kept in the network only if each of the nodes appeared in each other’s respective top 10 most similar nodes. The spectra in the network were then searched against GNPS spectral libraries. The library spectra were filtered in the same manner as the input data. All matches kept between network spectra and library spectra were required to have a score above 0.5 and at least 6 matched peaks. Analog search was enabled against the library with a maximum mass shift of 100 Da. Molecular networks were visualised using Cytoscape (version 3.4.0) and all solvent blanks (i.e. MeOH or hexane:MeOH mixture that was used extraction of algal material) were subtracted. These solvents were evaporated, dissolved in MeOH and injected to UPLC-MS/MS under the same conditions as the algal extract samples. An Euler diagram of MS/MS features derived from GNPS analysis was generated using the Venn and Euler Diagrams app^89^ in the Cytoscape 3.4 environment^90^.

### Desorption electrospray ionisation-imaging mass spectrometry (DESI-IMS)

#### Sample preparation

Since the surfaces of *F*. *vesiculosus* thalli were not flat and analysis by DESI-IMS requires completely flat surfaces, DESI-IMS study was performed on algal imprints (Fig. S10). For this purpose, freshly collected *F*. *vesiculosus* material (i.e. untreated) was placed between two microscopic slides (Superfrost Plus, Thermo Scientific, Massachusetts, USA) and pressed under weight for 4 days^91^. The algal material was then removed and imprints were stored at −20 °C until data acquisition. For chemical imaging of the algal cross sections, frozen *F*. *vesiculosus* material was embedded in 2% CMC (carboxymethyl cellulose, Carl Roth, Karlsruhe, Germany). Serial slices with a thickness of 60 µm were prepared using a CM3050 S cryostat (Leica, Wetzlar, Germany) and mounted onto Superfrost Plus microscope slides. The slides were stored at −20 °C until data acquisition.

#### Data acquisition

DESI-IMS was performed with a Xevo G2-XS QTof Mass Spectrometer (Waters, Milford, MA, USA) equipped with a DESI source (Prosolia, Indianapolis, USA) coupled with an external syringe pump. The mixture of MeOH:H_2_O (98:2, v/v) was used as desorption solvent at a flow rate of 2 µL/min. The optimised MS instrumental parameters in the positive ion mode used were as follows: 0.5 MPa nebuliser gas (N_2_) pressure, 100 °C capillary temperature and 5 kV spray voltage. The most intense 1000 ions were considered for both surface imprints and cross sections for the data processing step. DESI-IMS experiments were performed by continuously scanning the algal imprints and cross sections in the *x*-direction at a surface velocity of 160 µm/s for imprint and 35 µm/s for cross sections. The pixel size was set to 160 µm by 160 µm for imprints, and 35 µm by 35 µm for cross sections. Total run time was between 7 h and 8 h, respectively. All experiments were performed in positive mode and data were collected in full scan mode (*m/z* 50–1000). The DESI geometry parameters were optimised, resulting in a tip-to-surface distance of 2–3 mm, a tip-to-inlet distance of 4–6 mm, an inlet-to-surface distance of 1 mm, an incident angle of 75° and a capillary protrusion of 0.5 mm. Data acquisition and analysis were monitored using HDImaging software (version 4.1, Waters) and MassLynx software (version 4.1, Waters).

#### Statistical analysis

The similarity/dissimilarity between all samples for the untargeted metabolome analysis (UPLC-MS/MS) was displayed as a principal coordinates (PCoA) plot based on a Euclidian distance matrix and as Hierarchical Ascendant Classification (HAC). Statistical analyses were performed using XLSTAT software (version 2016, Addinsoft) and the 3D Plot visualization software, part of XLSTAT software.

#### ^1^H-NMR spectroscopy

The surface extract was loaded on a C18 SPE cartridge (Chromabond C18, Macherey-Nagel, Germany) and eluted with H_2_O (100%) and subsequently with 100% MeOH. They were evaporated to dryness by a rotary evaporator. ^1^H-NMR spectra of these extracts (10 mg each) as well as the pure standard compounds (i.e. alanine, proline, serine, threonine, citric acid, fucoxanthin, 5 mg each) were acquired in MeOD and D_2_O on a Bruker DRX 500 (500 MHz) spectrometer. The residual solvent signal of CD_3_OD-*d*4 and D_2_O were used as internal reference (δ_H_ 3.31 and δ_H_ 4.79). The NMR spectra were processed using MestReNova version 1.3 software.

## Supplementary information

Supplementary Information

## Data Availability

The datasets generated and analysed during the current study are available from the corresponding author on reasonable request.
