# Supplementary material for: Mapping the Surface Microbiome and Metabolome of Brown Seaweed Fucus vesiculosus by Amplicon Sequencing, Integrated Metabolomics and Imaging Techniques
Source: Sci Rep. 2019 Jan 31;9:1061. doi: 10.1038/s41598-018-37914-8 (PMC6355876; doi:10.1038/s41598-018-37914-8)
Supplement: Supplementary file 1 — Supplementary Information [file 41598_2018_37914_MOESM1_ESM.doc]

**Supplementary Information**

**Mapping the Surface Microbiome and Metabolome of Brown Seaweed *Fucus vesiculosus* by Amplicon Sequencing, Integrated Metabolomics and Imaging Techniques**

Delphine Parrot^1^, Martina Blümel^1^, Caroline Utermann^1^, Giuseppina Chianese^1^, Stefan Krause^2^, Alexander Kovalev^3^, Stanislav N. Gorb^3^, Deniz Tasdemir^1,4^,*

^1^GEOMAR Centre for Marine Biotechnology, Research Unit Marine Natural Products Chemistry, GEOMAR Helmholtz Centre for Ocean Research Kiel, Am Kiel-Kanal 44, Kiel 24106, Germany

^2^Research Unit Marine Geosystems, GEOMAR Helmholtz Centre for Ocean Research Kiel, Wischhofstrasse 1-3, Kiel 24148, Germany

^3^Department of Functional Morphology and Biomechanics, Institute of Zoology, Kiel University, Am Botanischen Garten 9, Kiel 24118, Germany

^4^Kiel University, Christian-Albrechts-Platz 4, Kiel 24118, Germany

*To whom correspondence should be addressed. Email: [dtasdemir@geomar.de](mailto:dtasdemir@geomar.de)

**Table S1.** Abundances of OTUs identified from *F. vesiculosus* and seawater reference samples. The data were compiled by amplicon sequencing of the V3-V4 hypervariable region of the 16S rRNA gene (primers: 341F_ill and 802R_ill). The taxonomic classification is given until the highest possible taxonomic rank. The abbreviation of whole seaweed samples is ‘Fv’, uncl: unclassified.

| **OTU** | **Taxonomic classification** | **Sample / Replicate** | | | | | | | | | | | |
| --- | --- | --- | --- | --- | --- | --- | --- | --- | --- | --- | --- | --- | --- |
|  |  | **Thallus** | | | **Tip** | | | **Whole seaweed (Fv)** | | | **Seawater** | | |
|  |  | **#1** | **#2** | **#3** | **#1** | **#2** | **#3** | **#1** | **#2** | **#3** | **#1** | **#2** | **#3** |
| 1 | uncl. Altererythrobacter | 643 | 654 | 594 | 356 | 214 | 114 | 619 | 397 | 399 | 13 | 35 | 31 |
| 2 | Octadecabacter ponticola | 72 | 91 | 181 | 450 | 315 | 699 | 224 | 224 | 431 | 28 | 39 | 31 |
| 3 | uncl. Planctomycetaceae | 55 | 116 | 22 | 622 | 413 | 81 | 313 | 397 | 64 | 5 | 4 | 3 |
| 4 | Planktomarina temperata | 0 | 0 | 1 | 0 | 0 | 0 | 0 | 0 | 1 | 489 | 666 | 726 |
| 5 | uncl. Rhodobacteraceae | 55 | 92 | 72 | 208 | 172 | 79 | 144 | 123 | 60 | 9 | 6 | 5 |
| 6 | uncl. Synechococcales | 158 | 120 | 56 | 241 | 54 | 72 | 268 | 209 | 140 | 0 | 0 | 2 |
| 7 | Robiginitomaculum antarcticum | 65 | 105 | 60 | 402 | 224 | 91 | 204 | 176 | 70 | 3 | 7 | 7 |
| 8 | uncl. Sulfitobacter | 173 | 173 | 125 | 63 | 51 | 87 | 138 | 112 | 102 | 11 | 27 | 20 |
| 9 | uncl. Hyphomonadaceae | 15 | 21 | 28 | 343 | 442 | 41 | 118 | 151 | 19 | 3 | 3 | 18 |
| 10 | Litorimonas cladophorae | 109 | 90 | 84 | 246 | 78 | 158 | 138 | 121 | 142 | 6 | 3 | 11 |
| 11 | Candidatus Pelagibacter ubique | 0 | 0 | 0 | 0 | 0 | 0 | 0 | 0 | 2 | 415 | 787 | 708 |
| 12 | uncl. Erythrobacteraceae | 65 | 259 | 119 | 76 | 237 | 19 | 193 | 138 | 63 | 4 | 4 | 3 |
| 13 | Boseongicola aestuarii | 109 | 98 | 132 | 17 | 14 | 36 | 29 | 32 | 93 | 5 | 8 | 17 |
| 14 | uncl. Cyanobacteria | 32 | 52 | 25 | 44 | 122 | 106 | 63 | 63 | 60 | 5 | 6 | 8 |
| 15 | uncl. Erythrobacter | 257 | 356 | 99 | 14 | 20 | 12 | 151 | 112 | 16 | 0 | 6 | 13 |
| 16 | uncl. Rhodobacteraceae | 40 | 44 | 62 | 9 | 11 | 71 | 58 | 52 | 68 | 8 | 16 | 18 |
| 17 | uncl. Loktanella | 28 | 31 | 64 | 34 | 45 | 83 | 45 | 28 | 163 | 17 | 29 | 33 |
| 18 | uncl. Synechococcales | 40 | 18 | 33 | 104 | 54 | 73 | 98 | 107 | 79 | 0 | 1 | 1 |
| 19 | Nisaea denitrificans | 0 | 0 | 0 | 0 | 0 | 0 | 0 | 0 | 0 | 70 | 127 | 128 |
| 20 | Litoreibacter meonggei | 42 | 50 | 26 | 28 | 21 | 33 | 29 | 36 | 36 | 15 | 31 | 17 |
| 21 | uncl. Rhodobacteraceae | 4 | 6 | 38 | 6 | 10 | 108 | 9 | 5 | 66 | 20 | 23 | 20 |
| 22 | uncl. Rhodobacteraceae | 65 | 47 | 42 | 7 | 3 | 31 | 27 | 23 | 39 | 5 | 6 | 9 |
| 23 | Trichocoleus desertorum | 98 | 85 | 23 | 7 | 3 | 8 | 38 | 65 | 11 | 0 | 0 | 3 |
| 24 | Foliisarcina bertiogensis | 103 | 62 | 7 | 3 | 4 | 4 | 50 | 38 | 8 | 1 | 1 | 3 |
| 25 | uncl. Acidimicrobiales | 17 | 21 | 68 | 6 | 5 | 40 | 12 | 21 | 75 | 0 | 0 | 2 |
| 26 | uncl. Phyllobacteriaceae | 31 | 14 | 47 | 3 | 16 | 58 | 16 | 13 | 44 | 14 | 25 | 15 |
| 27 | Geminicoccus roseus | 16 | 30 | 78 | 2 | 1 | 53 | 20 | 16 | 69 | 1 | 5 | 4 |
| 28 | uncl. Bacteria | 56 | 21 | 8 | 32 | 3 | 14 | 82 | 19 | 14 | 1 | 0 | 4 |
| 29 | uncl. Proteobacteria | 31 | 21 | 18 | 10 | 25 | 25 | 31 | 20 | 22 | 1 | 6 | 2 |
| 30 | uncl. Deinococcales | 30 | 24 | 32 | 2 | 3 | 32 | 7 | 5 | 31 | 1 | 1 | 2 |
| 31 | uncl. Cyanobacteria | 0 | 0 | 0 | 0 | 0 | 0 | 0 | 0 | 0 | 54 | 74 | 53 |
| 32 | uncl. Iamiaceae | 23 | 25 | 38 | 4 | 4 | 36 | 13 | 23 | 39 | 1 | 1 | 3 |
| 33 | uncl. Alphaproteobacteria | 9 | 4 | 7 | 68 | 55 | 8 | 27 | 30 | 4 | 0 | 3 | 1 |
| 34 | Algisphaera agarilytica | 50 | 158 | 47 | 2 | 5 | 4 | 32 | 48 | 9 | 2 | 5 | 2 |
| 35 | Candidatus Pelagibacter ubique | 0 | 0 | 0 | 0 | 0 | 0 | 0 | 0 | 0 | 82 | 133 | 154 |
| 36 | Sphingorhabdus litoris | 97 | 36 | 4 | 1 | 1 | 5 | 6 | 13 | 0 | 1 | 2 | 5 |
| 37 | uncl. Rhodobacteraceae | 16 | 24 | 19 | 3 | 1 | 18 | 11 | 5 | 13 | 1 | 3 | 1 |
| 38 | uncl. Microbacteriaceae | 0 | 0 | 0 | 0 | 0 | 0 | 0 | 0 | 0 | 23 | 65 | 55 |
| 39 | Ilumatobacter nonamiensis | 15 | 11 | 17 | 2 | 4 | 17 | 22 | 12 | 27 | 0 | 2 | 2 |
| 40 | uncl. Rhodospirillales | 0 | 0 | 0 | 0 | 0 | 1 | 0 | 0 | 0 | 15 | 23 | 29 |
| 41 | uncl. Filomicrobium | 27 | 42 | 51 | 2 | 2 | 17 | 20 | 20 | 48 | 0 | 2 | 4 |
| 42 | uncl. Alphaproteobacteria | 0 | 0 | 0 | 0 | 0 | 0 | 0 | 0 | 0 | 36 | 50 | 49 |
| 43 | uncl. Rhodobacteraceae | 4 | 17 | 17 | 1 | 9 | 6 | 6 | 4 | 11 | 3 | 3 | 5 |
| 44 | uncl. Bacteria | 4 | 3 | 5 | 13 | 37 | 9 | 18 | 6 | 5 | 3 | 7 | 3 |
| 45 | uncl. Bacteria | 2 | 8 | 10 | 17 | 15 | 33 | 12 | 9 | 6 | 0 | 1 | 0 |
| 46 | uncl. Cyanobacteria | 4 | 3 | 6 | 6 | 5 | 23 | 17 | 14 | 8 | 2 | 2 | 3 |
| 47 | uncl. Deltaproteobacteria | 17 | 23 | 13 | 2 | 0 | 13 | 22 | 8 | 7 | 0 | 1 | 0 |
| 48 | Algisphaera agarilytica | 23 | 29 | 62 | 0 | 0 | 41 | 14 | 13 | 28 | 2 | 2 | 0 |
| 49 | uncl. Bacteria | 1 | 5 | 0 | 28 | 4 | 0 | 41 | 22 | 1 | 0 | 0 | 1 |
| 50 | uncl. Deltaproteobacteria | 6 | 6 | 3 | 9 | 6 | 20 | 18 | 14 | 18 | 0 | 0 | 0 |
| 51 | uncl. Rubritalea | 1 | 1 | 1 | 24 | 15 | 0 | 14 | 8 | 1 | 0 | 0 | 0 |
| 52 | uncl. Rhizobiales | 2 | 0 | 2 | 5 | 67 | 2 | 3 | 7 | 1 | 0 | 0 | 2 |
| 53 | uncl. Hyphomonas | 7 | 19 | 23 | 9 | 18 | 16 | 14 | 11 | 37 | 15 | 24 | 6 |
| 54 | uncl. Planctomycetaceae | 8 | 9 | 0 | 20 | 10 | 6 | 18 | 16 | 10 | 3 | 5 | 0 |
| 55 | uncl. Deltaproteobacteria | 19 | 14 | 18 | 0 | 0 | 10 | 14 | 6 | 4 | 1 | 0 | 1 |
| 56 | uncl. Deltaproteobacteria | 4 | 6 | 18 | 2 | 1 | 27 | 8 | 5 | 14 | 1 | 1 | 1 |
| 57 | uncl. Rhizobiales | 10 | 7 | 5 | 2 | 1 | 13 | 12 | 12 | 13 | 0 | 2 | 3 |
| 58 | uncl. Rhodobacteraceae | 1 | 5 | 5 | 1 | 1 | 1 | 2 | 3 | 1 | 10 | 21 | 13 |
| 59 | uncl. Alphaproteobacteria | 0 | 0 | 0 | 0 | 0 | 0 | 0 | 0 | 0 | 21 | 32 | 45 |
| 60 | uncl. Alphaproteobacteria | 2 | 1 | 18 | 1 | 4 | 31 | 0 | 5 | 25 | 2 | 4 | 3 |
| 61 | uncl. Rhodobacteraceae | 7 | 4 | 5 | 2 | 4 | 3 | 5 | 1 | 2 | 7 | 15 | 5 |
| 62 | uncl. Rhodobacteraceae | 0 | 0 | 0 | 0 | 0 | 0 | 0 | 0 | 0 | 6 | 25 | 31 |
| 63 | Candidatus Pelagibacter ubique | 0 | 0 | 0 | 0 | 0 | 0 | 0 | 0 | 0 | 39 | 57 | 65 |
| 64 | uncl. Proteobacteria | 2 | 0 | 2 | 2 | 6 | 17 | 5 | 0 | 14 | 3 | 12 | 3 |
| 65 | uncl. Bacteria | 30 | 12 | 3 | 1 | 1 | 1 | 12 | 4 | 3 | 1 | 0 | 1 |
| 66 | uncl. Alphaproteobacteria | 5 | 12 | 1 | 0 | 25 | 9 | 11 | 8 | 4 | 3 | 0 | 0 |
| 67 | uncl. Luteolibacter | 2 | 2 | 1 | 6 | 13 | 6 | 5 | 2 | 5 | 0 | 1 | 1 |
| 68 | uncl. Alphaproteobacteria | 1 | 1 | 2 | 29 | 23 | 0 | 11 | 2 | 0 | 1 | 1 | 1 |
| 69 | uncl. Rhodospirillaceae | 0 | 0 | 0 | 0 | 0 | 0 | 0 | 0 | 0 | 9 | 14 | 13 |
| 70 | uncl. Alphaproteobacteria | 6 | 2 | 1 | 26 | 6 | 0 | 25 | 2 | 0 | 0 | 0 | 1 |
| 71 | uncl. Alphaproteobacteria | 3 | 1 | 17 | 1 | 0 | 13 | 5 | 1 | 26 | 1 | 0 | 1 |
| 72 | uncl. Alphaproteobacteria | 0 | 0 | 0 | 11 | 41 | 3 | 9 | 2 | 1 | 0 | 0 | 0 |
| 73 | Arcobacter cryaerophilus | 0 | 0 | 0 | 0 | 0 | 0 | 0 | 0 | 0 | 7 | 5 | 11 |
| 74 | uncl. Saprospirales | 2 | 4 | 6 | 0 | 1 | 17 | 1 | 1 | 15 | 0 | 0 | 0 |
| 75 | uncl. Hyphomicrobium | 4 | 12 | 16 | 9 | 2 | 16 | 12 | 9 | 32 | 0 | 2 | 4 |
| 76 | uncl. Actinobacteria | 6 | 7 | 2 | 2 | 1 | 6 | 4 | 2 | 8 | 0 | 0 | 1 |
| 77 | uncl. Alphaproteobacteria | 2 | 0 | 9 | 1 | 3 | 7 | 2 | 0 | 9 | 4 | 18 | 9 |
| 78 | uncl. Erythrobacter | 32 | 12 | 4 | 6 | 4 | 2 | 7 | 11 | 0 | 3 | 4 | 4 |
| 79 | uncl. Bacteria | 2 | 1 | 17 | 0 | 0 | 16 | 2 | 0 | 16 | 0 | 0 | 2 |
| 80 | uncl. Bacteria | 23 | 5 | 5 | 6 | 4 | 1 | 4 | 2 | 2 | 0 | 1 | 2 |
| 81 | uncl. Hyphomonas | 11 | 7 | 53 | 4 | 4 | 6 | 2 | 4 | 4 | 2 | 7 | 7 |
| 82 | Fretibacter rubidus | 15 | 7 | 14 | 3 | 0 | 2 | 0 | 2 | 1 | 4 | 8 | 8 |
| 83 | uncl. Cyanobacteria | 0 | 0 | 0 | 0 | 0 | 0 | 0 | 0 | 0 | 11 | 20 | 13 |
| 84 | Ahrensia kielensis | 3 | 0 | 7 | 0 | 4 | 8 | 2 | 1 | 11 | 1 | 3 | 1 |
| 85 | uncl. Arcobacter | 0 | 0 | 0 | 0 | 0 | 1 | 0 | 0 | 0 | 4 | 8 | 8 |
| 86 | uncl. Bacteria | 4 | 2 | 2 | 0 | 0 | 18 | 0 | 0 | 25 | 0 | 0 | 0 |
| 87 | uncl. Flavobacteriaceae | 0 | 0 | 0 | 0 | 0 | 0 | 0 | 0 | 0 | 6 | 12 | 15 |
| 88 | uncl. Bacteria | 1 | 0 | 15 | 4 | 4 | 16 | 2 | 1 | 1 | 2 | 2 | 2 |
| 89 | Algisphaera agarilytica | 5 | 22 | 42 | 0 | 0 | 8 | 5 | 2 | 7 | 0 | 2 | 2 |
| 90 | uncl. Alphaproteobacteria | 2 | 7 | 1 | 1 | 19 | 3 | 13 | 7 | 0 | 0 | 1 | 0 |
| 91 | uncl. Planctomycetaceae | 3 | 9 | 15 | 1 | 1 | 4 | 3 | 3 | 3 | 1 | 4 | 6 |
| 92 | Parvularcula flava | 2 | 10 | 39 | 4 | 7 | 19 | 2 | 2 | 11 | 0 | 0 | 0 |
| 93 | uncl. Proteobacteria | 1 | 1 | 1 | 13 | 11 | 0 | 5 | 4 | 4 | 0 | 1 | 0 |
| 94 | Bythopirellula goksoyri | 9 | 5 | 3 | 3 | 3 | 1 | 6 | 15 | 3 | 0 | 2 | 1 |
| 95 | Synechococcus rubescens | 0 | 0 | 0 | 1 | 0 | 0 | 0 | 0 | 0 | 9 | 21 | 17 |
| 96 | Litorimonas taeanensis | 2 | 0 | 28 | 1 | 0 | 9 | 1 | 0 | 3 | 0 | 3 | 6 |
| 97 | uncl. Bacteria | 13 | 6 | 3 | 0 | 0 | 3 | 7 | 2 | 5 | 1 | 2 | 2 |
| 98 | uncl. Planctomycetaceae | 2 | 2 | 6 | 5 | 0 | 11 | 5 | 10 | 6 | 1 | 1 | 0 |
| 99 | Geminicoccus roseus | 3 | 2 | 3 | 0 | 1 | 12 | 3 | 2 | 3 | 2 | 12 | 6 |
| 100 | uncl. Proteobacteria | 8 | 4 | 5 | 5 | 1 | 2 | 4 | 2 | 6 | 0 | 2 | 1 |
| 101 | uncl. Alphaproteobacteria | 0 | 0 | 0 | 0 | 0 | 0 | 0 | 0 | 0 | 7 | 18 | 23 |
| 102 | uncl. Alphaproteobacteria | 0 | 0 | 6 | 10 | 16 | 7 | 5 | 4 | 0 | 0 | 0 | 0 |
| 103 | uncl. Flavobacteriaceae | 9 | 5 | 6 | 0 | 0 | 1 | 3 | 0 | 3 | 0 | 0 | 0 |
| 104 | uncl. Flavobacteriales | 0 | 0 | 0 | 0 | 0 | 0 | 0 | 0 | 0 | 9 | 24 | 19 |
| 105 | uncl. Fusobacteriaceae | 0 | 0 | 0 | 0 | 0 | 0 | 0 | 0 | 0 | 3 | 4 | 6 |
| 106 | uncl. Rhodobacteraceae | 7 | 4 | 1 | 1 | 0 | 4 | 5 | 5 | 3 | 0 | 0 | 0 |
| 107 | uncl. Bacteria | 4 | 2 | 2 | 11 | 4 | 2 | 6 | 2 | 5 | 0 | 1 | 1 |
| 108 | uncl. Maribacter | 4 | 3 | 13 | 0 | 0 | 2 | 2 | 1 | 10 | 0 | 0 | 2 |
| 109 | uncl. Bacteria | 1 | 0 | 3 | 0 | 0 | 26 | 2 | 2 | 2 | 0 | 2 | 3 |
| 110 | uncl. Desulfobacteraceae | 2 | 0 | 2 | 2 | 0 | 4 | 7 | 3 | 3 | 0 | 0 | 0 |
| 111 | uncl. Rhodospirillaceae | 0 | 0 | 0 | 0 | 0 | 0 | 0 | 0 | 0 | 5 | 6 | 10 |
| 112 | uncl. Alphaproteobacteria | 2 | 2 | 9 | 10 | 1 | 4 | 3 | 0 | 1 | 3 | 4 | 2 |
| 113 | uncl. Arcobacter | 0 | 0 | 0 | 0 | 0 | 0 | 0 | 0 | 0 | 4 | 5 | 6 |
| 114 | uncl. Alphaproteobacteria | 0 | 0 | 5 | 0 | 0 | 31 | 0 | 0 | 2 | 1 | 2 | 0 |
| 115 | uncl. Arcobacter | 0 | 1 | 0 | 0 | 0 | 0 | 0 | 1 | 0 | 4 | 4 | 4 |
| 116 | uncl. Bacteria | 3 | 0 | 9 | 0 | 0 | 3 | 0 | 1 | 10 | 2 | 1 | 6 |
| 117 | uncl. Rhodobacteraceae | 0 | 0 | 0 | 0 | 0 | 4 | 0 | 0 | 0 | 4 | 13 | 4 |
| 118 | Robiginitomaculum antarcticum | 6 | 8 | 12 | 0 | 1 | 5 | 2 | 0 | 3 | 2 | 0 | 0 |
| 119 | uncl. Erythrobacteraceae | 0 | 5 | 13 | 1 | 0 | 1 | 4 | 1 | 14 | 0 | 5 | 4 |
| 120 | uncl. Actinobacteria | 4 | 2 | 13 | 1 | 0 | 12 | 0 | 0 | 14 | 0 | 0 | 0 |
| 121 | Litorilinea aerophila | 3 | 3 | 0 | 0 | 1 | 7 | 0 | 1 | 1 | 5 | 11 | 3 |
| 122 | uncl. Bacteria | 0 | 0 | 0 | 1 | 26 | 0 | 4 | 1 | 0 | 0 | 1 | 0 |
| 123 | uncl. Rhodobacteraceae | 1 | 0 | 2 | 0 | 0 | 0 | 1 | 1 | 0 | 4 | 8 | 10 |
| 124 | uncl. Cyanobacteria | 0 | 0 | 0 | 0 | 0 | 0 | 0 | 0 | 0 | 2 | 15 | 9 |
| 125 | uncl. Rhodospirillaceae | 0 | 0 | 0 | 0 | 0 | 0 | 0 | 0 | 0 | 5 | 6 | 7 |
| 126 | uncl. Bacteria | 0 | 1 | 21 | 0 | 0 | 9 | 0 | 0 | 0 | 0 | 0 | 1 |
| 127 | uncl. Rhodobacteraceae | 2 | 1 | 0 | 5 | 2 | 1 | 3 | 5 | 1 | 0 | 1 | 3 |
| 128 | uncl. Rhodobacteraceae | 0 | 0 | 2 | 0 | 1 | 4 | 0 | 0 | 1 | 3 | 11 | 3 |
| 129 | uncl. Flavobacteriaceae | 1 | 2 | 3 | 1 | 3 | 4 | 2 | 1 | 3 | 0 | 0 | 1 |
| 130 | uncl. Nannocystaceae | 6 | 8 | 0 | 0 | 0 | 0 | 0 | 4 | 1 | 0 | 1 | 1 |
| 131 | uncl. Proteobacteria | 4 | 1 | 5 | 0 | 0 | 2 | 0 | 5 | 8 | 0 | 2 | 2 |
| 132 | uncl. Nannocystaceae | 0 | 0 | 13 | 0 | 0 | 1 | 6 | 0 | 0 | 0 | 0 | 0 |
| 133 | uncl. Sulfurovum | 0 | 0 | 0 | 0 | 0 | 0 | 0 | 0 | 0 | 6 | 7 | 8 |
| 134 | uncl. Hyphomonadaceae | 0 | 10 | 2 | 0 | 2 | 0 | 6 | 13 | 0 | 0 | 1 | 0 |
| 135 | uncl. Phyllobacteriaceae | 3 | 2 | 0 | 1 | 3 | 5 | 0 | 2 | 1 | 2 | 9 | 5 |
| 136 | uncl. Alphaproteobacteria | 0 | 0 | 3 | 0 | 0 | 16 | 3 | 1 | 10 | 1 | 0 | 0 |
| 137 | uncl. Alphaproteobacteria | 1 | 0 | 0 | 5 | 20 | 0 | 6 | 0 | 0 | 0 | 0 | 1 |
| 138 | Litorimonas taeanensis | 4 | 3 | 8 | 0 | 0 | 5 | 2 | 0 | 8 | 0 | 3 | 1 |
| 139 | uncl. Maribacter | 0 | 1 | 8 | 1 | 0 | 4 | 1 | 1 | 9 | 0 | 0 | 1 |
| 140 | uncl. Rhizobiales | 4 | 1 | 4 | 0 | 0 | 6 | 3 | 0 | 5 | 1 | 1 | 1 |
| 141 | uncl. Bacteria | 0 | 2 | 18 | 0 | 3 | 0 | 1 | 0 | 0 | 0 | 1 | 2 |
| 142 | Pacificibacter marinus | 0 | 1 | 1 | 0 | 1 | 0 | 0 | 0 | 0 | 4 | 7 | 7 |
| 143 | uncl. Rubritalea | 1 | 1 | 2 | 2 | 1 | 5 | 2 | 0 | 3 | 0 | 1 | 1 |
| 144 | uncl. Bacteria | 0 | 1 | 3 | 10 | 4 | 3 | 1 | 0 | 2 | 0 | 3 | 1 |
| 145 | uncl. Sphingomonadaceae | 0 | 0 | 0 | 8 | 12 | 0 | 2 | 3 | 0 | 0 | 0 | 0 |
| 146 | uncl. Actinobacteria | 10 | 0 | 4 | 2 | 4 | 3 | 5 | 6 | 1 | 0 | 0 | 0 |
| 147 | Sulfurimonas autotrophica | 0 | 0 | 0 | 0 | 0 | 0 | 0 | 0 | 0 | 3 | 4 | 5 |
| 148 | Nonlabens ulvanivorans | 1 | 0 | 3 | 2 | 3 | 2 | 4 | 2 | 1 | 0 | 1 | 0 |
| 149 | uncl. Flavobacteriaceae | 3 | 4 | 1 | 0 | 1 | 0 | 3 | 2 | 1 | 0 | 0 | 1 |
| 150 | uncl. Bacteria | 5 | 2 | 5 | 0 | 0 | 6 | 1 | 1 | 4 | 1 | 0 | 0 |
| 151 | Synechococcus rubescens | 0 | 0 | 0 | 0 | 0 | 0 | 0 | 0 | 0 | 7 | 9 | 11 |
| 152 | Polaribacter dokdonensis | 0 | 0 | 1 | 0 | 1 | 5 | 0 | 0 | 4 | 1 | 3 | 1 |
| 153 | uncl. Alphaproteobacteria | 1 | 0 | 3 | 0 | 1 | 16 | 3 | 1 | 2 | 0 | 0 | 0 |
| 154 | uncl. Alphaproteobacteria | 1 | 0 | 3 | 0 | 0 | 11 | 0 | 1 | 9 | 0 | 0 | 2 |
| 155 | uncl. Rhizobiales | 0 | 0 | 0 | 5 | 4 | 0 | 9 | 2 | 2 | 0 | 0 | 0 |
| 156 | Dokdonia eikasta | 0 | 2 | 0 | 1 | 4 | 0 | 5 | 3 | 1 | 0 | 0 | 0 |
| 157 | uncl. Synechococcales | 4 | 3 | 2 | 4 | 1 | 2 | 5 | 3 | 2 | 0 | 0 | 0 |
| 158 | uncl. Hyphomonadaceae | 5 | 2 | 6 | 0 | 1 | 1 | 1 | 1 | 2 | 2 | 1 | 4 |
| 159 | uncl. Bacteria | 10 | 3 | 8 | 1 | 1 | 0 | 0 | 0 | 0 | 0 | 1 | 0 |
| 160 | uncl. Rhodobacteraceae | 0 | 0 | 0 | 0 | 0 | 0 | 1 | 0 | 1 | 6 | 5 | 5 |
| 161 | uncl. Haliscomenobacteraceae | 1 | 3 | 2 | 4 | 4 | 1 | 4 | 3 | 1 | 0 | 0 | 0 |
| 162 | uncl. Blastocatellia | 30 | 12 | 0 | 0 | 0 | 0 | 0 | 0 | 0 | 0 | 0 | 2 |
| 163 | uncl. Rhizobiales | 0 | 0 | 1 | 0 | 1 | 1 | 0 | 0 | 2 | 3 | 10 | 3 |
| 164 | Portibacter lacus | 2 | 0 | 2 | 3 | 3 | 4 | 4 | 4 | 1 | 0 | 0 | 0 |
| 165 | uncl. Flavobacteriaceae | 1 | 0 | 1 | 1 | 1 | 5 | 2 | 2 | 3 | 0 | 0 | 0 |
| 166 | uncl. Alphaproteobacteria | 0 | 0 | 2 | 0 | 5 | 5 | 1 | 1 | 6 | 2 | 1 | 1 |
| 167 | uncl. Proteobacteria | 3 | 1 | 2 | 6 | 2 | 0 | 3 | 0 | 0 | 0 | 0 | 2 |
| 168 | uncl. Cyanobacteria | 0 | 0 | 0 | 0 | 0 | 0 | 0 | 0 | 0 | 2 | 9 | 7 |
| 169 | uncl. Rhodobacteraceae | 1 | 3 | 1 | 1 | 1 | 4 | 1 | 2 | 1 | 0 | 0 | 1 |
| 170 | uncl. Deltaproteobacteria | 3 | 0 | 10 | 0 | 0 | 2 | 3 | 1 | 1 | 0 | 0 | 0 |
| 171 | uncl. Bacteria | 11 | 8 | 0 | 0 | 0 | 0 | 0 | 0 | 0 | 0 | 0 | 0 |
| 172 | uncl. Bacteria | 3 | 3 | 0 | 0 | 0 | 0 | 9 | 4 | 1 | 0 | 0 | 0 |
| 173 | uncl. Microbacteriaceae | 0 | 0 | 0 | 0 | 0 | 0 | 0 | 0 | 0 | 2 | 9 | 9 |
| 174 | uncl. Rhodobacteraceae | 0 | 0 | 1 | 1 | 0 | 0 | 1 | 0 | 1 | 4 | 3 | 3 |
| 175 | uncl. Bacteria | 3 | 0 | 3 | 1 | 0 | 0 | 4 | 0 | 6 | 1 | 1 | 1 |
| 176 | uncl. Bacteria | 3 | 0 | 3 | 9 | 0 | 0 | 3 | 0 | 1 | 0 | 0 | 1 |
| 177 | uncl. Alphaproteobacteria | 1 | 3 | 0 | 3 | 3 | 2 | 5 | 4 | 0 | 0 | 0 | 0 |
| 178 | uncl. Bacteria | 0 | 0 | 13 | 0 | 0 | 1 | 0 | 0 | 2 | 0 | 0 | 3 |
| 179 | Vampirovibrio chlorellavorus | 6 | 7 | 0 | 0 | 0 | 0 | 1 | 4 | 0 | 0 | 0 | 0 |
| 180 | Kiloniella laminariae | 3 | 0 | 1 | 1 | 0 | 2 | 0 | 0 | 5 | 2 | 7 | 0 |
| 181 | uncl. Proteobacteria | 0 | 0 | 0 | 6 | 0 | 3 | 2 | 0 | 1 | 0 | 2 | 3 |
| 182 | uncl. Rhodobacteraceae | 0 | 3 | 1 | 0 | 8 | 1 | 0 | 0 | 1 | 0 | 0 | 1 |
| 183 | uncl. Cyanobacteria | 0 | 0 | 0 | 0 | 2 | 3 | 0 | 0 | 0 | 2 | 2 | 6 |
| 184 | uncl. Nostocales | 0 | 1 | 1 | 0 | 0 | 2 | 1 | 0 | 2 | 0 | 0 | 1 |
| 185 | Rubritalea marina | 1 | 1 | 0 | 1 | 2 | 3 | 2 | 0 | 1 | 1 | 0 | 0 |
| 186 | uncl. Bacteria | 5 | 1 | 2 | 0 | 0 | 2 | 0 | 0 | 1 | 2 | 2 | 4 |
| 187 | uncl. Bacteria | 2 | 0 | 10 | 0 | 0 | 4 | 0 | 0 | 2 | 0 | 0 | 0 |
| 188 | uncl. Phyllobacteriaceae | 0 | 1 | 1 | 0 | 0 | 8 | 1 | 0 | 3 | 3 | 3 | 0 |
| 189 | uncl. Pseudahrensia | 2 | 4 | 2 | 0 | 0 | 4 | 2 | 0 | 4 | 1 | 0 | 0 |
| 190 | Emcibacter nanhaiensis | 0 | 0 | 0 | 0 | 0 | 0 | 0 | 0 | 0 | 3 | 9 | 7 |
| 191 | Roseobacter litoralis | 0 | 0 | 2 | 0 | 0 | 0 | 0 | 0 | 0 | 7 | 22 | 4 |
| 192 | uncl. Rickettsia | 0 | 0 | 0 | 0 | 0 | 0 | 0 | 0 | 0 | 7 | 13 | 13 |
| 193 | Lewinella persica | 1 | 1 | 3 | 1 | 0 | 3 | 1 | 1 | 3 | 0 | 0 | 0 |
| 194 | uncl. Rhodobacteraceae | 1 | 1 | 2 | 0 | 1 | 2 | 1 | 1 | 3 | 0 | 0 | 0 |
| 195 | uncl. Flavobacterium | 1 | 0 | 0 | 0 | 0 | 2 | 1 | 0 | 1 | 0 | 0 | 0 |
| 196 | uncl. Flavobacteriaceae | 0 | 0 | 2 | 0 | 0 | 1 | 1 | 0 | 1 | 1 | 3 | 1 |
| 197 | uncl. Bacteria | 2 | 2 | 3 | 0 | 0 | 2 | 1 | 4 | 1 | 0 | 0 | 1 |
| 198 | uncl. Proteobacteria | 1 | 0 | 0 | 6 | 2 | 1 | 0 | 2 | 2 | 0 | 0 | 0 |
| 199 | uncl. Bacteria | 5 | 3 | 1 | 0 | 0 | 0 | 1 | 2 | 0 | 1 | 2 | 1 |
| 200 | uncl. Bacteria | 4 | 0 | 9 | 0 | 0 | 1 | 0 | 0 | 0 | 1 | 0 | 0 |
| 201 | uncl. Bacteria | 7 | 3 | 2 | 0 | 0 | 0 | 1 | 0 | 0 | 0 | 0 | 2 |
| 202 | Luteolibacter algae | 1 | 0 | 0 | 1 | 4 | 0 | 4 | 1 | 0 | 0 | 0 | 0 |
| 203 | Lewinella nigricans | 5 | 2 | 2 | 0 | 0 | 2 | 0 | 0 | 0 | 0 | 0 | 1 |
| 204 | uncl. Proteobacteria | 0 | 1 | 6 | 0 | 0 | 3 | 1 | 0 | 0 | 1 | 1 | 0 |
| 205 | uncl. Saprospirales | 1 | 1 | 1 | 2 | 1 | 2 | 1 | 1 | 1 | 0 | 0 | 0 |
| 206 | Portibacter lacus | 0 | 2 | 3 | 0 | 0 | 3 | 4 | 1 | 1 | 0 | 0 | 0 |
| 207 | uncl. Proteobacteria | 3 | 3 | 2 | 0 | 0 | 0 | 0 | 2 | 2 | 0 | 0 | 0 |
| 208 | Synechococcus rubescens | 0 | 0 | 0 | 0 | 0 | 0 | 0 | 0 | 0 | 4 | 8 | 3 |
| 209 | uncl. Proteobacteria | 1 | 0 | 2 | 0 | 0 | 4 | 0 | 2 | 0 | 1 | 2 | 1 |
| 210 | uncl. Granulosicoccus | 1 | 2 | 2 | 1 | 1 | 0 | 0 | 2 | 1 | 0 | 0 | 0 |
| 211 | uncl. Bacteria | 0 | 1 | 2 | 1 | 0 | 5 | 3 | 0 | 0 | 1 | 0 | 2 |
| 212 | uncl. Cyanobacteria | 0 | 0 | 0 | 0 | 0 | 0 | 0 | 0 | 0 | 3 | 5 | 3 |
| 213 | uncl. Actinobacteria | 4 | 2 | 0 | 2 | 1 | 0 | 2 | 5 | 0 | 1 | 0 | 0 |
| 214 | uncl. Bacteria | 10 | 3 | 0 | 0 | 0 | 0 | 1 | 0 | 0 | 0 | 0 | 0 |
| 215 | uncl. Saprospirales | 0 | 1 | 2 | 1 | 0 | 1 | 1 | 2 | 1 | 0 | 0 | 0 |
| 216 | uncl. Alphaproteobacteria | 0 | 0 | 2 | 0 | 1 | 0 | 0 | 0 | 1 | 2 | 6 | 3 |
| 217 | uncl. Rhodobacteraceae | 0 | 0 | 0 | 0 | 1 | 7 | 0 | 1 | 1 | 0 | 0 | 1 |
| 218 | uncl. Alphaproteobacteria | 0 | 0 | 1 | 3 | 0 | 7 | 3 | 1 | 1 | 0 | 0 | 0 |
| 219 | uncl. Deltaproteobacteria | 0 | 0 | 0 | 6 | 3 | 0 | 2 | 2 | 0 | 0 | 0 | 0 |
| 220 | uncl. Planctomycetaceae | 2 | 0 | 6 | 0 | 0 | 2 | 0 | 0 | 1 | 2 | 0 | 1 |
| 221 | uncl. Bacteria | 0 | 0 | 0 | 0 | 0 | 0 | 0 | 0 | 0 | 2 | 4 | 7 |
| 222 | uncl. Bacteria | 2 | 2 | 3 | 1 | 0 | 3 | 0 | 1 | 1 | 0 | 0 | 1 |
| 223 | uncl. Proteobacteria | 0 | 0 | 1 | 1 | 1 | 3 | 2 | 2 | 2 | 0 | 1 | 0 |
| 224 | uncl. Proteobacteria | 7 | 0 | 0 | 0 | 0 | 0 | 1 | 2 | 1 | 0 | 0 | 1 |
| 225 | uncl. Deltaproteobacteria | 3 | 2 | 1 | 0 | 0 | 1 | 3 | 2 | 0 | 0 | 0 | 0 |
| 226 | uncl. Alphaproteobacteria | 5 | 6 | 0 | 0 | 0 | 0 | 1 | 1 | 0 | 0 | 1 | 0 |
| 227 | uncl. Bacteria | 0 | 0 | 0 | 0 | 0 | 0 | 0 | 0 | 0 | 4 | 5 | 4 |
| 228 | uncl. Alphaproteobacteria | 0 | 0 | 0 | 0 | 0 | 0 | 10 | 3 | 0 | 1 | 0 | 0 |
| 229 | uncl. Gammaproteobacteria | 0 | 0 | 1 | 0 | 0 | 0 | 0 | 0 | 0 | 2 | 4 | 1 |
| 230 | uncl. Alphaproteobacteria | 0 | 0 | 0 | 1 | 4 | 0 | 2 | 4 | 0 | 2 | 0 | 0 |
| 231 | Litorilinea aerophila | 1 | 0 | 1 | 0 | 0 | 6 | 1 | 1 | 0 | 1 | 1 | 1 |
| 232 | Hirschia baltica | 1 | 0 | 1 | 0 | 0 | 1 | 0 | 0 | 2 | 0 | 6 | 1 |
| 233 | uncl. Rhodobacteraceae | 1 | 1 | 1 | 0 | 1 | 1 | 1 | 0 | 2 | 0 | 1 | 0 |
| 234 | uncl. Desulfobacteraceae | 3 | 4 | 0 | 0 | 0 | 0 | 0 | 0 | 0 | 0 | 0 | 0 |
| 235 | uncl. Deltaproteobacteria | 0 | 0 | 0 | 0 | 0 | 2 | 0 | 4 | 5 | 0 | 0 | 0 |
| 236 | uncl. Proteobacteria | 7 | 1 | 0 | 0 | 1 | 0 | 0 | 0 | 0 | 1 | 0 | 0 |
| 237 | uncl. Roseivirga | 0 | 0 | 0 | 1 | 0 | 2 | 0 | 0 | 0 | 0 | 1 | 1 |
| 238 | uncl. Bacteria | 0 | 0 | 1 | 3 | 3 | 0 | 2 | 1 | 0 | 0 | 1 | 2 |
| 239 | uncl. Bacteria | 1 | 0 | 0 | 0 | 0 | 3 | 0 | 1 | 1 | 3 | 2 | 2 |
| 240 | uncl. Alphaproteobacteria | 0 | 0 | 0 | 0 | 0 | 0 | 0 | 0 | 0 | 5 | 3 | 5 |
| 241 | uncl. Rhizobiales | 2 | 2 | 1 | 0 | 1 | 2 | 2 | 2 | 0 | 0 | 0 | 0 |
| 242 | uncl. Rhizobiales | 0 | 1 | 0 | 0 | 0 | 7 | 1 | 0 | 2 | 0 | 0 | 1 |
| 243 | uncl. Bacteria | 0 | 0 | 3 | 0 | 0 | 1 | 0 | 0 | 1 | 3 | 2 | 2 |
| 244 | uncl. Rhodobacteraceae | 0 | 0 | 2 | 0 | 0 | 0 | 0 | 0 | 1 | 1 | 3 | 0 |
| 245 | uncl. Alphaproteobacteria | 0 | 0 | 0 | 0 | 0 | 0 | 0 | 0 | 0 | 4 | 4 | 4 |
| 246 | uncl. Proteobacteria | 0 | 1 | 2 | 0 | 1 | 0 | 1 | 2 | 0 | 0 | 1 | 2 |
| 247 | uncl. Bacteria | 6 | 3 | 1 | 0 | 0 | 0 | 1 | 0 | 0 | 0 | 0 | 0 |
| 248 | uncl. Proteobacteria | 0 | 0 | 0 | 1 | 0 | 1 | 0 | 0 | 8 | 0 | 0 | 0 |
| 249 | uncl. Cyanobacteria | 0 | 0 | 0 | 0 | 0 | 0 | 0 | 0 | 0 | 2 | 3 | 4 |
| 250 | Truepera radiovictrix | 1 | 2 | 3 | 0 | 0 | 0 | 2 | 0 | 1 | 1 | 1 | 1 |
| 251 | uncl. Bacteria | 0 | 0 | 1 | 0 | 0 | 0 | 0 | 0 | 0 | 1 | 4 | 4 |
| 252 | Magnetococcus marinus | 1 | 0 | 2 | 0 | 0 | 3 | 0 | 0 | 0 | 0 | 0 | 0 |
| 253 | uncl. Alphaproteobacteria | 0 | 0 | 0 | 3 | 1 | 0 | 2 | 2 | 0 | 1 | 2 | 0 |
| 254 | uncl. Bacteria | 0 | 0 | 1 | 0 | 0 | 4 | 1 | 0 | 5 | 0 | 0 | 0 |
| 255 | uncl. Bacteria | 1 | 1 | 3 | 0 | 0 | 3 | 0 | 0 | 2 | 0 | 1 | 0 |
| 256 | uncl. Bacteria | 0 | 0 | 1 | 0 | 0 | 2 | 0 | 0 | 0 | 0 | 6 | 1 |
| 257 | uncl. Alphaproteobacteria | 1 | 1 | 9 | 0 | 0 | 0 | 0 | 0 | 0 | 0 | 0 | 0 |
| 258 | uncl. Pseudorhodobacter | 0 | 0 | 0 | 0 | 0 | 0 | 0 | 0 | 0 | 1 | 3 | 3 |
| 259 | uncl. Actinobacteria | 5 | 0 | 1 | 0 | 0 | 0 | 0 | 0 | 0 | 0 | 0 | 1 |
| 260 | uncl. Bacteria | 1 | 3 | 1 | 1 | 0 | 2 | 1 | 0 | 0 | 0 | 1 | 1 |
| 261 | uncl. Haliscomenobacteraceae | 2 | 3 | 0 | 1 | 1 | 1 | 2 | 0 | 0 | 0 | 0 | 1 |
| 262 | uncl. Saprospirales | 1 | 0 | 1 | 2 | 1 | 0 | 1 | 1 | 0 | 0 | 0 | 0 |
| 263 | uncl. Lewinella | 2 | 1 | 1 | 0 | 0 | 2 | 1 | 0 | 0 | 0 | 0 | 0 |
| 264 | uncl. Hyphomonadaceae | 0 | 0 | 0 | 0 | 0 | 3 | 0 | 0 | 0 | 2 | 4 | 1 |
| 265 | uncl. Bacteria | 0 | 0 | 4 | 0 | 0 | 0 | 2 | 2 | 1 | 0 | 0 | 0 |
| 266 | uncl. Desulfobacteraceae | 0 | 0 | 0 | 0 | 5 | 0 | 0 | 0 | 0 | 0 | 0 | 0 |
| 267 | uncl. Kiloniella | 0 | 0 | 0 | 0 | 1 | 2 | 0 | 0 | 0 | 2 | 3 | 2 |
| 268 | uncl. Haliscomenobacteraceae | 3 | 3 | 1 | 0 | 0 | 0 | 0 | 1 | 0 | 0 | 1 | 0 |
| 269 | uncl. Saprospirales | 1 | 1 | 0 | 1 | 1 | 1 | 0 | 1 | 0 | 0 | 0 | 0 |
| 270 | uncl. Saprospirales | 0 | 0 | 0 | 0 | 0 | 2 | 0 | 0 | 3 | 0 | 1 | 1 |
| 271 | uncl. Proteobacteria | 1 | 0 | 0 | 0 | 1 | 3 | 1 | 1 | 2 | 0 | 0 | 0 |
| 272 | uncl. Lewinella | 1 | 1 | 1 | 0 | 0 | 1 | 2 | 0 | 1 | 0 | 0 | 0 |
| 273 | uncl. Saprospirales | 0 | 1 | 0 | 1 | 0 | 5 | 0 | 0 | 1 | 0 | 0 | 0 |
| 274 | uncl. Proteobacteria | 2 | 0 | 0 | 3 | 0 | 0 | 0 | 2 | 0 | 0 | 1 | 0 |
| 275 | uncl. Rhodobacteraceae | 0 | 0 | 0 | 0 | 0 | 0 | 0 | 0 | 0 | 2 | 4 | 1 |
| 276 | uncl. Alphaproteobacteria | 0 | 0 | 0 | 0 | 0 | 0 | 0 | 0 | 0 | 1 | 7 | 2 |
| 277 | uncl. Vallitalea | 0 | 0 | 0 | 0 | 0 | 0 | 0 | 0 | 0 | 1 | 2 | 1 |
| 278 | uncl. Gammaproteobacteria | 0 | 0 | 0 | 0 | 0 | 0 | 0 | 0 | 0 | 1 | 2 | 2 |
| 279 | uncl. Bacteria | 0 | 0 | 0 | 1 | 0 | 0 | 0 | 0 | 0 | 0 | 5 | 3 |
| 280 | uncl. Deltaproteobacteria | 0 | 1 | 0 | 2 | 2 | 3 | 1 | 1 | 0 | 0 | 0 | 0 |
| 281 | Gimesia maris | 0 | 0 | 1 | 0 | 0 | 1 | 0 | 0 | 0 | 2 | 3 | 3 |
| 282 | uncl. Deltaproteobacteria | 0 | 0 | 2 | 0 | 0 | 0 | 0 | 0 | 5 | 0 | 2 | 0 |
| 283 | uncl. Saprospirales | 0 | 0 | 1 | 1 | 0 | 3 | 0 | 0 | 0 | 0 | 1 | 0 |
| 284 | uncl. Bacteria | 3 | 1 | 0 | 0 | 0 | 1 | 2 | 2 | 0 | 0 | 0 | 0 |
| 285 | uncl. Flammeovirgaceae | 0 | 0 | 0 | 0 | 0 | 0 | 0 | 0 | 0 | 1 | 1 | 1 |
| 286 | uncl. Alphaproteobacteria | 0 | 0 | 1 | 1 | 3 | 0 | 4 | 0 | 0 | 0 | 0 | 0 |
| 287 | Dokdonia pacifica | 0 | 1 | 0 | 0 | 2 | 0 | 1 | 1 | 0 | 0 | 0 | 0 |
| 288 | uncl. Proteobacteria | 0 | 0 | 6 | 0 | 0 | 1 | 0 | 0 | 0 | 0 | 0 | 0 |
| 289 | Roseibacillus persicicus | 1 | 1 | 1 | 1 | 0 | 0 | 1 | 0 | 1 | 0 | 0 | 0 |
| 290 | Sphingomonas leidyi | 0 | 1 | 0 | 0 | 0 | 3 | 0 | 0 | 1 | 2 | 1 | 1 |
| 291 | uncl. Saprospirales | 0 | 0 | 0 | 0 | 0 | 5 | 0 | 1 | 1 | 0 | 0 | 0 |
| 292 | uncl. Winogradskyella | 1 | 1 | 1 | 0 | 2 | 0 | 0 | 1 | 0 | 0 | 1 | 2 |
| 293 | Nereida ignava | 0 | 0 | 1 | 0 | 0 | 0 | 0 | 0 | 1 | 2 | 1 | 1 |
| 294 | uncl. Bacteria | 1 | 0 | 0 | 2 | 2 | 0 | 1 | 0 | 1 | 0 | 0 | 2 |
| 295 | uncl. Rhodobacteraceae | 0 | 0 | 0 | 0 | 0 | 0 | 0 | 0 | 0 | 2 | 1 | 3 |
| 296 | uncl. Bacteria | 3 | 2 | 2 | 0 | 0 | 0 | 0 | 2 | 0 | 0 | 0 | 0 |
| 297 | uncl. Alphaproteobacteria | 0 | 0 | 0 | 0 | 3 | 4 | 0 | 2 | 0 | 0 | 0 | 0 |
| 298 | uncl. Bacteria | 0 | 2 | 3 | 0 | 0 | 0 | 0 | 0 | 0 | 1 | 0 | 2 |
| 299 | uncl. Flavobacterium | 0 | 0 | 0 | 0 | 0 | 2 | 0 | 0 | 0 | 0 | 0 | 0 |
| 300 | Rubidimonas crustatorum | 1 | 0 | 0 | 0 | 2 | 0 | 0 | 2 | 0 | 0 | 0 | 0 |
| 301 | uncl. Cyanobacteria | 0 | 0 | 0 | 0 | 1 | 0 | 0 | 0 | 0 | 0 | 3 | 2 |
| 302 | uncl. Rhodospirillaceae | 0 | 0 | 0 | 0 | 0 | 1 | 0 | 0 | 0 | 0 | 1 | 3 |
| 303 | uncl. Bacteria | 3 | 1 | 0 | 0 | 0 | 0 | 0 | 0 | 2 | 0 | 1 | 1 |
| 304 | uncl. Alphaproteobacteria | 1 | 1 | 0 | 0 | 0 | 3 | 1 | 0 | 2 | 0 | 0 | 0 |
| 305 | uncl. Saprospirales | 0 | 0 | 0 | 2 | 0 | 2 | 1 | 0 | 1 | 0 | 0 | 0 |
| 306 | uncl. Bacteria | 0 | 0 | 1 | 0 | 0 | 1 | 0 | 0 | 0 | 1 | 3 | 2 |
| 307 | Roseobacter denitrificans | 0 | 0 | 0 | 0 | 2 | 0 | 0 | 0 | 0 | 2 | 11 | 0 |
| 308 | uncl. Bdellovibrio | 0 | 0 | 0 | 0 | 0 | 3 | 3 | 0 | 6 | 0 | 0 | 0 |
| 309 | uncl. Erythrobacteraceae | 1 | 1 | 0 | 0 | 0 | 0 | 0 | 0 | 1 | 1 | 0 | 6 |
| 310 | uncl. Rhodobacteraceae | 0 | 0 | 0 | 1 | 0 | 2 | 0 | 0 | 1 | 0 | 0 | 1 |
| 311 | uncl. Actinobacteria | 1 | 4 | 0 | 1 | 0 | 0 | 0 | 2 | 0 | 0 | 0 | 1 |
| 312 | uncl. Bacteria | 0 | 0 | 2 | 0 | 0 | 4 | 1 | 1 | 0 | 0 | 0 | 0 |
| 313 | uncl. Alphaproteobacteria | 0 | 0 | 0 | 0 | 0 | 0 | 0 | 0 | 0 | 1 | 3 | 4 |
| 314 | uncl. Sphingorhabdus | 1 | 1 | 0 | 0 | 0 | 0 | 1 | 0 | 0 | 0 | 0 | 5 |
| 315 | uncl. Proteobacteria | 0 | 0 | 0 | 0 | 0 | 5 | 0 | 0 | 1 | 1 | 0 | 0 |
| 316 | uncl. Nostocales | 0 | 0 | 0 | 0 | 0 | 1 | 0 | 0 | 0 | 0 | 0 | 1 |
| 317 | uncl. Bacteria | 0 | 1 | 3 | 0 | 0 | 0 | 0 | 0 | 1 | 0 | 0 | 2 |
| 318 | uncl. Proteobacteria | 2 | 1 | 1 | 0 | 0 | 0 | 1 | 0 | 1 | 0 | 0 | 0 |
| 319 | Anderseniella baltica | 0 | 2 | 0 | 0 | 0 | 0 | 0 | 0 | 0 | 2 | 4 | 7 |
| 320 | uncl. Sulfurimonas | 0 | 0 | 0 | 0 | 0 | 0 | 0 | 0 | 0 | 0 | 1 | 2 |
| 321 | uncl. Saprospirales | 0 | 0 | 0 | 1 | 0 | 3 | 0 | 0 | 0 | 0 | 1 | 1 |
| 322 | uncl. Flavobacteriaceae | 0 | 0 | 0 | 0 | 0 | 0 | 0 | 0 | 0 | 1 | 2 | 1 |
| 323 | uncl. Ilumatobacter | 0 | 0 | 1 | 0 | 0 | 0 | 0 | 0 | 1 | 2 | 3 | 0 |
| 324 | uncl. Deltaproteobacteria | 0 | 0 | 3 | 0 | 0 | 2 | 0 | 0 | 2 | 0 | 0 | 0 |
| 325 | uncl. Rubritalea | 0 | 0 | 0 | 1 | 4 | 0 | 0 | 0 | 0 | 0 | 0 | 0 |
| 326 | uncl. Proteobacteria | 0 | 0 | 4 | 0 | 0 | 0 | 0 | 0 | 2 | 0 | 0 | 0 |
| 327 | uncl. Verrucomicrobiaceae | 2 | 0 | 1 | 0 | 0 | 1 | 0 | 1 | 0 | 0 | 0 | 0 |
| 328 | uncl. Hyphomicrobiaceae | 0 | 0 | 0 | 0 | 1 | 5 | 0 | 0 | 0 | 1 | 1 | 0 |
| 329 | uncl. Alphaproteobacteria | 0 | 0 | 0 | 2 | 5 | 0 | 0 | 0 | 0 | 0 | 0 | 0 |
| 330 | uncl. Rhodobacteraceae | 0 | 0 | 0 | 0 | 0 | 0 | 0 | 0 | 0 | 1 | 3 | 1 |
| 331 | uncl. Rhodobacteraceae | 0 | 0 | 0 | 0 | 0 | 0 | 3 | 0 | 0 | 1 | 1 | 0 |
| 332 | Hellea balneolensis | 2 | 1 | 0 | 0 | 1 | 0 | 0 | 0 | 1 | 1 | 1 | 0 |
| 333 | uncl. Rhodobacteraceae | 0 | 0 | 0 | 1 | 1 | 0 | 0 | 0 | 0 | 0 | 1 | 1 |
| 334 | uncl. Alphaproteobacteria | 0 | 0 | 2 | 0 | 0 | 1 | 0 | 0 | 0 | 2 | 1 | 1 |
| 335 | uncl. Alphaproteobacteria | 0 | 1 | 4 | 0 | 0 | 1 | 0 | 0 | 0 | 1 | 0 | 0 |
| 336 | uncl. Bacteria | 0 | 1 | 0 | 0 | 0 | 1 | 0 | 0 | 1 | 1 | 2 | 1 |
| 337 | uncl. Saprospirales | 1 | 0 | 1 | 0 | 0 | 1 | 1 | 0 | 1 | 0 | 0 | 0 |
| 338 | uncl. Bacteria | 0 | 3 | 1 | 0 | 0 | 0 | 0 | 1 | 0 | 0 | 0 | 2 |
| 339 | uncl. Bacteria | 0 | 0 | 0 | 0 | 0 | 0 | 0 | 0 | 0 | 1 | 5 | 0 |
| 340 | uncl. Actinobacteria | 0 | 0 | 0 | 0 | 0 | 0 | 0 | 1 | 0 | 1 | 1 | 2 |
| 341 | Ilyobacter polytropus | 0 | 0 | 0 | 0 | 0 | 0 | 0 | 0 | 0 | 0 | 0 | 1 |
| 342 | uncl. Alphaproteobacteria | 0 | 0 | 0 | 0 | 3 | 0 | 0 | 0 | 0 | 1 | 1 | 1 |
| 343 | Geminicoccus roseus | 0 | 0 | 0 | 0 | 0 | 0 | 0 | 0 | 0 | 2 | 2 | 2 |
| 344 | uncl. Bacteria | 0 | 0 | 0 | 0 | 1 | 0 | 1 | 0 | 0 | 2 | 2 | 0 |
| 345 | uncl. Rhodospirillaceae | 0 | 0 | 0 | 0 | 0 | 0 | 0 | 0 | 0 | 2 | 1 | 0 |

**Table S2.** Taxonomy list of all bacterial OTUs identified from *F. vesiculosus* and the respective seawater reference samples.

| **Phylum** | **Class** | **Order** | **Family** | **Genus** | **Species** |
| --- | --- | --- | --- | --- | --- |
| Acidobacteria | Blastocatellia | uncl. Blastocatellia | uncl. Blastocatellia | uncl. Blastocatellia | uncl. Blastocatellia |
| Actinobacteria | Acidimicrobiia | Acidimicrobiales | Acidimicrobiaceae | Ilumatobacter | Ilumatobacter nonamiensis |
| Actinobacteria | Acidimicrobiia | Acidimicrobiales | Acidimicrobiaceae | Ilumatobacter | uncl. Ilumatobacter |
| Actinobacteria | Acidimicrobiia | Acidimicrobiales | Iamiaceae | uncl. Iamiaceae | uncl. Iamiaceae |
| Actinobacteria | Acidimicrobiia | Acidimicrobiales | uncl. Acidimicrobiales | uncl. Acidimicrobiales | uncl. Acidimicrobiales |
| Actinobacteria | Actinobacteria | Micrococcales | Microbacteriaceae | uncl. Microbacteriaceae | uncl. Microbacteriaceae |
| Actinobacteria | Actinobacteria | uncl. Actinobacteria | uncl. Actinobacteria | uncl. Actinobacteria | uncl. Actinobacteria |
| Actinobacteria | uncl. Actinobacteria | uncl. Actinobacteria | uncl. Actinobacteria | uncl. Actinobacteria | uncl. Actinobacteria |
| Bacteroidetes | Cytophagia | Cytophagales | Flammeovirgaceae | Roseivirga | uncl. Roseivirga |
| Bacteroidetes | Cytophagia | Cytophagales | Flammeovirgaceae | uncl. Flammeovirgaceae | uncl. Flammeovirgaceae |
| Bacteroidetes | Flavobacteriia | Flavobacteriales | Flavobacteriaceae | Dokdonia | Dokdonia eikasta |
| Bacteroidetes | Flavobacteriia | Flavobacteriales | Flavobacteriaceae | Dokdonia | Dokdonia pacifica |
| Bacteroidetes | Flavobacteriia | Flavobacteriales | Flavobacteriaceae | Flavobacterium | uncl. Flavobacterium |
| Bacteroidetes | Flavobacteriia | Flavobacteriales | Flavobacteriaceae | Maribacter | uncl. Maribacter |
| Bacteroidetes | Flavobacteriia | Flavobacteriales | Flavobacteriaceae | Nonlabens | Nonlabens ulvanivorans |
| Bacteroidetes | Flavobacteriia | Flavobacteriales | Flavobacteriaceae | Polaribacter | Polaribacter dokdonensis |
| Bacteroidetes | Flavobacteriia | Flavobacteriales | Flavobacteriaceae | uncl. Flavobacteriaceae | uncl. Flavobacteriaceae |
| Bacteroidetes | Flavobacteriia | Flavobacteriales | Flavobacteriaceae | Winogradskyella | uncl. Winogradskyella |
| Bacteroidetes | Flavobacteriia | Flavobacteriales | uncl. Flavobacteriales | uncl. Flavobacteriales | uncl. Flavobacteriales |
| Bacteroidetes | Saprospiria | Saprospirales | Haliscomenobacteraceae | Portibacter | Portibacter lacus |
| Bacteroidetes | Saprospiria | Saprospirales | Haliscomenobacteraceae | uncl. Haliscomenobacteraceae | uncl. Haliscomenobacteraceae |
| Bacteroidetes | Saprospiria | Saprospirales | Lewinellaceae | Lewinella | Lewinella nigricans |
| Bacteroidetes | Saprospiria | Saprospirales | Lewinellaceae | Lewinella | Lewinella persica |
| Bacteroidetes | Saprospiria | Saprospirales | Lewinellaceae | Lewinella | uncl. Lewinella |
| Bacteroidetes | Saprospiria | Saprospirales | Saprospiraceae | Rubidimonas | Rubidimonas crustatorum |
| Bacteroidetes | Saprospiria | Saprospirales | uncl. Saprospirales | uncl. Saprospirales | uncl. Saprospirales |
| Candidatus Melainabacteria | uncl. Candidatus Melainabacteria | uncl. Candidatus Melainabacteria | uncl. Candidatus Melainabacteria | Vampirovibrio | Vampirovibrio chlorellavorus |
| Chloroflexi | Caldilineae | Caldilineales | Caldilineaceae | Litorilinea | Litorilinea aerophila |
| Cyanobacteria | uncl. Cyanobacteria | Nostocales | uncl. Nostocales | uncl. Nostocales | uncl. Nostocales |
| Cyanobacteria | uncl. Cyanobacteria | Pleurocapsales | Xenococcaceae | Foliisarcina | Foliisarcina bertiogensis |
| Cyanobacteria | uncl. Cyanobacteria | Synechococcales | Leptolyngbyaceae | Trichocoleus | Trichocoleus desertorum |
| Cyanobacteria | uncl. Cyanobacteria | Synechococcales | Synechococcaceae | Synechococcus | Synechococcus rubescens |
| Cyanobacteria | uncl. Cyanobacteria | Synechococcales | uncl. Synechococcales | uncl. Synechococcales | uncl. Synechococcales |
| Cyanobacteria | uncl. Cyanobacteria | uncl. Cyanobacteria | uncl. Cyanobacteria | uncl. Cyanobacteria | uncl. Cyanobacteria |
| Deinococcus-Thermus | Deinococci | Deinococcales | Trueperaceae | Truepera | Truepera radiovictrix |
| Deinococcus-Thermus | Deinococci | Deinococcales | uncl. Deinococcales | uncl. Deinococcales | uncl. Deinococcales |
| Firmicutes | Clostridia | Clostridiales | Defluviitaleaceae | Vallitalea | uncl. Vallitalea |
| Fusobacteria | Fusobacteriia | Fusobacteriales | Fusobacteriaceae | Ilyobacter | Ilyobacter polytropus |
| Fusobacteria | Fusobacteriia | Fusobacteriales | Fusobacteriaceae | uncl. Fusobacteriaceae | uncl. Fusobacteriaceae |
| Planctomycetes | Phycisphaerae | Phycisphaerales | Phycisphaeraceae | Algisphaera | Algisphaera agarilytica |
| Planctomycetes | Planctomycetia | Planctomycetales | Planctomycetaceae | Bythopirellula | Bythopirellula goksoyri |
| Planctomycetes | Planctomycetia | Planctomycetales | Planctomycetaceae | Gimesia | Gimesia maris |
| Planctomycetes | Planctomycetia | Planctomycetales | Planctomycetaceae | uncl. Planctomycetaceae | uncl. Planctomycetaceae |
| Proteobacteria | Alphaproteobacteria | Kiloniellales | Kiloniellaceae | Kiloniella | Kiloniella laminariae |
| Proteobacteria | Alphaproteobacteria | Kiloniellales | Kiloniellaceae | Kiloniella | uncl. Kiloniella |
| Proteobacteria | Alphaproteobacteria | Kordiimonadales | Kordiimonadaceae | Emcibacter | Emcibacter nanhaiensis |
| Proteobacteria | Alphaproteobacteria | Magnetococcales | Magnetococcaceae | Magnetococcus | Magnetococcus marinus |
| Proteobacteria | Alphaproteobacteria | Parvularculales | Parvularculaceae | Parvularcula | Parvularcula flava |
| Proteobacteria | Alphaproteobacteria | Pelagibacterales | Pelagibacteraceae | Candidatus Pelagibacter | Candidatus Pelagibacter ubique |
| Proteobacteria | Alphaproteobacteria | Rhizobiales | Hyphomicrobiaceae | Filomicrobium | uncl. Filomicrobium |
| Proteobacteria | Alphaproteobacteria | Rhizobiales | Hyphomicrobiaceae | Hyphomicrobium | uncl. Hyphomicrobium |
| Proteobacteria | Alphaproteobacteria | Rhizobiales | Hyphomicrobiaceae | uncl. Hyphomicrobiaceae | uncl. Hyphomicrobiaceae |
| Proteobacteria | Alphaproteobacteria | Rhizobiales | Phyllobacteriaceae | Pseudahrensia | uncl. Pseudahrensia |
| Proteobacteria | Alphaproteobacteria | Rhizobiales | Phyllobacteriaceae | uncl. Phyllobacteriaceae | uncl. Phyllobacteriaceae |
| Proteobacteria | Alphaproteobacteria | Rhizobiales | Rhodobiaceae | Anderseniella | Anderseniella baltica |
| Proteobacteria | Alphaproteobacteria | Rhizobiales | uncl. Rhizobiales | uncl. Rhizobiales | uncl. Rhizobiales |
| Proteobacteria | Alphaproteobacteria | Rhodobacterales | Hyphomonadaceae | Fretibacter | Fretibacter rubidus |
| Proteobacteria | Alphaproteobacteria | Rhodobacterales | Hyphomonadaceae | Hellea | Hellea balneolensis |
| Proteobacteria | Alphaproteobacteria | Rhodobacterales | Hyphomonadaceae | Hirschia | Hirschia baltica |
| Proteobacteria | Alphaproteobacteria | Rhodobacterales | Hyphomonadaceae | Hyphomonas | uncl. Hyphomonas |
| Proteobacteria | Alphaproteobacteria | Rhodobacterales | Hyphomonadaceae | Litorimonas | Litorimonas cladophorae |
| Proteobacteria | Alphaproteobacteria | Rhodobacterales | Hyphomonadaceae | Litorimonas | Litorimonas taeanensis |
| Proteobacteria | Alphaproteobacteria | Rhodobacterales | Hyphomonadaceae | Robiginitomaculum | Robiginitomaculum antarcticum |
| Proteobacteria | Alphaproteobacteria | Rhodobacterales | Hyphomonadaceae | uncl. Hyphomonadaceae | uncl. Hyphomonadaceae |
| Proteobacteria | Alphaproteobacteria | Rhodobacterales | Rhodobacteraceae | Ahrensia | Ahrensia kielensis |
| Proteobacteria | Alphaproteobacteria | Rhodobacterales | Rhodobacteraceae | Boseongicola | Boseongicola aestuarii |
| Proteobacteria | Alphaproteobacteria | Rhodobacterales | Rhodobacteraceae | Litoreibacter | Litoreibacter meonggei |
| Proteobacteria | Alphaproteobacteria | Rhodobacterales | Rhodobacteraceae | Loktanella | uncl. Loktanella |
| Proteobacteria | Alphaproteobacteria | Rhodobacterales | Rhodobacteraceae | Nereida | Nereida ignava |
| Proteobacteria | Alphaproteobacteria | Rhodobacterales | Rhodobacteraceae | Octadecabacter | Octadecabacter ponticola |
| Proteobacteria | Alphaproteobacteria | Rhodobacterales | Rhodobacteraceae | Pacificibacter | Pacificibacter marinus |
| Proteobacteria | Alphaproteobacteria | Rhodobacterales | Rhodobacteraceae | Planktomarina | Planktomarina temperata |
| Proteobacteria | Alphaproteobacteria | Rhodobacterales | Rhodobacteraceae | Pseudorhodobacter | uncl. Pseudorhodobacter |
| Proteobacteria | Alphaproteobacteria | Rhodobacterales | Rhodobacteraceae | Roseobacter | Roseobacter denitrificans |
| Proteobacteria | Alphaproteobacteria | Rhodobacterales | Rhodobacteraceae | Roseobacter | Roseobacter litoralis |
| Proteobacteria | Alphaproteobacteria | Rhodobacterales | Rhodobacteraceae | Sulfitobacter | uncl. Sulfitobacter |
| Proteobacteria | Alphaproteobacteria | Rhodobacterales | Rhodobacteraceae | uncl. Rhodobacteraceae | uncl. Rhodobacteraceae |
| Proteobacteria | Alphaproteobacteria | Rhodospirillales | Rhodospirillaceae | Nisaea | Nisaea denitrificans |
| Proteobacteria | Alphaproteobacteria | Rhodospirillales | Rhodospirillaceae | uncl. Rhodospirillaceae | uncl. Rhodospirillaceae |
| Proteobacteria | Alphaproteobacteria | Rhodospirillales | uncl. Rhodospirillales | uncl. Rhodospirillales | uncl. Rhodospirillales |
| Proteobacteria | Alphaproteobacteria | Rickettsiales | Rickettsiaceae | Rickettsia | uncl. Rickettsia |
| Proteobacteria | Alphaproteobacteria | Sphingomonadales | Erythrobacteraceae | Altererythrobacter | uncl. Altererythrobacter |
| Proteobacteria | Alphaproteobacteria | Sphingomonadales | Erythrobacteraceae | Erythrobacter | uncl. Erythrobacter |
| Proteobacteria | Alphaproteobacteria | Sphingomonadales | Erythrobacteraceae | uncl. Erythrobacteraceae | uncl. Erythrobacteraceae |
| Proteobacteria | Alphaproteobacteria | Sphingomonadales | Sphingomonadaceae | Sphingomonas | Sphingomonas leidyi |
| Proteobacteria | Alphaproteobacteria | Sphingomonadales | Sphingomonadaceae | Sphingorhabdus | Sphingorhabdus litoris |
| Proteobacteria | Alphaproteobacteria | Sphingomonadales | Sphingomonadaceae | Sphingorhabdus | uncl. Sphingorhabdus |
| Proteobacteria | Alphaproteobacteria | Sphingomonadales | Sphingomonadaceae | uncl. Sphingomonadaceae | uncl. Sphingomonadaceae |
| Proteobacteria | Alphaproteobacteria | uncl. Alphaproteobacteria | uncl. Alphaproteobacteria | Geminicoccus | Geminicoccus roseus |
| Proteobacteria | Alphaproteobacteria | uncl. Alphaproteobacteria | uncl. Alphaproteobacteria | uncl. Alphaproteobacteria | uncl. Alphaproteobacteria |
| Proteobacteria | Deltaproteobacteria | Desulfobacterales | Desulfobacteraceae | uncl. Desulfobacteraceae | uncl. Desulfobacteraceae |
| Proteobacteria | Deltaproteobacteria | Myxococcales | Nannocystaceae | uncl. Nannocystaceae | uncl. Nannocystaceae |
| Proteobacteria | Deltaproteobacteria | uncl. Deltaproteobacteria | uncl. Deltaproteobacteria | uncl. Deltaproteobacteria | uncl. Deltaproteobacteria |
| Proteobacteria | Epsilonproteobacteria | Campylobacterales | Campylobacteraceae | Arcobacter | Arcobacter cryaerophilus |
| Proteobacteria | Epsilonproteobacteria | Campylobacterales | Campylobacteraceae | Arcobacter | uncl. Arcobacter |
| Proteobacteria | Epsilonproteobacteria | Campylobacterales | Helicobacteraceae | Sulfurimonas | Sulfurimonas autotrophica |
| Proteobacteria | Epsilonproteobacteria | Campylobacterales | Helicobacteraceae | Sulfurimonas | uncl. Sulfurimonas |
| Proteobacteria | Epsilonproteobacteria | uncl. Epsilonproteobacteria | uncl. Epsilonproteobacteria | Sulfurovum | uncl. Sulfurovum |
| Proteobacteria | Gammaproteobacteria | Chromatiales | Granulosicoccaceae | Granulosicoccus | uncl. Granulosicoccus |
| Proteobacteria | Gammaproteobacteria | uncl. Gammaproteobacteria | uncl. Gammaproteobacteria | uncl. Gamma-proteobacteria | uncl. Gamma-proteobacteria |
| Proteobacteria | Oligoflexia | Bdellovibrionales | Bdellovibrionaceae | Bdellovibrio | uncl. Bdellovibrio |
| Proteobacteria | uncl. Proteobacteria | uncl. Proteobacteria | uncl. Proteobacteria | uncl. Proteobacteria | uncl. Proteobacteria |
| uncl. Bacteria | uncl. Bacteria | uncl. Bacteria | uncl. Bacteria | uncl. Bacteria | uncl. Bacteria |
| Verrucomicrobia | Verrucomicrobiae | Verrucomicrobiales | Rubritaleaceae | Rubritalea | Rubritalea marina |
| Verrucomicrobia | Verrucomicrobiae | Verrucomicrobiales | Rubritaleaceae | Rubritalea | uncl. Rubritalea |
| Verrucomicrobia | Verrucomicrobiae | Verrucomicrobiales | Verrucomicrobiaceae | Luteolibacter | Luteolibacter algae |
| Verrucomicrobia | Verrucomicrobiae | Verrucomicrobiales | Verrucomicrobiaceae | Luteolibacter | uncl. Luteolibacter |
| Verrucomicrobia | Verrucomicrobiae | Verrucomicrobiales | Verrucomicrobiaceae | Roseibacillus | Roseibacillus persicicus |
| Verrucomicrobia | Verrucomicrobiae | Verrucomicrobiales | Verrucomicrobiaceae | uncl. Verruco-microbiaceae | uncl. Verrucomicrobiaceae |

**Table S3.** Cumulative abundance counts for the taxonomy rank family observed for thallus, tip and whole seaweed samples of *F. vesiculosus* and ambient seawater samples. The average number of sequence reads per OTUs was calculated for three respective replicates per sample. Subsequently, numbers for all OTUs of a bacterial class were summed up and their average percentage in comparison to all detected OTUs in the respective sample type was calculated.

| **Family** | **Thallus** | **Tip** | **Whole seaweed** | **Seawater** | **Seaweed samples combined** |
| --- | --- | --- | --- | --- | --- |
| Acidimicrobiaceae | 0.5 | 0.2 | 0.6 | 0.2 | 0.4 |
| Bdellovibrionaceae | 0.0 | 0.1 | 0.1 | 0.0 | 0.1 |
| Caldilineaceae | 0.1 | 0.2 | 0.0 | 0.3 | 0.1 |
| Campylobacteraceae | 0.0 | 0.0 | 0.0 | 0.9 | 0.0 |
| Defluviitaleaceae | 0.0 | 0.0 | 0.0 | 0.1 | 0.0 |
| Desulfobacteraceae | 0.1 | 0.1 | 0.1 | 0.0 | 0.1 |
| Erythrobacteraceae | 30.6 | 10.3 | 20.0 | 1.7 | 20.3 |
| Flammeovirgaceae | 0.0 | 0.0 | 0.0 | 0.1 | 0.0 |
| Flavobacteriaceae | 0.9 | 0.8 | 0.9 | 0.8 | 0.8 |
| Fusobacteriaceae | 0.0 | 0.0 | 0.0 | 0.2 | 0.0 |
| Granulosicoccaceae | 0.0 | 0.0 | 0.0 | 0.0 | 0.0 |
| Haliscomenobacteraceae | 0.3 | 0.2 | 0.2 | 0.0 | 0.2 |
| Helicobacteraceae | 0.0 | 0.0 | 0.0 | 0.2 | 0.0 |
| Hyphomicrobiaceae | 1.5 | 0.6 | 1.4 | 0.2 | 1.2 |
| Hyphomonadaceae | 8.3 | 20.1 | 12.1 | 2.4 | 13.6 |
| Iamiaceae | 0.9 | 0.4 | 0.7 | 0.1 | 0.7 |
| Kiloniellaceae | 0.1 | 0.1 | 0.2 | 0.3 | 0.1 |
| Kordiimonadaceae | 0.0 | 0.0 | 0.0 | 0.2 | 0.0 |
| Leptolyngbyaceae | 2.0 | 0.2 | 1.1 | 0.1 | 1.1 |
| Lewinellaceae | 0.2 | 0.2 | 0.1 | 0.0 | 0.2 |
| Magnetococcaceae | 0.0 | 0.1 | 0.0 | 0.0 | 0.1 |
| Microbacteriaceae | 0.0 | 0.0 | 0.0 | 2.0 | 0.0 |
| Nannocystaceae | 0.3 | 0.0 | 0.1 | 0.0 | 0.2 |
| Parvularculaceae | 0.5 | 0.3 | 0.2 | 0.0 | 0.3 |
| Pelagibacteraceae | 0.0 | 0.0 | 0.1 | 30.9 | 0.1 |
| Phycisphaeraceae | 4.3 | 0.6 | 1.5 | 0.2 | 2.1 |
| Phyllobacteriaceae | 1.1 | 1.1 | 0.9 | 1.0 | 1.0 |
| Planctomycetaceae | 2.7 | 11.1 | 8.3 | 0.6 | 7.3 |
| Rhodobacteraceae | 21.3 | 27.4 | 24.5 | 35.0 | 24.5 |
| Rhodobiaceae | 0.1 | 0.0 | 0.0 | 0.2 | 0.1 |
| Rhodospirillaceae | 0.0 | 0.0 | 0.0 | 5.2 | 0.0 |
| Rickettsiaceae | 0.0 | 0.0 | 0.0 | 0.4 | 0.0 |
| Rubritaleaceae | 0.1 | 0.5 | 0.3 | 0.1 | 0.3 |
| Saprospiraceae | 0.0 | 0.1 | 0.1 | 0.0 | 0.1 |
| Sphingomonadaceae | 1.4 | 0.4 | 0.4 | 0.3 | 0.9 |
| Synechococcaceae | 0.0 | 0.0 | 0.0 | 1.1 | 0.0 |
| Trueperaceae | 0.1 | 0.0 | 0.0 | 0.0 | 0.1 |
| uncl. Acidimicrobiales | 0.2 | 0.1 | 0.1 | 0.1 | 0.1 |
| uncl. Actinobacteria | 0.4 | 0.3 | 0.3 | 0.0 | 0.3 |
| uncl. Alphaproteobacteria | 3.2 | 6.5 | 4.3 | 5.9 | 4.7 |
| uncl. Bacteria | 5.7 | 4.4 | 4.2 | 2.4 | 4.8 |
| uncl. Blastocatellia | 0.6 | 0.0 | 0.0 | 0.1 | 0.6 |
| uncl. Candidatus Melainabacteria | 0.2 | 0.0 | 0.1 | 0.0 | 0.1 |
| uncl. Cyanobacteria | 1.2 | 3.1 | 2.2 | 4.3 | 2.2 |
| uncl. Deinococcales | 0.9 | 0.4 | 0.4 | 0.1 | 0.6 |
| uncl. Deltaproteobacteria | 1.7 | 1.1 | 1.6 | 0.1 | 1.5 |
| uncl. Epsilonproteobacteria | 0.0 | 0.0 | 0.0 | 0.3 | 0.0 |
| uncl. Flavobacteriales | 0.0 | 0.0 | 0.0 | 0.6 | 0.0 |
| uncl. Gammaproteobacteria | 0.0 | 0.0 | 0.0 | 0.2 | 0.0 |
| uncl. Nostocales | 0.0 | 0.1 | 0.0 | 0.1 | 0.0 |
| uncl. Proteobacteria | 1.6 | 1.7 | 1.7 | 0.7 | 1.7 |
| uncl. Rhizobiales | 0.4 | 1.2 | 0.7 | 0.3 | 0.8 |
| uncl. Rhodospirillales | 0.0 | 0.0 | 0.0 | 0.9 | 0.0 |
| uncl. Saprospirales | 0.3 | 0.6 | 0.4 | 0.1 | 0.4 |
| uncl. Synechococcales | 4.3 | 5.6 | 8.6 | 0.1 | 6.2 |
| Verrucomicrobiaceae | 0.2 | 0.4 | 0.2 | 0.0 | 0.3 |
| Xenococcaceae | 1.7 | 0.1 | 0.9 | 0.1 | 0.9 |

**Table S4.** OTUs exclusively detected in *F. vesiculosus* samples. All OTUs present in at least one of the nine replicates of the different *F. vesiculosus* samples (thallus, tip, whole seaweed) are shown here as exclusive *F. vesiculosus* OTUs.

*: considered as core OTUs of the sampled *F. vesiculosus* since they were detected in all nine *F. vesiculosus* replicates.

| **OTU** | **No. se-quence reads** | **Phylum** | **Class** | **Order** | **Family** | **Genus** | **Species** |
| --- | --- | --- | --- | --- | --- | --- | --- |
| 50* | 100 | Proteobacteria | Deltaproteo-bacteria | uncl. Deltaproteo-bacteria | uncl. Deltaproteo-bacteria | uncl. Deltaproteo-bacteria | uncl. Deltaproteobacteria |
| 51 | 65 | Verruco-microbia | Verruco-microbiae | Verruco-microbiales | Rubritaleaceae | Rubritalea | uncl. Rubritalea |
| 72 | 67 | Proteobacteria | Alphaproteo-bacteria | uncl. Alphaproteo-bacteria | uncl. Alphaproteo-bacteria | uncl. Alphaproteo-bacteria | uncl. Alphaproteobacteria |
| 74 | 47 | Bacteroidetes | Saprospiria | Saprospirales | uncl. Saprospirales | uncl. Saprospirales | uncl. Saprospirales |
| 86 | 51 | uncl. Bacteria | uncl. Bacteria | uncl. Bacteria | uncl. Bacteria | uncl. Bacteria | uncl. Bacteria |
| 92* | 96 | Proteobacteria | Alphaproteo-bacteria | Parvularculales | Parvularculaceae | Parvularcula | Parvularcula flava |
| 102 | 48 | Proteobacteria | Alphaproteo-bacteria | uncl. Alphaproteo-bacteria | uncl. Alphaproteo-bacteria | uncl. Alphaproteo-bacteria | uncl. Alphaproteobacteria |
| 103 | 27 | Bacteroidetes | Flavobacteriia | Flavobacteriales | Flavobacteriaceae | uncl. Flavobacteriaceae | uncl. Flavobacteriaceae |
| 106 | 30 | Proteobacteria | Alphaproteo-bacteria | Rhodobacterales | Rhodo-bacteraceae | uncl. Rhodobacteraceae | uncl. Rhodobacteraceae |
| 110 | 23 | Proteobacteria | Deltaproteo-bacteria | Desulfobacterales | Desulfobacteraceae | uncl. Desulfo-bacteraceae | uncl. Desulfobacteraceae |
| 120 | 46 | Actino-bacteria | uncl. Actinobacteria | uncl. Actinobacteria | uncl. Actinobacteria | uncl. Actinobacteria | uncl. Actinobacteria |
| 132 | 20 | Proteobacteria | Deltaproteo-bacteria | Myxococcales | Nannocystaceae | uncl. Nannocystaceae | uncl. Nannocystaceae |
| 145 | 25 | Proteobacteria | Alphaproteo-bacteria | Sphingomonadales | Sphingo-monadaceae | uncl. Sphingo-monadaceae | uncl. Sphingomonadaceae |
| 146 | 35 | Actino-bacteria | uncl. Actinobacteria | uncl. Actinobacteria | uncl. Actinobacteria | uncl. Actinobacteria | uncl. Actinobacteria |
| 153 | 27 | Proteobacteria | Alphaproteo-bacteria | uncl. Alphaproteo-bacteria | uncl. Alphaproteo-bacteria | uncl. Alphaproteo-bacteria | uncl. Alphaproteobacteria |
| 155 | 22 | Proteobacteria | Alphaproteo-bacteria | Rhizobiales | uncl. Rhizobiales | uncl. Rhizobiales | uncl. Rhizobiales |
| 156 | 16 | Bacteroidetes | Flavobacteriia | Flavobacteriales | Flavobacteriaceae | Dokdonia | Dokdonia eikasta |
| 157* | 26 | Cyanobacteria | uncl. Cyanobacteria | Synechococcales | uncl. Synechococcales | uncl. Synechococcales | uncl. Synechococcales |
| 161* | 23 | Bacteroidetes | Saprospiria | Saprospirales | Haliscomeno-bacteraceae | uncl. Haliscomeno-bacteraceae | uncl. Haliscomeno-bacteraceae |
| 164 | 23 | Bacteroidetes | Saprospiria | Saprospirales | Haliscomeno-bacteraceae | Portibacter | Portibacter lacus |
| 165 | 16 | Bacteroidetes | Flavobacteriia | Flavobacteriales | Flavobacteriaceae | uncl. Flavobacteriaceae | uncl. Flavobacteriaceae |
| 170 | 20 | Proteobacteria | Deltaproteo-bacteria | uncl. Deltaproteo-bacteria | uncl. Deltaproteo-bacteria | uncl. Deltaproteo-bacteria | uncl. Deltaproteobacteria |
| 171 | 19 | uncl. Bacteria | uncl. Bacteria | uncl. Bacteria | uncl. Bacteria | uncl. Bacteria | uncl. Bacteria |
| 172 | 20 | uncl. Bacteria | uncl. Bacteria | uncl. Bacteria | uncl. Bacteria | uncl. Bacteria | uncl. Bacteria |
| 177 | 21 | Proteobacteria | Alphaproteo-bacteria | uncl. Alphaproteo-bacteria | uncl. Alphaproteo-bacteria | uncl. Alphaproteo-bacteria | uncl. Alphaproteobacteria |
| 179 | 18 | Candidatus Melaina-bacteria | uncl. Candidatus Melainabacteria | uncl. Candidatus Melainabacteria | uncl. Candidatus Melainabacteria | Vampirovibrio | Vampirovibrio chlorellavorus |
| 187 | 18 | uncl. Bacteria | uncl. Bacteria | uncl. Bacteria | uncl. Bacteria | uncl. Bacteria | uncl. Bacteria |
| 193 | 14 | Bacteroidetes | Saprospiria | Saprospirales | Lewinellaceae | Lewinella | Lewinella persica |
| 194 | 12 | Proteobacteria | Alphaproteo-bacteria | Rhodobacterales | Rhodo-bacteraceae | uncl. Rhodobacteraceae | uncl. Rhodobacteraceae |
| 195 | 5 | Bacteroidetes | Flavobacteriia | Flavobacteriales | Flavobacteriaceae | Flavobacterium | uncl. Flavobacterium |
| 198 | 14 | Proteobacteria | uncl. Proteobacteria | uncl. Proteobacteria | uncl. Proteobacteria | uncl. Proteobacteria | uncl. Proteobacteria |
| 202 | 11 | Verruco-microbia | Verruco-microbiae | Verruco-microbiales | Verruco-microbiaceae | Luteolibacter | Luteolibacter algae |
| 205* | 11 | Bacteroidetes | Saprospiria | Saprospirales | uncl. Saprospirales | uncl. Saprospirales | uncl. Saprospirales |
| 206 | 14 | Bacteroidetes | Saprospiria | Saprospirales | Haliscomeno-bacteraceae | Portibacter | Portibacter lacus |
| 207 | 12 | Proteobacteria | uncl. Proteobacteria | uncl. Proteobacteria | uncl. Proteobacteria | uncl. Proteobacteria | uncl. Proteobacteria |
| 210 | 10 | Proteobacteria | Gammaproteo-bacteria | Chromatiales | Granulosi-coccaceae | Granulosicoccus | uncl. Granulosicoccus |
| 214 | 14 | uncl. Bacteria | uncl. Bacteria | uncl. Bacteria | uncl. Bacteria | uncl. Bacteria | uncl. Bacteria |
| 215 | 9 | Bacteroidetes | Saprospiria | Saprospirales | uncl. Saprospirales | uncl. Saprospirales | uncl. Saprospirales |
| 218 | 16 | Proteobacteria | Alphaproteo-bacteria | uncl. Alphaproteo-bacteria | uncl. Alphaproteo-bacteria | uncl. Alphaproteo-bacteria | uncl. Alphaproteobacteria |
| 219 | 13 | Proteobacteria | Deltaproteo-bacteria | uncl. Deltaproteo-bacteria | uncl. Deltaproteo-bacteria | uncl. Deltaproteo-bacteria | uncl. Deltaproteobacteria |
| 225 | 12 | Proteobacteria | Deltaproteo-bacteria | uncl. Deltaproteo-bacteria | uncl. Deltaproteo-bacteria | uncl. Delta-proteobacteria | uncl. Deltaproteobacteria |
| 234 | 7 | Proteobacteria | Deltaproteo-bacteria | Desulfobacterales | Desulfo-bacteraceae | uncl. Desulfo-bacteraceae | uncl. Desulfobacteraceae |
| 235 | 11 | Proteobacteria | Deltaproteo-bacteria | uncl. Deltaproteo-bacteria | uncl. Deltaproteo-bacteria | uncl. Deltaproteo-bacteria | uncl. Deltaproteobacteria |
| 241 | 12 | Proteobacteria | Alphaproteo-bacteria | Rhizobiales | uncl. Rhizobiales | uncl. Rhizobiales | uncl. Rhizobiales |
| 247 | 11 | uncl. Bacteria | uncl. Bacteria | uncl. Bacteria | uncl. Bacteria | uncl. Bacteria | uncl. Bacteria |
| 248 | 10 | Proteobacteria | uncl. Proteobacteria | uncl. Proteobacteria | uncl. Proteobacteria | uncl. Proteobacteria | uncl. Proteobacteria |
| 252 | 6 | Proteobacteria | Alphaproteo-bacteria | Magnetococcales | Magneto-coccaceae | Magnetococcus | Magnetococcus marinus |
| 254 | 11 | uncl. Bacteria | uncl. Bacteria | uncl. Bacteria | uncl. Bacteria | uncl. Bacteria | uncl. Bacteria |
| 257 | 11 | Proteobacteria | Alphaproteo-bacteria | uncl. Alphaproteo-bacteria | uncl. Alphaproteo-bacteria | uncl. Alphaproteo-bacteria | uncl. Alphaproteobacteria |
| 262 | 7 | Bacteroidetes | Saprospiria | Saprospirales | uncl. Saprospirales | uncl. Saprospirales | uncl. Saprospirales |
| 263 | 7 | Bacteroidetes | Saprospiria | Saprospirales | Lewinellaceae | Lewinella | uncl. Lewinella |
| 265 | 9 | uncl. Bacteria | uncl. Bacteria | uncl. Bacteria | uncl. Bacteria | uncl. Bacteria | uncl. Bacteria |
| 266 | 5 | Proteobacteria | Deltaproteo-bacteria | Desulfobacterales | Desulfo-bacteraceae | uncl. Desulfo-bacteraceae | uncl. Desulfobacteraceae |
| 269 | 6 | Bacteroidetes | Saprospiria | Saprospirales | uncl. Saprospirales | uncl. Saprospirales | uncl. Saprospirales |
| 271 | 9 | Proteobacteria | uncl. Proteobacteria | uncl. Proteobacteria | uncl. Proteobacteria | uncl. Proteobacteria | uncl. Proteobacteria |
| 272 | 7 | Bacteroidetes | Saprospiria | Saprospirales | Lewinellaceae | Lewinella | uncl. Lewinella |
| 273 | 8 | Bacteroidetes | Saprospiria | Saprospirales | uncl. Saprospirales | uncl. Saprospirales | uncl. Saprospirales |
| 280 | 10 | Proteobacteria | Deltaproteo-bacteria | uncl. Deltaproteo-bacteria | uncl. Deltaproteo-bacteria | uncl. Deltaproteo-bacteria | uncl. Deltaproteobacteria |
| 284 | 9 | uncl. Bacteria | uncl. Bacteria | uncl. Bacteria | uncl. Bacteria | uncl. Bacteria | uncl. Bacteria |
| 286 | 9 | Proteobacteria | Alphaproteo-bacteria | uncl. Alphaproteo-bacteria | uncl. Alphaproteo-bacteria | uncl. Alphaproteo-bacteria | uncl. Alphaproteobacteria |
| 287 | 5 | Bacteroidetes | Flavobacteriia | Flavobacteriales | Flavobacteriaceae | Dokdonia | Dokdonia pacifica |
| 288 | 7 | Proteobacteria | uncl. Proteobacteria | uncl. Proteobacteria | uncl. Proteobacteria | uncl. Proteobacteria | uncl. Proteobacteria |
| 289 | 6 | Verruco-microbia | Verruco-microbiae | Verruco-microbiales | Verruco-microbiaceae | Roseibacillus | Roseibacillus persicicus |
| 291 | 7 | Bacteroidetes | Saprospiria | Saprospirales | uncl. Saprospirales | uncl. Saprospirales | uncl. Saprospirales |
| 296 | 9 | uncl. Bacteria | uncl. Bacteria | uncl. Bacteria | uncl. Bacteria | uncl. Bacteria | uncl. Bacteria |
| 297 | 9 | Proteobacteria | Alphaproteo-bacteria | uncl. Alphaproteo-bacteria | uncl. Alphaproteo-bacteria | uncl. Alphaproteo-bacteria | uncl. Alphaproteobacteria |
| 299 | 2 | Bacteroidetes | Flavobacteriia | Flavobacteriales | Flavobacteriaceae | Flavobacterium | uncl. Flavobacterium |
| 300 | 5 | Bacteroidetes | Saprospiria | Saprospirales | Saprospiraceae | Rubidimonas | Rubidimonas crustatorum |
| 304 | 8 | Proteobacteria | Alphaproteo-bacteria | uncl. Alphaproteo-bacteria | uncl. Alphaproteo-bacteria | uncl. Alphaproteo-bacteria | uncl. Alphaproteobacteria |
| 305 | 6 | Bacteroidetes | Saprospiria | Saprospirales | uncl. Saprospirales | uncl. Saprospirales | uncl. Saprospirales |
| 308 | 12 | Proteobacteria | Oligoflexia | Bdellovibrionales | Bdello-vibrionaceae | Bdellovibrio | uncl. Bdellovibrio |
| 312 | 8 | uncl. Bacteria | uncl. Bacteria | uncl. Bacteria | uncl. Bacteria | uncl. Bacteria | uncl. Bacteria |
| 318 | 6 | Proteobacteria | uncl. Proteobacteria | uncl. Proteobacteria | uncl. Proteobacteria | uncl. Proteobacteria | uncl. Proteobacteria |
| 324 | 7 | Proteobacteria | Deltaproteo-bacteria | uncl. Deltaproteo-bacteria | uncl. Deltaproteo-bacteria | uncl. Deltaproteo-bacteria | uncl. Deltaproteobacteria |
| 325 | 5 | Verruco-microbia | Verruco-microbiae | Verruco-microbiales | Rubritaleaceae | Rubritalea | uncl. Rubritalea |
| 326 | 6 | Proteobacteria | uncl. Proteobacteria | uncl. Proteobacteria | uncl. Proteobacteria | uncl. Proteobacteria | uncl. Proteobacteria |
| 327 | 5 | Verruco-microbia | Verruco-microbiae | Verruco-microbiales | Verruco-microbiaceae | uncl. Verruco-microbiaceae | uncl. Verrucomicrobiaceae |
| 329 | 7 | Proteobacteria | Alphaproteo-bacteria | uncl. Alphaproteo-bacteria | uncl. Alphaproteo-bacteria | uncl. Alphaproteo-bacteria | uncl. Alphaproteobacteria |
| 337 | 5 | Bacteroidetes | Saprospiria | Saprospirales | uncl. Saprospirales | uncl. Saprospirales | uncl. Saprospirales |

**Table S5.** Results of SIMPER analysis to detect the OTUs contributing most to the differences observed between different samples. SIMPER analysis was performed by considering only the most abundant OTUs (80% threshold) on the basis of the Bray-Curtis similarity index.

| **Thallus vs. tip** | | | |
| --- | --- | --- | --- |
| **OTU** | **Contrib. %** | **Mean thallus** | **Mean tip** |
| 1 | 13.630 | 630 | 228 |
| 2 | 12.580 | 115 | 488 |
| 3 | 10.040 | 64.3 | 372 |
| 9 | 8.214 | 21.3 | 275 |
| 15 | 7.228 | 237 | 15.3 |
| 7 | 5.221 | 76.7 | 239 |
| 12 | 3.474 | 148 | 111 |
| 13 | 3.029 | 113 | 22.3 |
| 8 | 2.950 | 157 | 67 |
| 5 | 2.652 | 73 | 153 |
| 34 | 2.633 | 85 | 3.67 |
| 6 | 2.532 | 111 | 122 |
| 10 | 2.462 | 94.3 | 161 |
| 23 | 2.048 | 68.7 | 6 |
| 14 | 1.955 | 36.3 | 90.7 |
| 24 | 1.752 | 57.3 | 3.67 |
| 18 | 1.505 | 30.3 | 77 |
| 36 | 1.430 | 45.7 | 2.33 |
| 21 | 1.365 | 16 | 41.3 |
| 22 | 1.232 | 51.3 | 13.7 |
| 27 | 1.210 | 41.3 | 18.7 |
| 33 | 1.182 | 6.67 | 43.7 |
| 16 | 1.094 | 48.7 | 30.3 |
| 41 | 1.092 | 40 | 7 |
| 25 | 0.939 | 35.3 | 17 |
| 17 | 0.821 | 41 | 54 |
| 26 | 0.790 | 30.7 | 25.7 |
| 28 | 0.704 | 28.3 | 16.3 |
| 32 | 0.633 | 28.7 | 14.7 |
| 30 | 0.598 | 28.7 | 12.3 |
| 44 | 0.523 | 4 | 19.7 |
| 45 | 0.513 | 6.67 | 21.7 |
| 20 | 0.455 | 39.3 | 27.3 |
| 37 | 0.411 | 19.7 | 7.33 |
| 43 | 0.292 | 12.7 | 5.33 |
| 39 | 0.273 | 14.3 | 7.67 |
| 29 | 0.266 | 23.3 | 20 |
| 46 | 0.255 | 4.33 | 11.3 |
| 40 | 0.012 | 0 | 0.333 |
| 4 | 0.012 | 0.333 | 0 |
| 31 | 0.000 | 0 | 0 |
| 11 | 0.000 | 0 | 0 |
| 42 | 0.000 | 0 | 0 |
| 38 | 0.000 | 0 | 0 |
| 19 | 0.000 | 0 | 0 |
| 35 | 0.000 | 0 | 0 |
| **Thallus vs. whole seaweed** | | | |
| **OTU** | **Contrib. %** | **Mean thallus** | **Mean whole seaweed** |
| 3 | 11.030 | 64.3 | 258 |
| 2 | 9.905 | 115 | 293 |
| 1 | 9.184 | 630 | 472 |
| 15 | 8.566 | 237 | 93 |
| 6 | 5.302 | 111 | 206 |
| 12 | 4.366 | 148 | 131 |
| 7 | 4.322 | 76.7 | 150 |
| 9 | 4.106 | 21.3 | 96 |
| 18 | 3.479 | 30.3 | 94.7 |
| 13 | 3.332 | 113 | 51.3 |
| 34 | 2.943 | 85 | 29.7 |
| 17 | 2.891 | 41 | 78.7 |
| 5 | 2.442 | 73 | 109 |
| 23 | 2.363 | 68.7 | 38 |
| 8 | 2.317 | 157 | 117 |
| 24 | 2.306 | 57.3 | 32 |
| 36 | 2.266 | 45.7 | 6.33 |
| 10 | 2.160 | 94.3 | 134 |
| 28 | 1.579 | 28.3 | 38.3 |
| 27 | 1.544 | 41.3 | 35 |
| 25 | 1.490 | 35.3 | 36 |
| 14 | 1.430 | 36.3 | 62 |
| 21 | 1.401 | 16 | 26.7 |
| 22 | 1.166 | 51.3 | 29.7 |
| 41 | 0.904 | 40 | 29.3 |
| 26 | 0.902 | 30.7 | 24.3 |
| 30 | 0.862 | 28.7 | 14.3 |
| 33 | 0.823 | 6.67 | 20.3 |
| 16 | 0.753 | 48.7 | 59.3 |
| 20 | 0.578 | 39.3 | 33.7 |
| 32 | 0.574 | 28.7 | 25 |
| 37 | 0.539 | 19.7 | 9.67 |
| 46 | 0.460 | 4.33 | 13 |
| 39 | 0.428 | 14.3 | 20.3 |
| 43 | 0.417 | 12.7 | 7 |
| 29 | 0.309 | 23.3 | 24.3 |
| 44 | 0.292 | 4 | 9.67 |
| 45 | 0.211 | 6.67 | 9 |
| 11 | 0.039 | 0 | 0.667 |
| 4 | 0.025 | 0.333 | 0.333 |
| 31 | 0.000 | 0 | 0 |
| 42 | 0.000 | 0 | 0 |
| 40 | 0.000 | 0 | 0 |
| 38 | 0.000 | 0 | 0 |
| 19 | 0.000 | 0 | 0 |
| 35 | 0.000 | 0 | 0 |
| **Thallus vs. seawater** | | | |
| **OTU** | **Contrib. %** | **Mean thallus** | **Mean seawater** |
| 11 | 15.310 | 0 | 637 |
| 4 | 15.210 | 0.333 | 627 |
| 1 | 14.860 | 630 | 26.3 |
| 15 | 5.547 | 237 | 6.33 |
| 12 | 3.460 | 148 | 3.67 |
| 8 | 3.376 | 157 | 19.3 |
| 35 | 2.962 | 0 | 123 |
| 6 | 2.690 | 111 | 0.667 |
| 19 | 2.605 | 0 | 108 |
| 13 | 2.565 | 113 | 10 |
| 10 | 2.157 | 94.3 | 6.67 |
| 2 | 2.080 | 115 | 32.7 |
| 34 | 1.954 | 85 | 3 |
| 7 | 1.726 | 76.7 | 5.67 |
| 23 | 1.632 | 68.7 | 1 |
| 5 | 1.618 | 73 | 6.67 |
| 31 | 1.475 | 0 | 60.3 |
| 3 | 1.428 | 64.3 | 4 |
| 24 | 1.340 | 57.3 | 1.67 |
| 38 | 1.133 | 0 | 47.7 |
| 22 | 1.100 | 51.3 | 6.67 |
| 42 | 1.093 | 0 | 45 |
| 36 | 1.049 | 45.7 | 2.67 |
| 27 | 0.968 | 41.3 | 3.33 |
| 41 | 0.944 | 40 | 2 |
| 25 | 0.883 | 35.3 | 0.667 |
| 16 | 0.874 | 48.7 | 14 |
| 18 | 0.741 | 30.3 | 0.667 |
| 14 | 0.725 | 36.3 | 6.33 |
| 30 | 0.679 | 28.7 | 1.33 |
| 32 | 0.674 | 28.7 | 1.67 |
| 28 | 0.651 | 28.3 | 1.67 |
| 40 | 0.538 | 0 | 22.3 |
| 29 | 0.503 | 23.3 | 3 |
| 20 | 0.475 | 39.3 | 21 |
| 37 | 0.441 | 19.7 | 1.67 |
| 17 | 0.436 | 41 | 26.3 |
| 21 | 0.403 | 16 | 21 |
| 26 | 0.396 | 30.7 | 18 |
| 9 | 0.356 | 21.3 | 8 |
| 39 | 0.326 | 14.3 | 1.33 |
| 43 | 0.228 | 12.7 | 3.67 |
| 45 | 0.158 | 6.67 | 0.333 |
| 33 | 0.135 | 6.67 | 1.33 |
| 46 | 0.052 | 4.33 | 2.33 |
| 44 | 0.040 | 4 | 4.33 |
| **Tip vs. whole seaweed** | | | |
| **OTU** | **Contrib. %** | **Mean tip** | **Mean whole seaweed** |
| 1 | 11.590 | 228 | 472 |
| 3 | 10.890 | 372 | 258 |
| 2 | 10.560 | 488 | 293 |
| 9 | 10.260 | 275 | 96 |
| 7 | 6.030 | 239 | 150 |
| 6 | 5.373 | 122 | 206 |
| 12 | 4.467 | 111 | 131 |
| 15 | 3.566 | 15.3 | 93 |
| 5 | 3.166 | 153 | 109 |
| 10 | 2.891 | 161 | 134 |
| 17 | 2.476 | 54 | 78.7 |
| 8 | 2.308 | 67 | 117 |
| 21 | 2.022 | 41.3 | 26.7 |
| 14 | 1.963 | 90.7 | 62 |
| 16 | 1.686 | 30.3 | 59.3 |
| 13 | 1.527 | 22.3 | 51.3 |
| 27 | 1.521 | 18.7 | 35 |
| 33 | 1.492 | 43.7 | 20.3 |
| 23 | 1.481 | 6 | 38 |
| 25 | 1.426 | 17 | 36 |
| 28 | 1.299 | 16.3 | 38.3 |
| 24 | 1.290 | 3.67 | 32 |
| 34 | 1.198 | 3.67 | 29.7 |
| 18 | 1.171 | 77 | 94.7 |
| 41 | 1.062 | 7 | 29.3 |
| 26 | 1.044 | 25.7 | 24.3 |
| 22 | 0.870 | 13.7 | 29.7 |
| 32 | 0.862 | 14.7 | 25 |
| 30 | 0.661 | 12.3 | 14.3 |
| 39 | 0.639 | 7.67 | 20.3 |
| 44 | 0.626 | 19.7 | 9.67 |
| 45 | 0.622 | 21.7 | 9 |
| 46 | 0.394 | 11.3 | 13 |
| 37 | 0.375 | 7.33 | 9.67 |
| 29 | 0.352 | 20 | 24.3 |
| 20 | 0.344 | 27.3 | 33.7 |
| 36 | 0.259 | 2.33 | 6.33 |
| 43 | 0.181 | 5.33 | 7 |
| 11 | 0.033 | 0 | 0.667 |
| 40 | 0.017 | 0.333 | 0 |
| 4 | 0.017 | 0 | 0.333 |
| 31 | 0.000 | 0 | 0 |
| 42 | 0.000 | 0 | 0 |
| 38 | 0.000 | 0 | 0 |
| 19 | 0.000 | 0 | 0 |
| 35 | 0.000 | 0 | 0 |
| **Tip vs. seawater** | | | |
| **OTU** | **Contrib. %** | **Mean tip** | **Mean seawater** |
| 11 | 14.740 | 0 | 637 |
| 4 | 14.640 | 0 | 627 |
| 2 | 10.950 | 488 | 32.7 |
| 3 | 8.266 | 372 | 4 |
| 9 | 6.131 | 275 | 8 |
| 7 | 5.261 | 239 | 5.67 |
| 1 | 4.587 | 228 | 26.3 |
| 10 | 3.549 | 161 | 6.67 |
| 5 | 3.358 | 153 | 6.67 |
| 35 | 2.850 | 0 | 123 |
| 6 | 2.718 | 122 | 0.667 |
| 12 | 2.523 | 111 | 3.67 |
| 19 | 2.507 | 0 | 108 |
| 14 | 2.061 | 90.7 | 6.33 |
| 18 | 1.776 | 77 | 0.667 |
| 31 | 1.419 | 0 | 60.3 |
| 8 | 1.158 | 67 | 19.3 |
| 38 | 1.091 | 0 | 47.7 |
| 42 | 1.052 | 0 | 45 |
| 33 | 0.957 | 43.7 | 1.33 |
| 21 | 0.945 | 41.3 | 21 |
| 17 | 0.709 | 54 | 26.3 |
| 16 | 0.574 | 30.3 | 14 |
| 45 | 0.517 | 21.7 | 0.333 |
| 40 | 0.509 | 0.333 | 22.3 |
| 26 | 0.482 | 25.7 | 18 |
| 27 | 0.462 | 18.7 | 3.33 |
| 29 | 0.420 | 20 | 3 |
| 25 | 0.413 | 17 | 0.667 |
| 44 | 0.367 | 19.7 | 4.33 |
| 28 | 0.334 | 16.3 | 1.67 |
| 32 | 0.333 | 14.7 | 1.67 |
| 13 | 0.329 | 22.3 | 10 |
| 30 | 0.284 | 12.3 | 1.33 |
| 22 | 0.252 | 13.7 | 6.67 |
| 15 | 0.228 | 15.3 | 6.33 |
| 46 | 0.226 | 11.3 | 2.33 |
| 20 | 0.225 | 27.3 | 21 |
| 39 | 0.164 | 7.67 | 1.33 |
| 37 | 0.156 | 7.33 | 1.67 |
| 41 | 0.152 | 7 | 2 |
| 23 | 0.120 | 6 | 1 |
| 43 | 0.082 | 5.33 | 3.67 |
| 24 | 0.049 | 3.67 | 1.67 |
| 36 | 0.045 | 2.33 | 2.67 |
| 34 | 0.038 | 3.67 | 3 |
| **Whole seaweed vs. seawater** | | | |
| **OTU** | **Contrib. %** | **Mean whole seaweed** | **Mean seawater** |
| 11 | 14.50 | 0.667 | 637 |
| 4 | 14.41 | 0.333 | 627 |
| 1 | 10.27 | 472 | 26.3 |
| 2 | 6.20 | 293 | 32.7 |
| 3 | 5.78 | 258 | 4 |
| 6 | 4.71 | 206 | 0.667 |
| 7 | 3.29 | 150 | 5.67 |
| 10 | 2.97 | 134 | 6.67 |
| 12 | 2.90 | 131 | 3.67 |
| 35 | 2.81 | 0 | 123 |
| 19 | 2.47 | 0 | 108 |
| 5 | 2.33 | 109 | 6.67 |
| 8 | 2.28 | 117 | 19.3 |
| 18 | 2.18 | 94.7 | 0.667 |
| 9 | 2.00 | 96 | 8 |
| 15 | 1.95 | 93 | 6.33 |
| 31 | 1.40 | 0 | 60.3 |
| 17 | 1.33 | 78.7 | 26.3 |
| 14 | 1.30 | 62 | 6.33 |
| 38 | 1.08 | 0 | 47.7 |
| 16 | 1.07 | 59.3 | 14 |
| 42 | 1.04 | 0 | 45 |
| 13 | 1.01 | 51.3 | 10 |
| 25 | 0.86 | 36 | 0.667 |
| 23 | 0.85 | 38 | 1 |
| 28 | 0.81 | 38.3 | 1.67 |
| 27 | 0.77 | 35 | 3.33 |
| 24 | 0.68 | 32 | 1.67 |
| 41 | 0.66 | 29.3 | 2 |
| 34 | 0.61 | 29.7 | 3 |
| 21 | 0.59 | 26.7 | 21 |
| 32 | 0.56 | 25 | 1.67 |
| 22 | 0.55 | 29.7 | 6.67 |
| 40 | 0.51 | 0 | 22.3 |
| 29 | 0.50 | 24.3 | 3 |
| 39 | 0.45 | 20.3 | 1.33 |
| 33 | 0.43 | 20.3 | 1.33 |
| 30 | 0.32 | 14.3 | 1.33 |
| 20 | 0.32 | 33.7 | 21 |
| 26 | 0.28 | 24.3 | 18 |
| 46 | 0.24 | 13 | 2.33 |
| 45 | 0.20 | 9 | 0.333 |
| 37 | 0.19 | 9.67 | 1.67 |
| 44 | 0.13 | 9.67 | 4.33 |
| 36 | 0.13 | 6.33 | 2.67 |
| 43 | 0.09 | 7 | 3.67 |

**Table S6.** Results of SIMPER analysis to detect the bacterial classes contributing most to the differences observed between the different samples. SIMPER analysis was performed on the basis of the Bray-Curtis similarity index.

| **Thallus vs. tip** | | | |
| --- | --- | --- | --- |
| **Class** | **Contrib. %** | **Mean thallus** | **Mean tip** |
| Planctomycetia | 30.63 | 91 | 395 |
| Alphaproteobacteria | 16.4 | 2.30E+03 | 2.34E+03 |
| Phycisphaerae | 12.54 | 146 | 20 |
| uncl. Cyanobacteria | 12.04 | 312 | 317 |
| uncl. Bacteria | 6.236 | 190 | 152 |
| Acidimicrobiia | 5.594 | 78.7 | 39.3 |
| Deltaproteobacteria | 3.833 | 70 | 41.7 |
| Verrucomicrobiae | 2.295 | 7 | 30 |
| Deinococci | 1.978 | 30.7 | 12.3 |
| Saprospiria | 1.708 | 25 | 31 |
| uncl. Proteobacteria | 1.523 | 55 | 58 |
| Blastocatellia | 1.409 | 14 | 0 |
| Flavobacteriia | 1.279 | 26.3 | 17 |
| uncl. Actinobacteria | 0.7607 | 14.7 | 8.67 |
| Caldilineae | 0.4969 | 2.67 | 4.67 |
| Actinobacteria | 0.469 | 7 | 3 |
| uncl. Candidatus Melainabacteria | 0.4321 | 4.33 | 0 |
| Gammaproteobacteria | 0.1349 | 2 | 0.667 |
| Oligoflexia | 0.1032 | 0 | 1 |
| Cytophagia | 0.1003 | 0 | 1 |
| Epsilonproteobacteria | 0.04472 | 0.333 | 0.333 |
| Fusobacteriia | 0 | 0 | 0 |
| Clostridia | 0 | 0 | 0 |
| **Thallus vs. whole seaweed** | | | |
| **Class** | **Contrib. %** | **Mean thallus** | **Mean whole seaweed** |
| Planctomycetia | 23.35 | 91 | 291 |
| Alphaproteobacteria | 23.22 | 2.30E+03 | 2.24E+03 |
| uncl. Cyanobacteria | 19.16 | 312 | 450 |
| Phycisphaerae | 10.27 | 146 | 52.7 |
| uncl. Bacteria | 7.794 | 190 | 151 |
| Acidimicrobiia | 4.419 | 78.7 | 81.7 |
| Deinococci | 1.856 | 30.7 | 15.3 |
| uncl. Proteobacteria | 1.853 | 55 | 59.7 |
| Deltaproteobacteria | 1.631 | 70 | 64.7 |
| Blastocatellia | 1.555 | 14 | 0 |
| Flavobacteriia | 1.207 | 26.3 | 25.3 |
| Verrucomicrobiae | 1.07 | 7 | 17 |
| uncl. Actinobacteria | 0.623 | 14.7 | 11.7 |
| Saprospiria | 0.4925 | 25 | 24.7 |
| uncl. Candidatus Melainabacteria | 0.4165 | 4.33 | 1.67 |
| Actinobacteria | 0.395 | 7 | 5 |
| Oligoflexia | 0.334 | 0 | 3 |
| Caldilineae | 0.1721 | 2.67 | 1.33 |
| Gammaproteobacteria | 0.1334 | 2 | 1 |
| Epsilonproteobacteria | 0.04935 | 0.333 | 0.333 |
| Cytophagia | 0 | 0 | 0 |
| Fusobacteriia | 0 | 0 | 0 |
| Clostridia | 0 | 0 | 0 |
| **Thallus vs. seawater** | | | |
| **Class** | **Contrib. %** | **Mean thallus** | **Mean seawater** |
| Alphaproteobacteria | 32.92 | 2.30E+03 | 2.20E+03 |
| uncl. Cyanobacteria | 13.16 | 312 | 144 |
| Phycisphaerae | 10.46 | 146 | 5.67 |
| uncl. Bacteria | 9.621 | 190 | 64.3 |
| Planctomycetia | 5.602 | 91 | 15.7 |
| Acidimicrobiia | 5.532 | 78.7 | 5.33 |
| Deltaproteobacteria | 5.01 | 70 | 3.33 |
| Actinobacteria | 3.529 | 7 | 56.3 |
| uncl. Proteobacteria | 2.805 | 55 | 18.7 |
| Epsilonproteobacteria | 2.585 | 0.333 | 35.3 |
| Deinococci | 2.13 | 30.7 | 2.33 |
| Saprospiria | 1.689 | 25 | 2.67 |
| Flavobacteriia | 1.101 | 26.3 | 36 |
| uncl. Actinobacteria | 1.054 | 14.7 | 0.667 |
| Blastocatellia | 1.036 | 14 | 0.667 |
| Verrucomicrobiae | 0.4043 | 7 | 1.67 |
| Fusobacteriia | 0.3412 | 0 | 4.67 |
| Caldilineae | 0.3411 | 2.67 | 7.33 |
| uncl. Candidatus Melainabacteria | 0.3218 | 4.33 | 0 |
| Gammaproteobacteria | 0.1437 | 2 | 4 |
| Cytophagia | 0.1212 | 0 | 1.67 |
| Clostridia | 0.09787 | 0 | 1.33 |
| Oligoflexia | 0 | 0 | 0 |
| **Tip vs. whole seaweed** | | | |
| **Class** | **Contrib. %** | **Mean tip** | **Mean whole seaweed** |
| Planctomycetia | 26.41 | 395 | 291 |
| Alphaproteobacteria | 23.96 | 2.34E+03 | 2.24E+03 |
| uncl. Cyanobacteria | 17.6 | 317 | 450 |
| Acidimicrobiia | 6.993 | 39.3 | 81.7 |
| uncl. Bacteria | 6.42 | 152 | 151 |
| Deltaproteobacteria | 4.136 | 41.7 | 64.7 |
| Phycisphaerae | 3.965 | 20 | 52.7 |
| Saprospiria | 1.951 | 31 | 24.7 |
| Verrucomicrobiae | 1.821 | 30 | 17 |
| Deinococci | 1.62 | 12.3 | 15.3 |
| uncl. Proteobacteria | 1.453 | 58 | 59.7 |
| Flavobacteriia | 1.448 | 17 | 25.3 |
| uncl. Actinobacteria | 0.5995 | 8.67 | 11.7 |
| Caldilineae | 0.5162 | 4.67 | 1.33 |
| Actinobacteria | 0.3586 | 3 | 5 |
| Oligoflexia | 0.3064 | 1 | 3 |
| uncl. Candidatus Melainabacteria | 0.1916 | 0 | 1.67 |
| Cytophagia | 0.1138 | 1 | 0 |
| Gammaproteobacteria | 0.08931 | 0.667 | 1 |
| Epsilonproteobacteria | 0.05131 | 0.333 | 0.333 |
| Fusobacteriia | 0 | 0 | 0 |
| Clostridia | 0 | 0 | 0 |
| Blastocatellia | 0 | 0 | 0 |
| **Tip vs. seawater** | | | |
| **Class** | **Contrib. %** | **Mean tip** | **Mean seawater** |
| Alphaproteobacteria | 31.08 | 2.34E+03 | 2.20E+03 |
| Planctomycetia | 26.51 | 395 | 15.7 |
| uncl. Cyanobacteria | 12.3 | 317 | 144 |
| uncl. Bacteria | 6.401 | 152 | 64.3 |
| Actinobacteria | 3.652 | 3 | 56.3 |
| uncl. Proteobacteria | 2.88 | 58 | 18.7 |
| Deltaproteobacteria | 2.787 | 41.7 | 3.33 |
| Acidimicrobiia | 2.502 | 39.3 | 5.33 |
| Epsilonproteobacteria | 2.465 | 0.333 | 35.3 |
| Saprospiria | 2.062 | 31 | 2.67 |
| Verrucomicrobiae | 2.016 | 30 | 1.67 |
| Flavobacteriia | 1.427 | 17 | 36 |
| Phycisphaerae | 1.291 | 20 | 5.67 |
| Deinococci | 0.7528 | 12.3 | 2.33 |
| uncl. Actinobacteria | 0.5755 | 8.67 | 0.667 |
| Caldilineae | 0.4561 | 4.67 | 7.33 |
| Fusobacteriia | 0.3255 | 0 | 4.67 |
| Gammaproteobacteria | 0.2332 | 0.667 | 4 |
| Clostridia | 0.09338 | 0 | 1.33 |
| Oligoflexia | 0.07364 | 1 | 0 |
| Cytophagia | 0.06318 | 1 | 1.67 |
| Blastocatellia | 0.0446 | 0 | 0.667 |
| uncl. Candidatus Melainabacteria | 0 | 0 | 0 |
| **Water vs. whole seaweed** | | | |
| **Class** | **Contrib. %** | **Mean water** | **Mean whole seaweed** |
| Alphaproteobacteria | 29.77 | 2.20E+03 | 2.24E+03 |
| uncl. Cyanobacteria | 19.92 | 144 | 450 |
| Planctomycetia | 17.83 | 15.7 | 291 |
| uncl. Bacteria | 5.642 | 64.3 | 151 |
| Acidimicrobiia | 5.091 | 5.33 | 81.7 |
| Deltaproteobacteria | 3.972 | 3.33 | 64.7 |
| Actinobacteria | 3.207 | 56.3 | 5 |
| Phycisphaerae | 3.082 | 5.67 | 52.7 |
| uncl. Proteobacteria | 2.743 | 18.7 | 59.7 |
| Epsilonproteobacteria | 2.254 | 35.3 | 0.333 |
| Saprospiria | 1.454 | 2.67 | 24.7 |
| Flavobacteriia | 1.046 | 36 | 25.3 |
| Verrucomicrobiae | 0.9799 | 1.67 | 17 |
| Deinococci | 0.8692 | 2.33 | 15.3 |
| uncl. Actinobacteria | 0.7282 | 0.667 | 11.7 |
| Caldilineae | 0.3838 | 7.33 | 1.33 |
| Fusobacteriia | 0.2977 | 4.67 | 0 |
| Oligoflexia | 0.1969 | 0 | 3 |
| Gammaproteobacteria | 0.1886 | 4 | 1 |
| uncl. Candidatus Melainabacteria | 0.1099 | 0 | 1.67 |
| Cytophagia | 0.1058 | 1.67 | 0 |
| Clostridia | 0.08539 | 1.33 | 0 |
| Blastocatellia | 0.04079 | 0.667 | 0 |

**Table S7.** Putative identification, presence (+), origin and reported bioactivities of metabolites annotated in various *F. vesiculosus* extracts by LC-MS/MS and algal surface imprints by DESI-IMS.FVAI: surface extraction by dipping method; FVAII: surface extraction by C18 method; FVBI: surface-free extract after solvent dipping; FVBII: surface-free extract after C18 adsorption; FVC: whole algal extract of *F. vesiculosus*. The (putative) annotation of all metabolites was based on comparison of both parent ion *m/z* and fragment ions in databases. The only exceptions were compounds **37** and **49** for which no fragmentation was obtained, thus their putative identification was only based on parent ion.

^1^Δ: the relative *m/z* difference between the experimental and the calculated *m/z* for the [M+H]^+^ or the [M+Na]^+^ ion, as stated in the ion type column.^2^: Confidence level of the putative identification (based on Summer, et al.^1^).

| **N°** | **Rt (min)** | ***m/z*** | **Ion type** | **Calcd. *m/z* ([M+H]^+^ or [M+Na]^+^, etc.)** | **Putative Molecular Formula (M)** | **Δ ppm^1^** | **Fragment ions observed**  **(MS/MS)** | **Putative identification** | **Biological origin / Source** | **Chemical Class / Family** | **Biological Activity** | **Confidence Level^2^** | **FVAI** | **FVAII** | **FVBI** | **FVBII** | **FVC** | **DESI-IMS** | **References** |
| --- | --- | --- | --- | --- | --- | --- | --- | --- | --- | --- | --- | --- | --- | --- | --- | --- | --- | --- | --- |
| **1** | 0.45 | 205.069 | [M+Na]^+^ | 205.069 | C_6_H_14_O_6_ | 0 | 184.07; 166.05; 84.08 | Mannitol | *Macrocystis pyrifera*; *Dictyota coriacea* / Seaweed | Sugar-Polyol | Osmoregulation, storage, regeneration of reducing power, radical scavenger of ROS, antioxidant | 2 | **+** | **+** | **+** | **+** | **+** | **+** | 2–4 |
| **2** | 0.98 | 499.087 | [M+H]^+^ | 499.088 | C_24_H_18_O_12_ | 2.0 | 481.07; 463.07; 435.07; 413.08; 411.06; 395.08; 393.05; 355.04; 337.03; 315.09; 287.06; 271.02; 139.04 | Fucodiphlorethol (A, D, F); Difucophlorethol A; Tetrafucol (A, B); Tetraphlorethol (A) | *Fucus vesiculosus*; *Cystoseira baccata*; *C. retroflexa*; *Himanthalia elongata*; *Alaria marginata*; *Laminaria ochroleuca* / Seaweed | Phlorotannin | Antimicrobial, antioxidant, anticancer, UV protection, anti-coagulant and many other biological activities | 2 | **-** | **-** | **+** | **+** | **+** |  | 5–7 |
| **3** | 2.33 | 197.118 | [M+H]^+^ | 197.118 | C_11_H_16_O_3_ | 0 | 179.11; 161.10; 158.00; 135.12; 133.10; 129.54; 116.97; 107.08; 93.07 | Loliolide; Epiloliolide | *Sargassum ringgoldianum* subsp. *coreanum*; *Undaria pinnatifida*; *Padina tetrastromatica*; *Cystophora moniliformis*; *Sargassum crassifolium*; *S. thunbergii*; *Stokeyia indica*; *Dictyota dichotoma* / Seaweed | Monoterpene lactone | Anti-cancer, antibacterial, antifungal, antioxidant, | 2 | **+** | **-** | **-** | **-** | **-** |  | 8,9 |
| **4** | 4.04 | 242.139 | [M+H]^+^ | 242.139 | C_12_H_19_NO_4_ | 0 | 224.13; 165.05; 97.97; 93.07; 91.02; 77.04 | N-(3-oxooctanoyl) homoserine lactone | *Bosea massiliensis*; *Pseudomonas* sp.; *Lysobacter* sp.; *Pseudoalteromonas* sp. / Bacterium | Homoserine lactone | Quorum sensing signalling; defense responses; plant growth and development; biofilm formation | 2 | **-** | **+** | **+** | **-** | **-** | **+** | 10–16 |
| **5** | 4.80 | 258.124 | [M+H]^+^ | 258.124 | C_14_H_15_N_3_O_2_ | 0 | 236.15; 231.03; 224.13; 221.03; 165.05; 144.10; 98.92; 96.96; 93.07; 91.05; 77.04; 72.94 | Indolmycin | *Streptomyces griseus;*  *Pseudoalteromonas luteoviolacea* / Bacterium | L-tryptophan alkaloid | Activity against MRSA | 2 | **+** | **-** | **-** | **+** | **+** | **+** | 17,18 |
| **6** | 5.64 | 624.213 | [M+H]^+^ | 624.219 | C_31_H_33_N_3_O_11_ | 9.6 | 585.19; 583.19; 573.23; 567.22; 551.25; 393.31; 381.26; 343.13; 340.36; 337.11; 325.24; 285.24; 268.26 | Azinomycin B | *Streptomyces* sp. / Bacterium | Polyketide | Antitumor, antimicrobial against Gram-positive & Gram-negative bacteria | 2 | **+** | **-** | **+** | **-** | **-** | **+** | 19–21 |
| **7** | 5.98 | 787.460 | [M+2H]^2+^ | - | C_77_H_120_N_16_O_19_ | - | 714.42; 544.30; 445.24; 432.57; 360.21; 350.21;341.17; 242.11; 171.11; 152.10 | Emerimicin IV | *Emericellopsis* sp. / Fungus | Peptaibol | Bacteriostatic activity against Gram-positive organisms, incl. MRSA and vancomycin-resistant *Enterococcus faecalis* | 3 | **+** | **+** | **-** | **-** | **-** |  | 22,23 |
| **8** | 6.03 | 564.305 | [M+H]^+^ | - | - | - | 494.35; 446.35; 394.11; 378.14; 359.24; 343.13; 337.11; 270.31; 255.23; 236.15; 184.07; 104.11; 86.09 | - | *-* | Phospholipid |  | 3 | **+** | **+** | **-** | **-** | **-** | **+** | 24 |
| **9** | 6.09 | 343.130 | [M+H]^+^ | 343.129 | C_18_H_18_N_2_O_5_ | 2.9 | 300.29; 255.23; 236.15; 184.07; 104.11 | Pestalamide B | *Pestalotiopsis theae* / Fungus | Alkaloid amide | Antifungal (against *Aspergillus fumigalus*)  Antiviral (HIV) | 2 | **-** | **+** | **-** | **-** | **+** | **+** | 25 |
| **10** | 6.12 | 617.420 | [M+H]^+^ | - | - | - | 496.36; 446.35; 236.15 | DGTSA* | Chlorophyceae (Green algae, e.g. *Chlamydomonas reinhardtii*); Chrysophyceae (golden/golden-brown algae); Ochrophyta (e.g. *Nannochloropsis*); Phaeophyceae (brown algae; e.g. Fucales) / Seaweed | Betaine lipids |  | 3 | **-** | **-** | **-** | **+** | **+** |  | 24,26–28 |
| **11** | 6.24 | 645.522 | [M+H]^+^ | - | - | - | 446.35; 236.15; 144.10 | DGTSA* | Chlorophyceae (Green algae, e.g. *Chlamydomonas reinhardtii*); Chrysophyceae (golden/golden-brown algae); Ochrophyta (e.g. *Nannochloropsis*); Phaeophyceae (brown algae; e.g. Fucales) / Seaweed | Betaine lipids |  | 3 | **-** | **-** | **-** | **+** | **+** | **+** | 24,26–28 |
| **12** | 6.65 | 437.373 | [M+H]^+^ | - | - | - | 394.11; 371.16; 365.14; 359.24; 343.13; 337.11; 313.23; 236.15; 184.07; 125.00; 104.11; 86.09 | - | *-* | Phospholipid |  | 3 | **+** | **+** | **-** | **-** | **-** | **+** | 24 |
| **13** | 6.71 | 439.200 | [M+H]^+^ | 439.202 | C_28_H_26_N_2_O_3_ | 4.5 | 412.12; 408.13; 392.16; 376.18; 357.15; 351.13; 236.15; 222.97; 187.11 | Ochrindole C | *Aspergillus* sp. / Fungus | Bis-indolyl benzenoid | Activity against Gram-positive bacteria | 2 | **+** | **+** | **-** | **-** | **-** |  | 29 |
| **14** | 6.72 | 566.322 | [M+H]^+^ | - | - | - | 544.34; 472.36; 446.35; 395.24; 367.10; 352.13; 335.15; 236.15; 184.07; 104.11; 86.09 | - | *-* | Phospholipid |  | 3 | **+** | **+** | **-** | **-** | **-** |  | 24 |
| **15** | 6.73 | 542.336 | [M+H]^+^ | 542.333 | C_28_H_47_NO_9_ | 5.5 | 498.38; 367.10; 357.15; 351.13; 236.15; 144.10 | Novapikromycin | *Streptomyces* sp. / Bacterium | Macrolide | Activity against *Bacillus subtilis* | 2 | **+** | **-** | **-** | **+** | **-** |  | 30 |
| **16** | 6.76 | 454.127 | [M+H]^+^ | - | - | - | 409.13; 395.24; 367.10; 352.13; 335.15; 184.07; 155.14; 125.00; 104.11; 86.09 | - | *-* | Sulfoglycolipid |  | 3 | **+** | **+** | **-** | **-** | **-** |  | 31 |
| **17** | 6.78 | 568.340 | [M+H]^+^ | - | - | - | 550.33; 544.34; 409.13; 395.24; 367.10; 352.13; 335.15; 184.07; 125.00; 104.11; 86.09 | - | *-* | Sulfoglycolipid |  | 3 | **+** | **+** | **-** | **-** | **-** |  | 31 |
| **18** | 6.84 | 677.373 | [M+H]^+^ | - | - | - | 522.38; 498.38; 408.13; 392.16; 357.15; 351.13; 236.15; 144.10 | - | Chlorophyceae (Green algae, e.g. *Chlamydomonas reinhardtii*); Chrysophyceae (golden/golden-brown algae); Ochrophyta (e.g. *Nannochloropsis*); Phaeophyceae (brown algae; e.g. Fucales) / Seaweed | Betaine lipids |  | 3 | **+** | **-** | **+** | **+** | **+** |  | 24,26–28 |
| **19** | 7.22 | 518.321 | [M+H]^+^ | - | - | - | 459.35; 385.31; 371.16; 365.14; 313.23; 236.15; 184.07; 104.11 | - | *-* | Sulfoglycolipid |  | 3 | **+** | **+** | **-** | **-** | **-** |  | 31 |
| **20** | 7.23 | 420.346 | [M+H]^+^ | - | - | - | 385.31; 371.16; 365.14; 313.23; 184.07; 104.11 | - | *-* | Phospholipid |  | 3 | **+** | **+** | **-** | **-** | **-** |  | 24 |
| **21** | 7.23 | 447.198 | [M+H]^+^ | - | - | - | 422.14; 385.31; 371.16; 365.16; 313.23; 308.29; 184.07; 104.11 | - | *-* | Phospholipid |  | 3 | **+** | **+** | **-** | **-** | **-** |  | 24 |
| **22** | 7.23 | 465.170 | [M+H]^+^ | - | - | - | 422.14; 402.33; 385.31; 371.16; 365.14; 336.32; 313.23; 308.29; 184.07; 104.11 | - | *-* | Phospholipid |  | 3 | **+** | **+** | **-** | **-** | **-** |  | 24 |
| **23** | 7.23 | 475.329 | [M+H]^+^ | - | - | - | 447.25; 435.19; 385.31; 371.16; 365.14; 336.32; 311.26; 184.07; 104.11 | - | *-* | Phospholipid |  | 3 | **+** | **+** | **+** | **-** | **-** |  | 24 |
| **24** | 7.60 | 482.361 | [M+H]^+^ | - | - | - | 459.18; 443.20; 405.26; 361.23; 341.26; 332.33; 283.26; 235.17; 222.97; 201.05; 184.07; 165.05; 125.00; 104.11; 91.05 | - | *-* | Sulfoglycolipid |  | 3 | **+** | **+** | **-** | **-** | **-** |  | 31 |
| **25** | 7.61 | 788.355 | [M+H]^+^ | 788.349 | C_40_H_53_NO_15_ | 7.6 | 719.34; 616.44; 581.37; 422.14; 406.17; 381.12; 371.16; 365.14; 349.17 | Obelmycin F | *Streptomyces* sp. / Bacterium | Anthracycline | Weak cytotoxic avtivity | 2 | **+** | **+** | **-** | **-** | **-** | **+** | 32,33 |
| **26** | 7.62 | 687.334 | [M+H]^+^ | - | - | - | 616.44; 522.36; 467.36; 459.18; 443.20; 405.26; 361.23; 339.16; 333.14; 332.33; 283.26; 235.17; 201.05; 184.07; 104.11; 91.05 | - | *-* | Phospholipid |  | 3 | **+** | **+** | **-** | **-** | **-** |  | 24 |
| **27** | 7.71 – 8.49 | 641.429 | [M-H_2_O+H]^+^ | 641.421 | C_42_H_56_O_5_ | 12.4 | 614.50; 603.38; 581.40; 527.31; 411.27; 355.24; 209.13; 159.08; 149.09; 119.08; 109.10 | Dehydrated fucoxanthin  ([M-H_2_O]) | - | Carotenoid |  | 2 | **-** | **-** | **+** | **+** | **+** |  | 34 |
| **28** | 8.05 | 723.144 | [M+H]^+^ | - | - | - | 663.34; 561.40; 527.27; 500.39; 482.38; 236.15; 184.07; 144.10 | DGTSA* | Chlorophyceae (Green algae, e.g. *Chlamydomonas reinhardtii*); Chrysophyceae (golden/golden-brown algae); Ochrophyta (e.g. *Nannochloropsis*); Phaeophyceae (brown algae; e.g. Fucales)/ Seaweed | Betaine lipids |  | 3 | **+** | **-** | **-** | **-** | **-** |  | 24,26–28 |
| **29** | 8.18 | 455.314 | [M+H]^+^ | 455.312 | C_24_H_42_N_2_O_6_ | 4.39 | 326.38; 221.15; 186.22; 149.09 | Acremolide D | *Acremonium* sp. / Fungus | lipodepsipeptide | No biological activity known | 2 | **+** | **+** | **-** | **+** | **+** |  | 35,36 |
| **30** | 8.39 | 568.663 | [M+H]^+^ | 568.671 | C_33_H_36_N_4_O_5_ | 14.1 | 550.49; 529.82; 449.37; 427.39; 324.33; 184.07 | 4,5 secopyropheophorbide *a* | *Chlorella protothecoides /* Microalga | Tetrapyrrole |  | 2 | **+** | **+** | **-** | **-** | **-** |  | 37 |
| **31** | 8.59 | 639.409 | [M+H]^+^ | 639.411 | C_35_H_58_O_10_ | 3.13 | 611.36; 595.38; 375.25; 291.23; 247.21; 133.09; 89.06 | Filipin II | *Streptomyces* sp. / Bacterium | Polyene macrolide | Antifungal activity | 2 | **+** | **+** | **-** | **-** | **-** |  | 38,39 |
| **32** | 8.59 | 617.426 | [M+H]^+^ | 617.421 | C_40_H_56_O_5_ | 8.1 | 595.38; 375.25; 291.23; 247.21; 133.09; 89.06 | Fucoxanthinol | *Fucus vesiculosus;*  *Halocynthia roretzi /* Seaweed | Carotenoid | Antiproliferative activity; antioxidant and anti-inflammatory properties | 2 | **+** | **+** | **-** | **-** | **-** |  | 34,40,41 |
| **33** | 8.89 | 709.453 | [M+H]^+^ | 709.453 | C_39_H_64_O_11_ | 0 | 683.43; 672.51; 649.45; 639.41; 630.46; 595.38; 531.37; 443.30 399.27; 383.18; 375.25; 360.36; 242.25 | Bahamaolide A | *Streptomyces* sp. / Bacterium | Macrocyclic lactone | Antifungal activity (against *Aspergilus* sp., *Trichoderma* sp. and *Candida* sp.) | 2 | **+** | **-** | **-** | **-** | **-** |  | 42,43 |
| **34** | 9.04 | 665.425 | [M+H]^+^ | 665.426 | C_37_H_60_O_10_ | 0.01 | 629.40; 609.39; 607.38; 605.42; 565.37; 487.32; 399.27 | Mycoticin B | *Streptomyces* sp. / Bacterium | Poly 1,3 hydroxyated macrocyclic polyenelactone | Antifungal antibiotic | 2 | **+** | **+** | **-** | **-** | **-** |  | 44,45 |
| **35** | 9.17 | 469.211 | [M+H]^+^ | 469.213 | C_29_H_28_N_2_O_4_ | 4.26 | 429.32; 425.21; 409.21; 385.29; 341.27 | Ochrindole B | *Aspergillus* sp. / Fungus | Bis-indolyl benzenoid |  | 2 | **+** | **-** | **-** | **-** | **-** |  | 29 |
| **36** | 9.20 | 263.239 | [M+H]^+^ | 263.237 | C_18_H_30_O | 7.6 | 245.23; 207.03; 184.89; 177.17; 175.15; 163.15; 151.14; 149.13; 137.13; 123.12; 121.10; 109.10; 95.08 | Farnesylacetone | *Carcinus maenas*/ *Arthropod*  (Derivative of farnesylacetone isolated from Fucales, e.g. *Sargassum micracanthum* and *Cystophora moniliformis /* Seaweed | Sesquiterpenoid | Antimicrobial activity against human Gram-positive bacteria  (derivatives: cholinesterase inhibitory activities) | 2 | **-** | **-** | **-** | **-** | **+** | **+** | 46–48 |
| **37** | 9.84 | 256.267 | [M+H]^+^ | 256.264 | C_16_H_33_NO | 11.7 | - | Palmitamide | *Prymnesium parvum* / Microalga | Fatty acid amide | Cytotoxic and ichthytoxic activity | 2 | **+** | **-** | **-** | **-** | **-** | **+** | 49 |
| **38** | 10.34 | 235.131 | [M+H]^+^ | 235.133 | C_14_H_18_O_3_ | 8.5 | 95.08 | Dihydrosorbicillin | *Verticillium inlertextum*  / Fungus | Polyketide | Antimicrobial activity against Gram-positive bacteria (*Staphylococcus aureus* and *Bacillus subtilis*) | 2 | **-** | **+** | **+** | **-** | **-** | **+** | 50,51 |
| **39** | 10.45 | 672.437 | [M+H]^+^ | 672.434 | C_36_H_57_N_5_O_7_ | 4.4 | 648.46; 633.46; 612.49; 589.43; 557.42; 550.49; 491.26; 461.32; 429.19; 368.39; 270.28; 158.15; 71.08 | Scopularide A | *Scopulariopsis brevicaulis* / Fungus | Cyclodepsipeptide | Weak activity against Gram-positive and Gram-negative bacteria  Antitumor activity | 2 | **+** | **-** | **-** | **-** | **+** |  | 44 |
| **40** | 10.96 | 681.413 | [M+Na]^+^ | 681.413 | C_42_H_58_O_6_Na | 0 | 663.41; 621.39; 603.38; 527.31 | Fucoxanthin** | *Sargassum siliquastrum;*  *Fucus evanescens;*  *F. vesiculosus* / Seaweed | Carotenoid | Antioxidant, anti-inflammatory, anticancer, anti-obese, antidiabetic, antiangiogenic and antimalarial activities | 1 | **-** | **-** | **+** | **+** | **+** | **+** | 34 |
| **41** | 10.96 | 887.570 | [M+H]^+^ | 887.569 | C_55_H_74_N_4_O_6_ | 1.1 | 784.61; 765.46; 734.59; 696.58; 308.30 | 10S-hydroxypheophytin *a* | *Isochrysis galbana /* Microalga *Clerodendrum sp .* / Plant | Chlorophyll *a* derivative |  | 2 | **-** | **-** | **+** | **-** | **-** |  | 52–54 |
| **42** | 11.22 | 593.277 | [M+H]^+^ | 593.276 | C_35_H_36_N_4_O_5_ | 1.7 | 533.25; 471.36; 460.22; 427.34; 309.28; 280.26; 135.12; 97.09; 81.07 | Pheophorbide *a* | Saccharina japonica; *Capsosiphon fulvescens* / Seaweed  *Scutellaria barbata*;  *Arrabidaea chica* / Plant | Tetrapyrrole | Anti-proliferative activity, anti-inflammatory | 2 | **-** | **-** | **+** | **+** | **+** |  | 55–57 |
| **43** | 11.34 | 452.282 | [M+H]^+^ | 452.280 | C28H37NO4 | 4.4 | 421.23; 393.24; 369.31; 351.30; 115.08; 91.05 | Cytochalasin O | *Phomopsis* sp. / Fungus | Indole alkaloid | Antmicrobial activity against *B. subtilis* and *E. coli* | 2 | **+** | **-** | **-** | **+** | **-** |  | 58 |
| **44** | 11.84 | 656.555 | [M+H]^+^ | 656.546 | C_38_H_73_NO_7_ | 13.7 | 619.46; 575.47; 446.35; 428.34; 325.27; 236.15; 162.11; 144.10; 100.11 | DGTSA* (14:0 / 14:0) | Chlorophyceae (Green algae, e.g. *Chlamydomonas reinhardtii*); Chrysophyceae (golden/golden-brown algae); Ochrophyta (e.g. *Nannochloropsis*); Phaeophyceae (brown algae; e.g. Fucales) / Seaweed | Betaine lipids | Phosphate starvation; adaptation to low temperature | 2 | **-** | **-** | **+** | **+** | **+** | **+** | 24,26–28 |
| **45** | 11.85 | 657.946 | [M+H]^+^ | - | - | - | 619.46; 575.47; 446.35; 428.34; 325.27; 313.24; 236.15; 162.11; 144.10; 100.11 | DGTSA* | Chlorophyceae (Green algae, e.g. *Chlamydomonas reinhardtii*); Chrysophyceae (golden/golden-brown algae); Ochrophyta (e.g. *Nannochloropsis*); Phaeophyceae (brown algae; e.g. Fucales) / Seaweed | Betaine lipid | Phosphate starvation; adaptation to low temperature | 3 | **-** | **-** | **-** | **+** | **+** |  | 24,26–28 |
| **46** | 12.05 | 708.597 | [M+H]^+^ | - | - | - | 671.45; 627.50; 614.60; 498.38; 480.37; 446.35; 428.34; 307.31; 236.15; 200.13; 162.11; 144.10; 100.11 | DGTSA* | Chlorophyceae (Green algae, e.g. *Chlamydomonas reinhardtii*); Chrysophyceae (golden/golden-brown algae); Ochrophyta (e.g. *Nannochloropsis*); Phaeophyceae (brown algae; e.g. Fucales) / Seaweed | Betaine lipid | Phosphate starvation; adaptation to low temperature | 2 | **-** | **-** | **+** | **+** | **+** | **+** | 24,26–28 |
| **47** | 12.37 | 515.081 | [M+H]^+^ | 515.082 | C_24_H_18_O_13_ | 1.9 | 491.26; 485.38; 471.11; 441.35; 397.33; 312.33; 283.26; 184.08; 172.12; 128.14; 116.05 | Hydroxyfucodiphlorethol | *Sargassum spinuligerum,* *Cystophora torulosa /* Seaweed | Phlorotannin | Antimicrobial, antioxidant, anticancer, UV protection, anti-coagulant and other pharmacological activities | 2 | **+** | **+** | **-** | **-** | **-** |  | 7,59 |
| **48** | 12.48 | 715.608 | [M+H]^+^ | - | - | - | 661.64; 645.49; 629.51; 522.38; 500.39; 482.38; 446.35; 428.34; 308.29; 236.15; 200.13; 162.11; 100.11 | DGTSA* | Chlorophyceae (Green algae, e.g. *Chlamydomonas reinhardtii*); Chrysophyceae (golden/golden-brown algae); Ochrophyta (e.g. *Nannochloropsis*); Phaeophyceae (brown algae; e.g. Fucales) / Seaweed | Betaine lipid | Phosphate starvation; adaptation to low temperature | 3 | **-** | **-** | **-** | **+** | **+** |  | 24,26–28 |
| **49** | 12.50 | 395.364 | [M+H]^+^ | 395.364 | C_24_H_46_N_2_O_2_ | 0 | - | Stockerine | *Stockeyia indica* / Seaweed | Linear aminolipid | No biological activity reported | 2 | **-** | **+** | **+** | **-** | **-** |  | 60 |
| **50** | 13.07 | 871.578 | [M+H]^+^ | 871.574 | C_55_H_74_N_4_O_5_ | 4.6 | 593.28; 533.25 (minor: 856.55; 810.53; 755.71; 736.63; 690.37; 661.30; 640.65; 635.90; 609.03; 606.90; 519.24;485.55; 480.56; 478.57; 460.23; 448.11; 446.80; 325.83; 238.17; 235.03) | Pheophytin *a* | *Sargassum fulvellum; Enteromorpha (Ulva) prolifera* / Seaweed | Chlorophyll *a* derivative | Anti-inflammatory activity; antioxidant and free radical scavenging activities | 2 | **-** | **-** | **+** | **-** | **-** |  | 52,61,62 |

*DGTSA: 1,2-diacylglyceryl-3-O-4'-(N,N,N-trimethyl)-homoserine (abbreviated to DGTS) or 1,2-diacylglyceryl-3-O-2'-(hydroxymethyl)-(N,N,N-trimethyl)-β-alanine (abbreviated to DGTA).

** Identification based on commercial standard purchased.

**
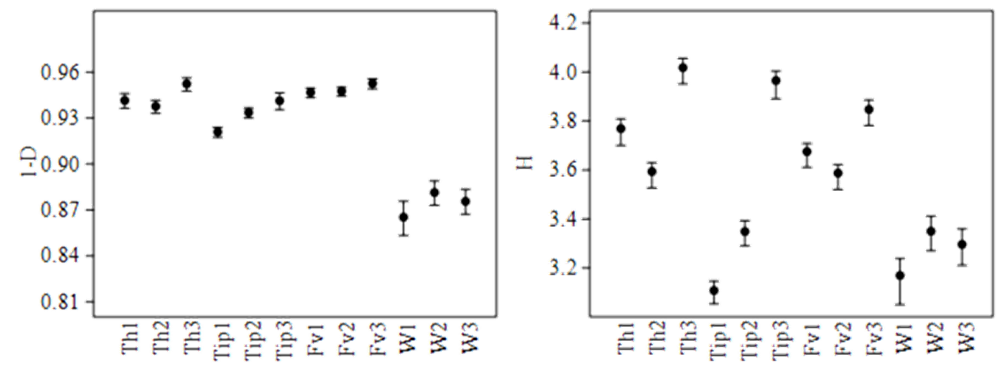
**

**Figure S1.** Simpson and Shannon diversity indices for all *F. vesiculosus* and seawater reference samples. Left: Simpson index ± standard error. Right: Shannon index ± standard error. Th1-3: Thallus replicates 1-3, Tip1-3: Tip replicates 1-3, Fv1-3: Whole seaweed replicates 1-3, W1-3: Seawater reference samples 1-3.


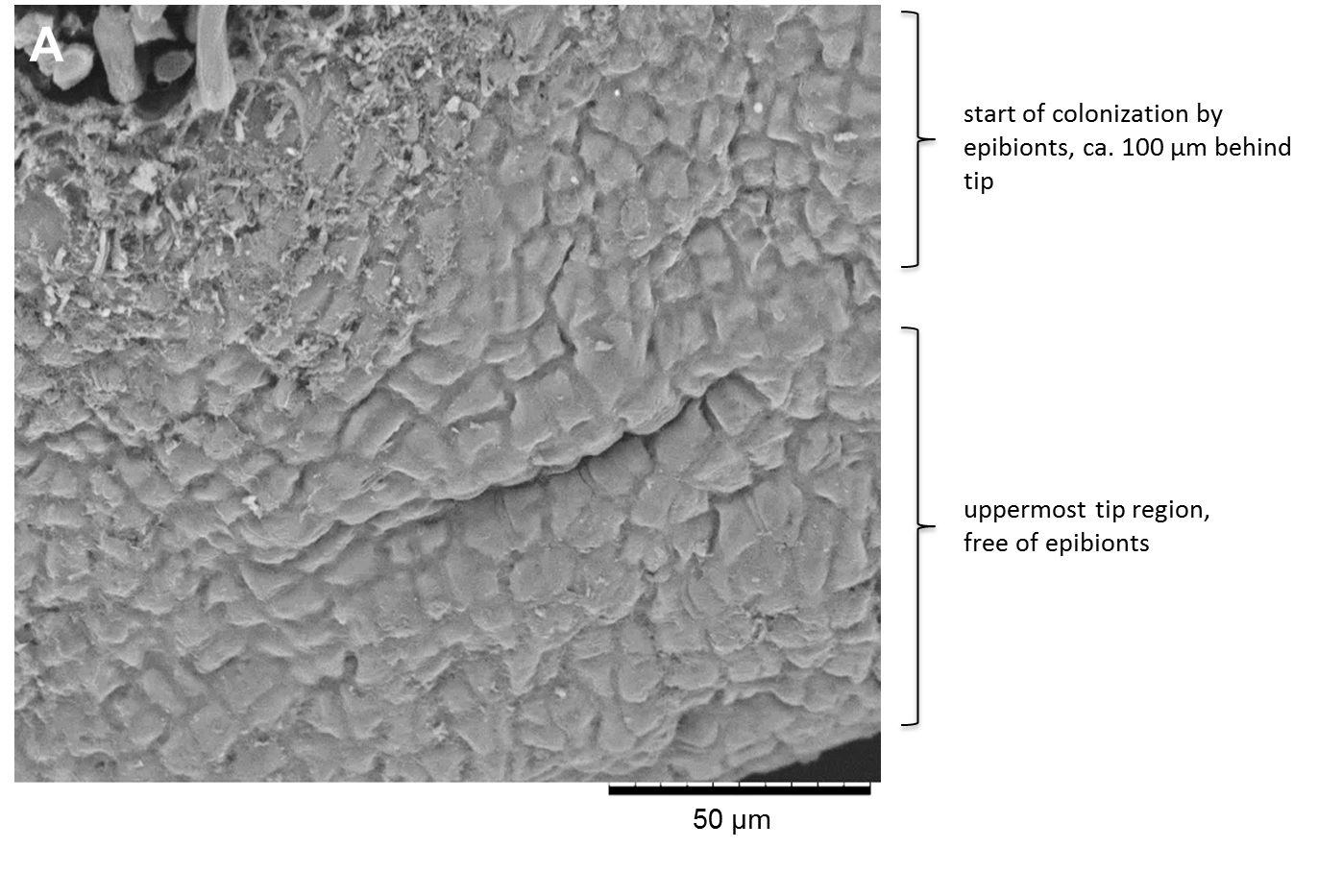


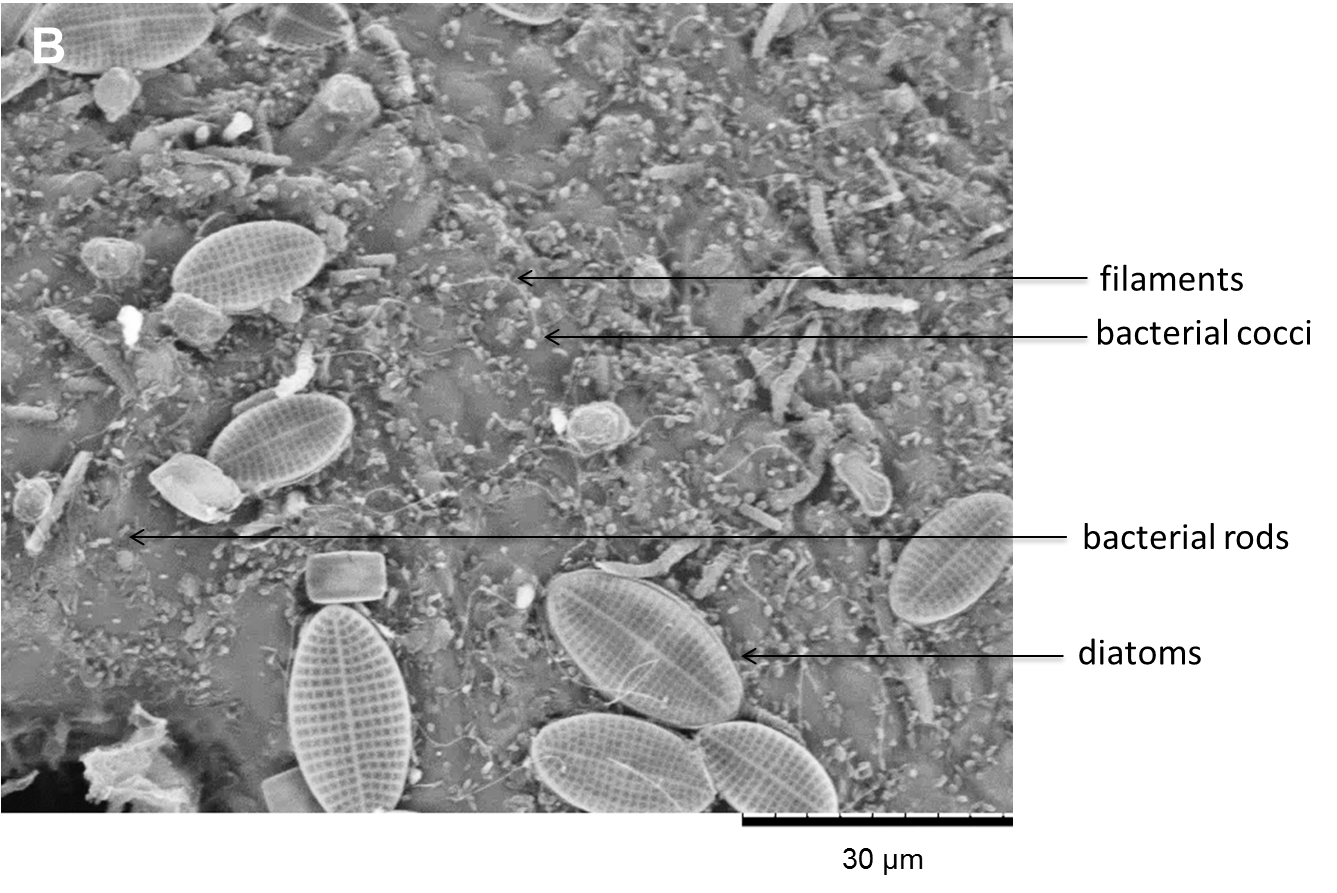


**Figure S2 A**. SEM images of uppermost tip region. The dense colonization by epibionts starts ca. 100 µm below the tip (magnification: 1200x). **B.** Epibionts on older thallus regions show a multitude of bacterial morphotypes, e.g. rods or cocci. Furthermore, diatoms and long filaments are found as epibionts on the algal surface (magnification: 2000x).

50 µm


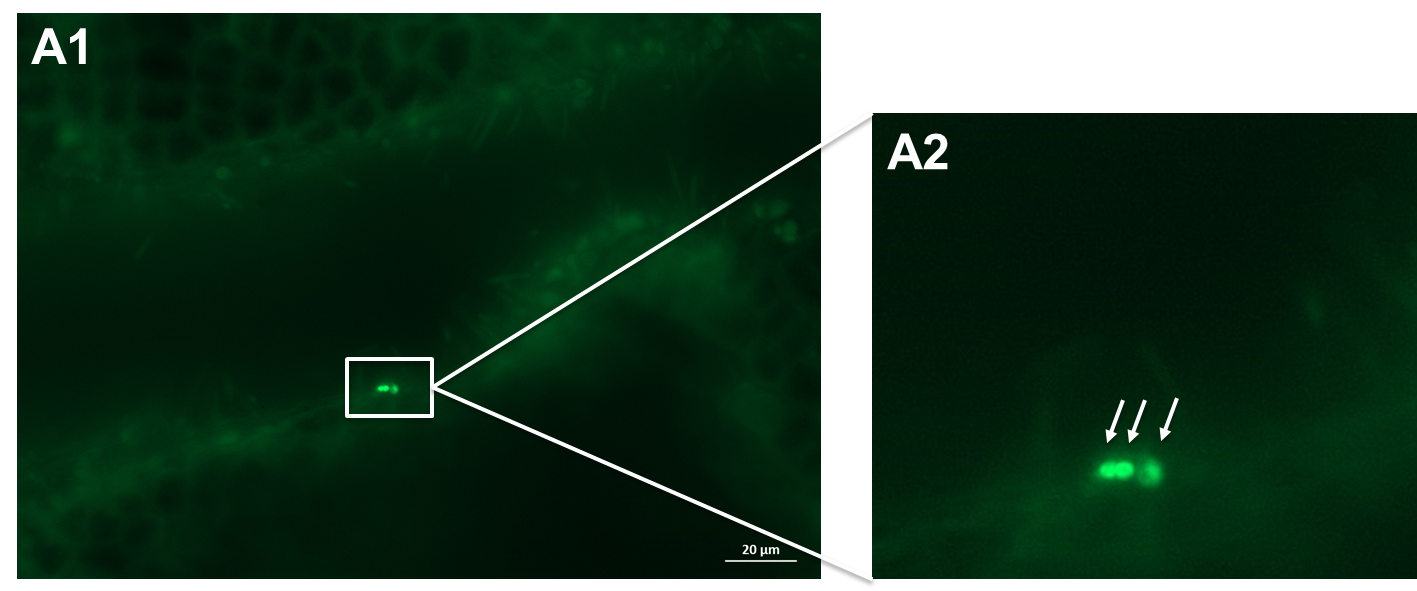


**
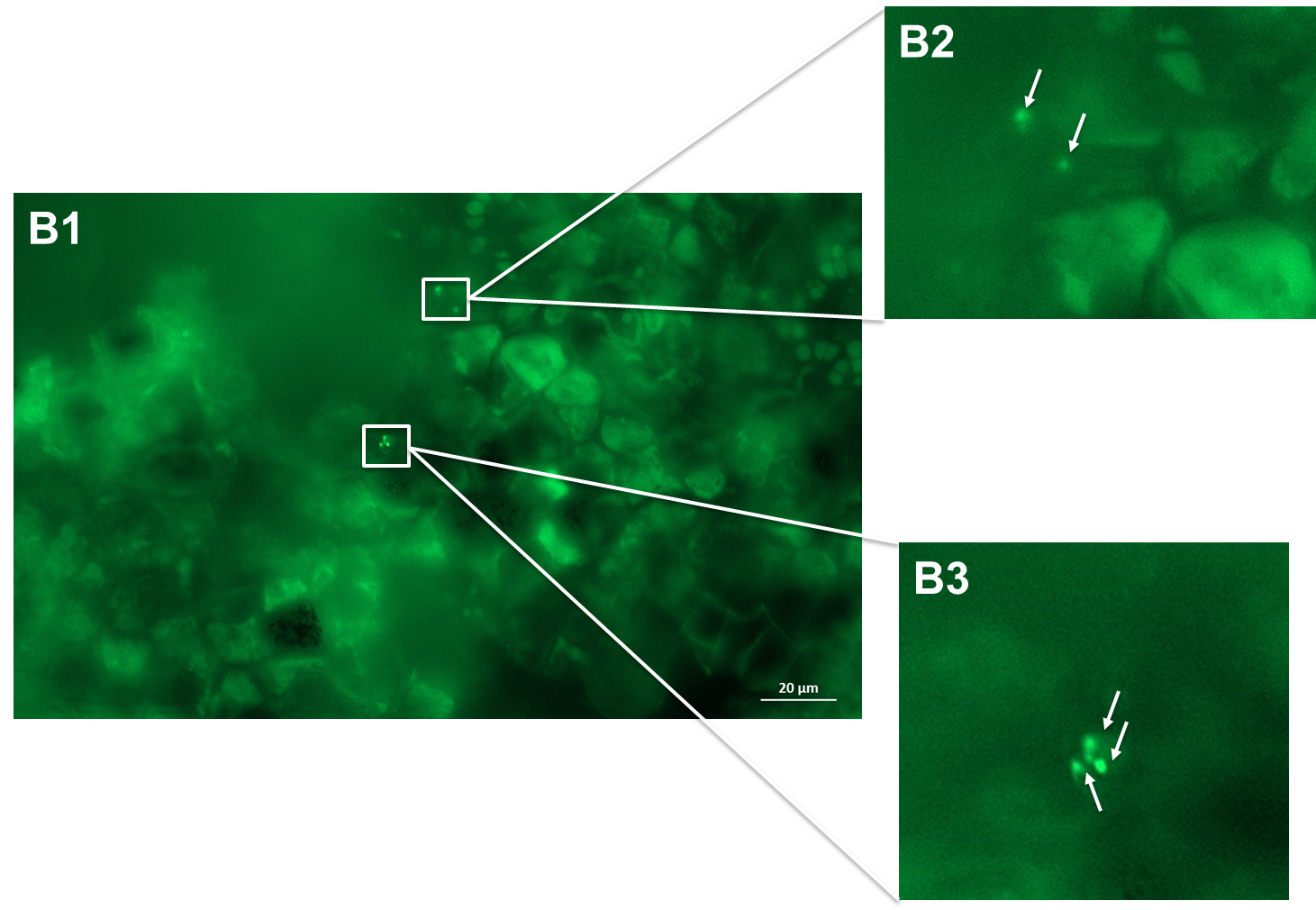
**

**
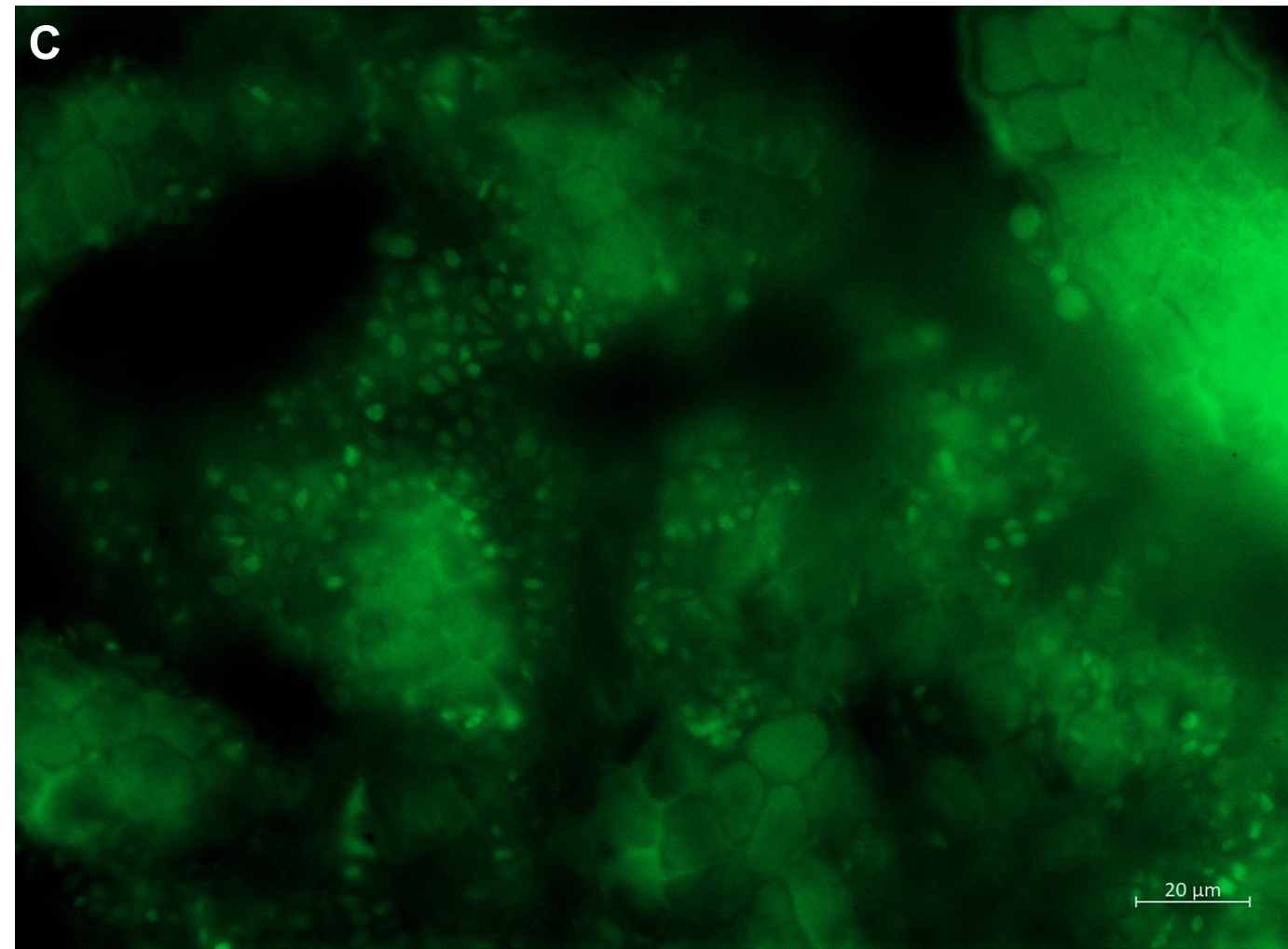
**

**Figure S3.** Epifluorescence microscopic images of *F. vesiculosus* surface using biotin-labelled probes for CARD-FISH targeting specific microbial groups. Images were acquired using the eGFP filter set (excitation 488 nm/emission 509 nm). Applied CARD-FISH probes targeted **A1**. Actinobacteria (probe HGC236, **A2.** close-up of A1). **B1**. Firmicutes (probe LGC254 A/B/C, **B2/3**. close-ups of B1). **C.** Alphaproteobacteria (probe ALF968).

**Detailed description of CARD-FISH.**

Preparation for CARD-FISH involved subsequent steps for permeabilization of microbial cells, inactivation of endogenous peroxidases, hybridization of the respective oligonucleotide probes, coupling of horseradish peroxidase to the oligonucleotide probes, catalyzed reporter deposition (CARD) of oligonucleotide probes coupled to horseradish peroxidase (HRP) and counter-staining by 4‘,6-diamidino-2-phenylindole (DAPI). For CARD-reaction TSA kit 22 (Thermo Fisher Scientific, Schwerte, Germany) was used. After embedding the algal pieces in 0.1% pre-filtered agarose, permeabilization of the microbial cells was realized by application of 10 mg/mL lysozyme solution for 60 min at 37°C followed by a washing step in ultra-purified water. The inactivation of endogenous peroxidases (30 min incubation, RT) took place by transferring the algal pieces to MeOH containing

0.15% H_2_O_2_ with subsequent washing steps in Milli-Q water and 96% EtOH, followed by air-drying. For probe hybridization, algal pieces were placed into 1.5 mL Eppendorf reaction tubes with 300 µL freshly prepared hybridization buffer (5 M NaCl, 1 M Tris-HCl (pH 8.0), blocking reagent from TSA kit, 20 µL of 20% (w/v) SDS, water and formamide containing the respective recommended concentration for hybridization ([http://probebase.csb.univie.ac.at](http://probebase.csb.univie.ac.at/)) for each probe, i.e. 20% formamide for ALF968, 35% for LGC354A/B/C, 10% for Eur1108, and 0% for HGC236) as well as 1 µL of the biotin-labelled probe (concentration 50 ng/µL) followed by an incubation at 46 °C for 2.5h. Amount of water and formamide in the hybridization buffer followed recommendations of the published CARD-FISH protocols (publicly available at https://www.arbsilva.de/fileadmin/graphics_fish/SILVA_FISH_protocols_card_101025_V2_2.pdf).

Samples were then transferred to sterile vials containing freshly prepared preheated washing buffer (0.5 M EDTA (pH 8), 1M Tris-HCl, 10% SDS, ultra-purified water, and 5 M NaCl depending on the formamide concentration in the hybridization buffer) and incubated at 48 °C for 10 min. Washing in 1x PBS and 2 x SSC for 15 min each, was followed by adding 100 µL of blocking reagent (incubated for 30 min at RT). Subsequently, HRP was coupled to the oligonucleotide probes using a 1:100 streptavidin-HRP/blocking reagent solution, at 20°C for 30 min, followed by washing in 1x PBS for 10 min. All subsequent steps were performed in the dark to avoid photo-bleaching of the dye. Samples were transferred to new Eppendorf reaction tubes containing 500 µL of amplification buffer (1x PBS, 0.15 % H_2_O_2_, 1 µL of the Alexa 488-labelled tyramide and incubated for 20 min followed by washing in 1x PBS for 10 min, each 2 min in MilliQ water and 96% EtOH and air-dried on a filter paper. DAPI counter staining was performed by adding 100 µL of DAPI solution (1 µg/mL) to each algal piece. Staining was carried out for 10 min at 20 °C, followed by a washing step in ultra-purified water for 5 min and EtOH (96%). After air-drying all samples were embedded for microscopy in 5 µL Citifluor anti-fading agent on microscopy slides and covered with a coverslide.


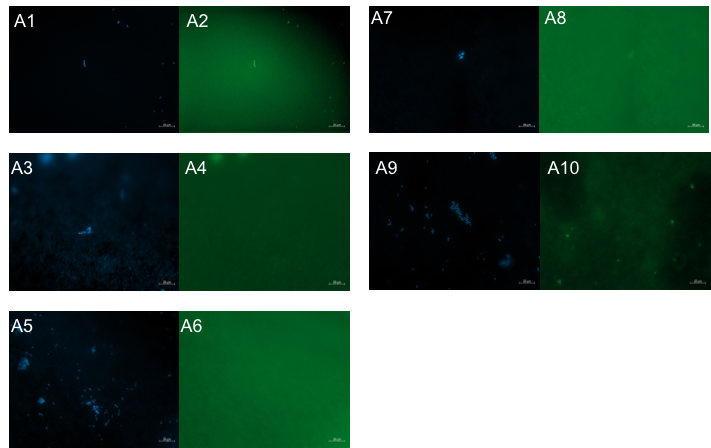


**Figure S4.** Epifluorescence microscopic images using biotin-labelled probes for CARD-FISH control experiments on pure cultures to test for probe specificity. A-D1,3,5,7,9 respectively, show images acquired using the DAPI filter set, whereas A-D 2,4,6,8,10 show images acquired using the EGFP filter set. A1-10: Control experiments for probe LGC354A,B,C; A1,2: *Bacillus subtilis* DSM 347 used as positive control, A3,4: *Streptomyces globisporus* DSM 41647 used as negative control, A5,6: *Gluconobacter oxydans* DSM7145, used as negative control, A7,8: *Saccharomyces cerevisiae* DSM70449 used as negative control (Note: rRNA was not stained. Light coloration from stain residue in cells), A9,10: *Penicllium* sp., used as negative control (Note: spores from pure culture were used for staining). B1-10: Control experiments for probe Eur1108; B1,2: *Penicllium* sp., used as positive control, B3,4: *Bacillus subtilis* DSM 347 used as negative control, B5,6: *Gluconobacter oxydans* DSM7145, used as negative control, B7,8: *Streptomyces globisporus* DSM 41647, used as negative control, B9,10: *Saccharomyces cerevisiae* DSM70449, used as negative control (Note: rRNA was not stained. Light coloration from stain residue in cells). C1-10: Control experiments for probe ALF968, C1,2: *Gluconobacter oxydans* DSM7145, used as positive control, C3,4: *Bacillus subtilis* DSM 347, used as negative control, C5,6: *Streptomyces globisporus* DSM 41647, used as negative control, C7,8: *Saccharomyces cerevisiae* DSM70449, used as negative control, C9,10: *Penicllium* sp., used as negative control. D1-10: control experiments for probe HGC236; D1,2) *Streptomyces globisporus* DSM 41647, used as positive control, D3,4: *Bacillus subtilis* DSM 347, used as negative control, D5,6: *Gluconobacter oxydans* DSM7145, used as negative control, D7,8: *Penicllium* sp., used as negative control (Note: spores from pure culture were used for staining), D9,10: *Saccharomyces cerevisiae* DSM70449, used as negative control (Note: rRNA was not stained. Light coloration from stain residue in cells).


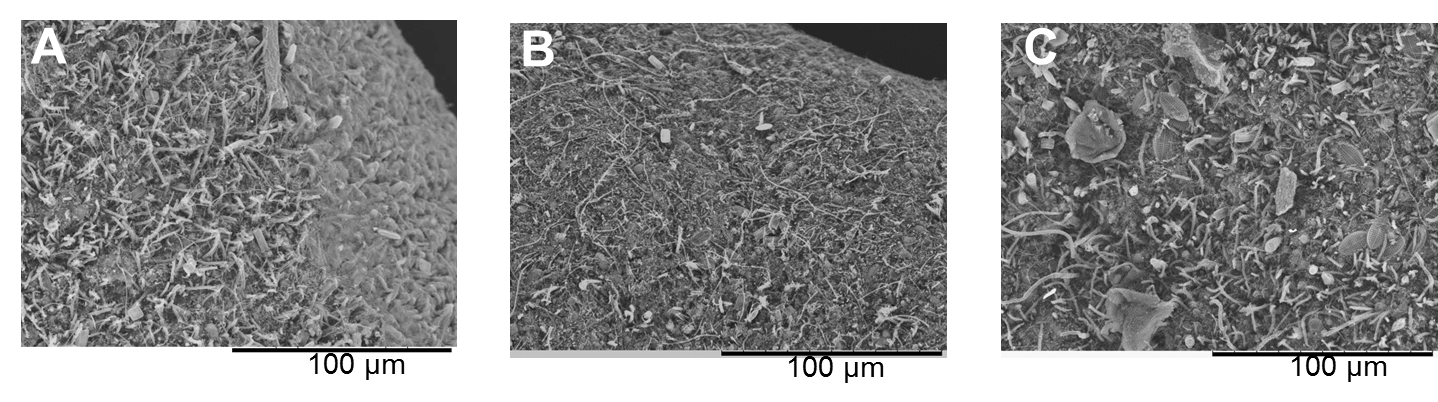


**Figure S5.** SEM images of the surface of *F. vesiculosus* before and after surface extractions*.* **A.** Untreated, before any extraction was performed. **B.** After surface extraction by dipping in *n*-hexane:MeOH (1:1, v/v), **C.** After extraction with C18 material. Magnification: 1000x.

FVAI

FVAII

FVBI

FVBII

FVC

**Figure S6.** Base peak chromatograms (acquired by an UPLC-QTOF-MS/MS system in positive mode) of the *F. vesiculosus* extracts. FVAI: Surface solvent extract. FVAII: Surface C18 extract. FVBI: Surface-free extract after solvent dipping. FVBII: Surface-free extract after C18 adsorption. FVC: Whole algal extract.

**Figure S7.** Chemical structures of metabolites annotated at chemical name level in the *F. vesiculosus* extracts. Numbers correspond to putatively annotated known compounds, which are reported in Table S7. Compound **27** that corresponds to the dehydrated form of fucoxanthin (**40**) (loss of a H_2_O molecule) is not represented in this figure. All putative hits for compounds **2** and **3**, reported in Table S7, are displayed by **2-1** to **2-6** and **3-1** and **3-2** respectively.

**Figure S7. (continued)**

**Figure S7. (continued)**


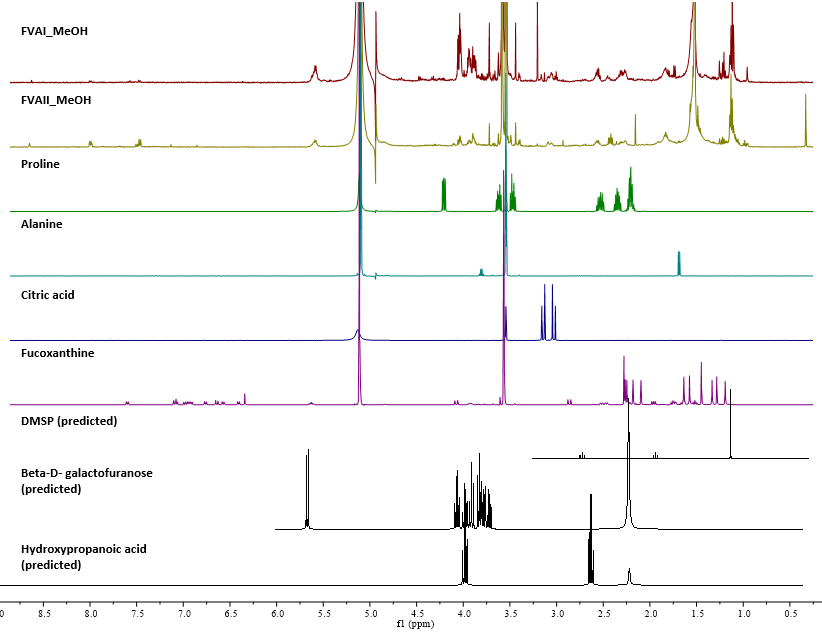


**A.**

**B.**


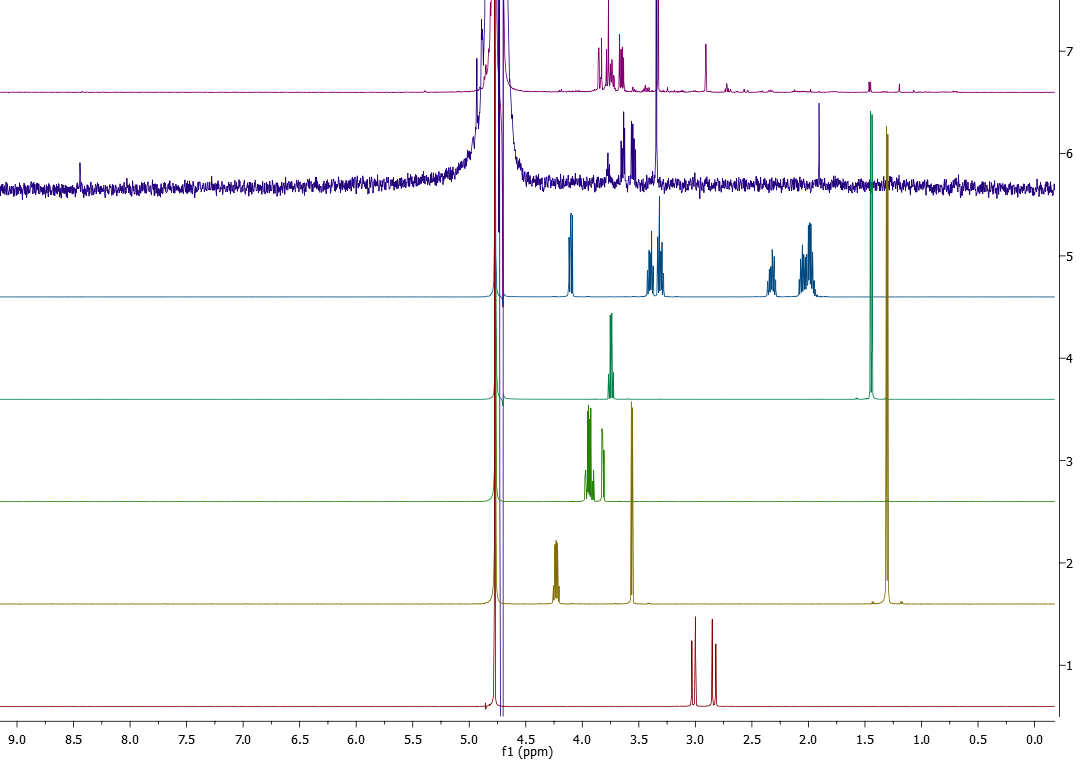


**FVAII_H_2_O**

**FVAI_H_2_O**

**Proline**

**Alanine**

**DMSP (predicted)**

**Citric acid**

**Beta-D- galactofuranose (predicted)**

**Hydroxypropanoic acid (predicted)**

**Serine**

**Threonine**

**Figure S8.** Comparison of the ^1^H-NMR spectra of the surface extracts FVAI and FVAII with those of the previously reported *F. vesiculosus* surface metabolites. Some compounds were commercially available (alanine, proline, serine, threonine, citric acid, fucoxanthin) while the ^1^H NMR spectra of some DMSP, hydroxypropanoic acid, β-D-galactofuranose were predicted in the respective NMR solvent. **A.** MeOH-soluble portion of the surface extract in MeOD and **B.** H_2_O-soluble portion of the surface extract in D_2_O.


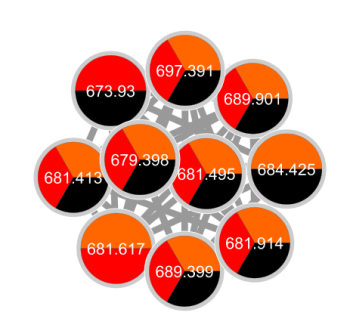


Fucoxanthin (**40**)

*m/z* [M+Na]^+^681.413

(*t_R_*10.96 min)


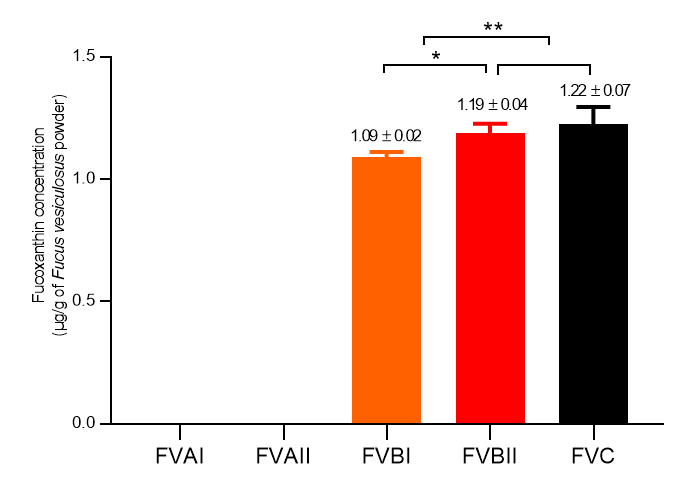


**A.**

**B.**

Fucoxanthin

*m/z* [M+Na]^+^

Fucoxanthin (**40**)

(*m/z* [M+Na]^+^ 681.413; C_42_H_58_O_6_Na; Δ ppm 0)


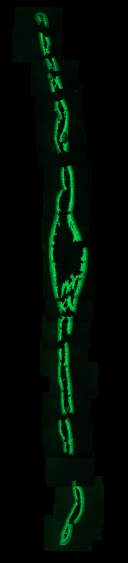


**C.**

| ***After surface extraction by dipping method***  **Stereo-microscope section?** | ***After surface extraction by C18 method*** | ***Untreated (whole Fucus vesiculosus)*** |
| --- | --- | --- |
| ***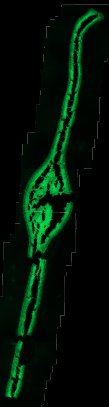***  **DESI-IMS** | 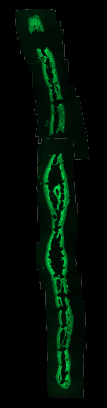 | **High** |
| *m/z* [M+Na]^+^ 681.413  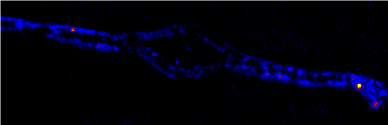  **DESI-IMS closer analysis**  1.42mm | 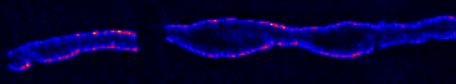  1.66 mm  *m/z* [M+Na]^+^ 681.413  *m/z* [M+Na]^+^ 681.413 | 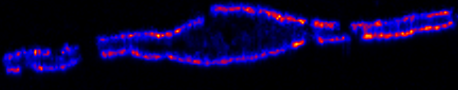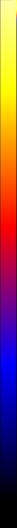  1.34mm |
| 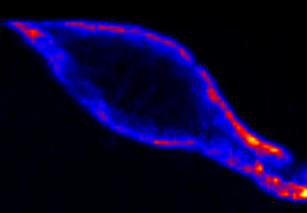  500 µm | **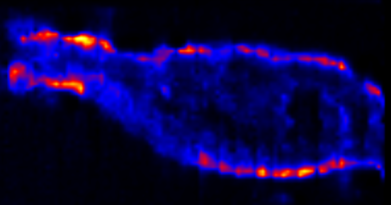**  487 µm | **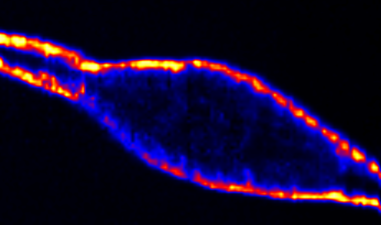**  492 µm  **Low** |

**Figure S9. A.** Mass chromatograms of *F. vesiculosus* extracts in positive mode by UPLC-MS/MS (annotation of the fucoxanthin peak, t_R_ 10.96 min) and fucoxanthin cluster generated by GNPS. **B.** Quantification of fucoxanthin in different extracts (mg/g of *F. vesiculosus* powder). Data are represented as mean and standard deviation of four technical replicates (**: 0.01 <*p*< 0.001; ****: *p*< 0.0001; ns: no significant difference). **C.** Stereomicroscopic images of different algal cross-sections, and spatial distribution of fucoxanthin (*m/z* [M+Na]^+^ 681.413) by DESI-IMS (positive mode) on different cross sections (**Blue**: Surface solvent extract. **Green**: Surface C18 extract. **Orange**: After surface extraction by dipping. **Red**: After surface extraction by C18. **Black**: Untreated (whole) algal extract.

**
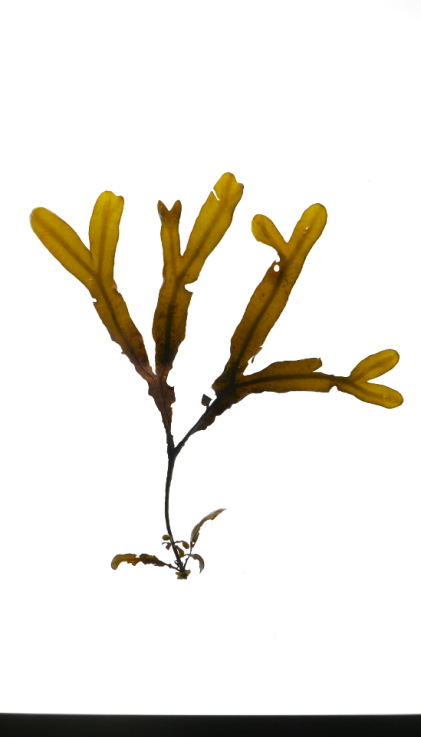

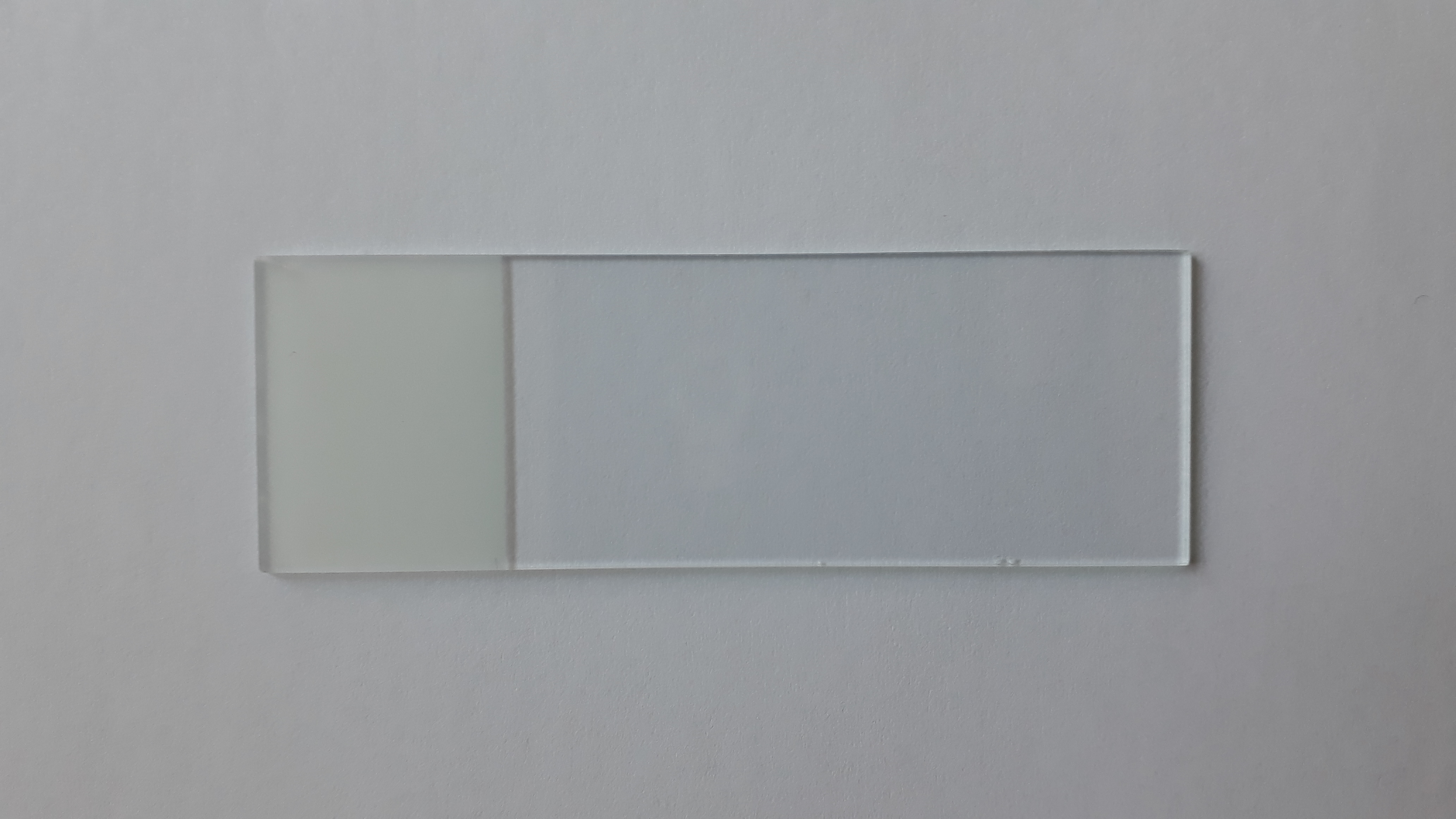

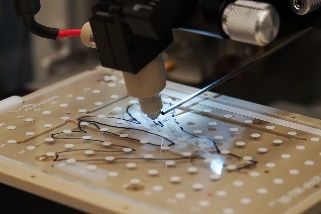

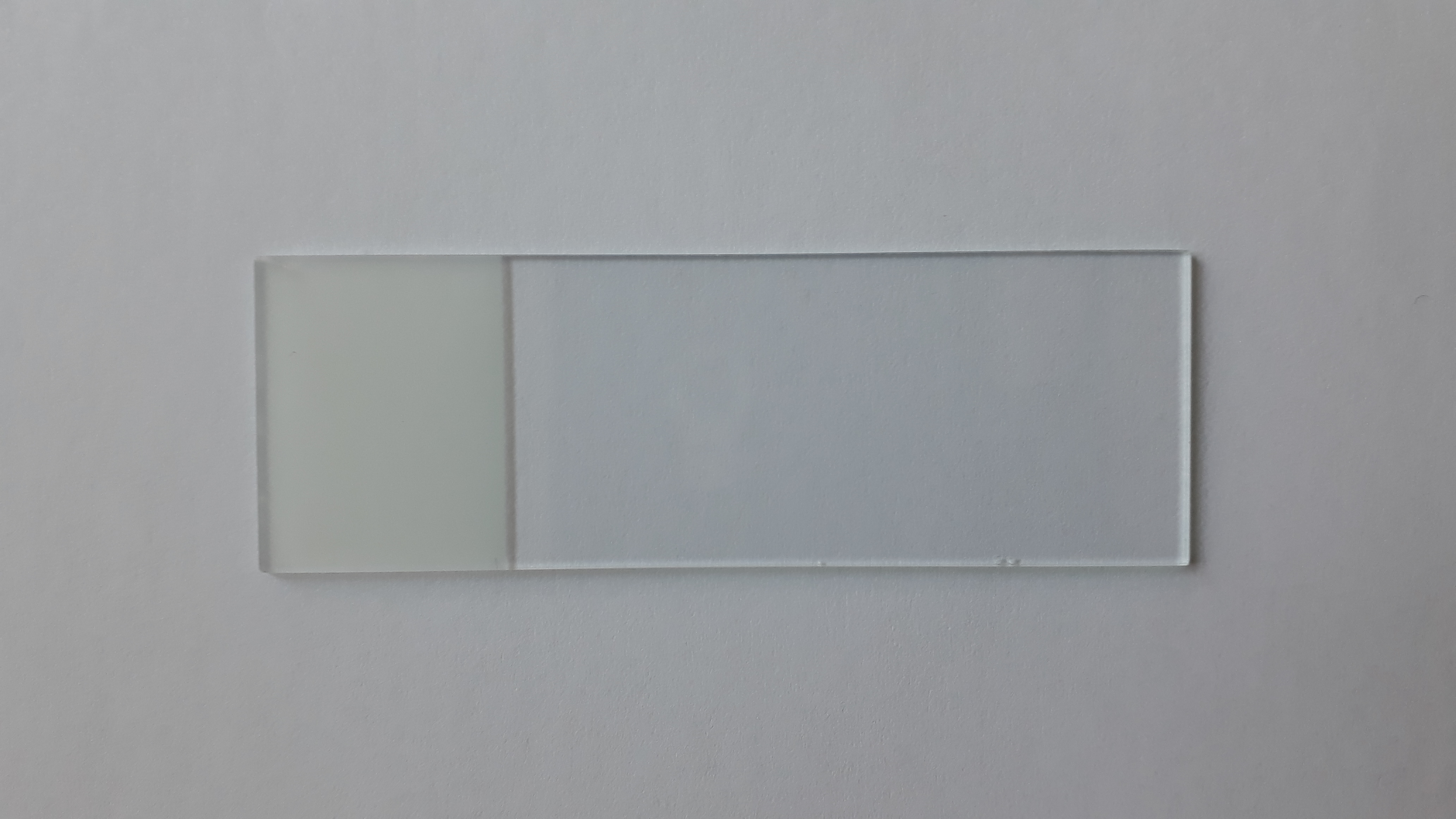
**

**Sample preparation for surface DESI-IMS analyses**

**DESI-IMS analyses**

Surface analyses


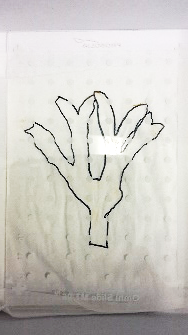

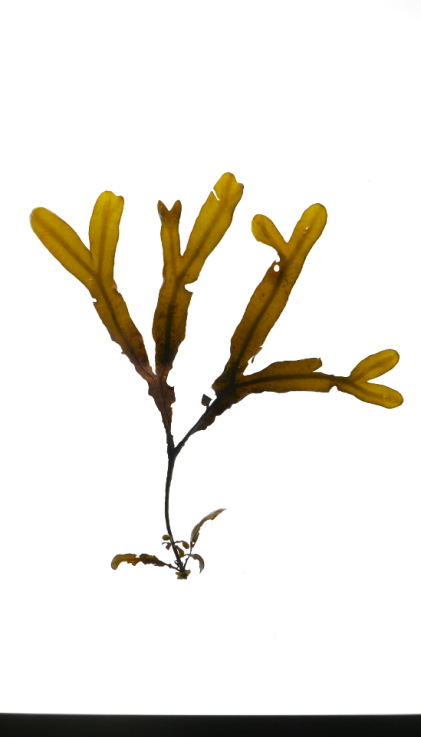


Tight between 2 glass slides during 4 days under 18 Kg

HDImaging

(Waters)

Cross section


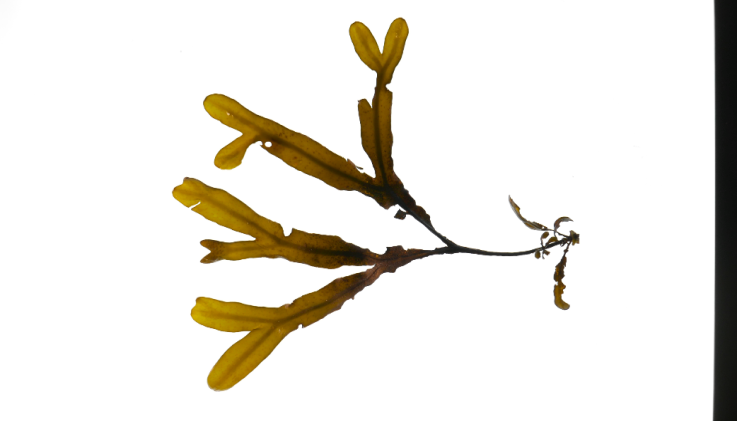

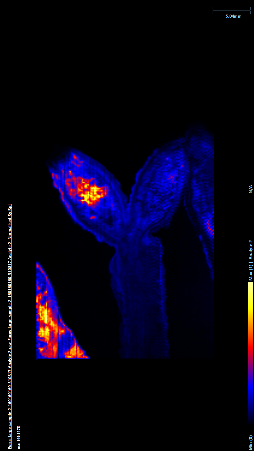

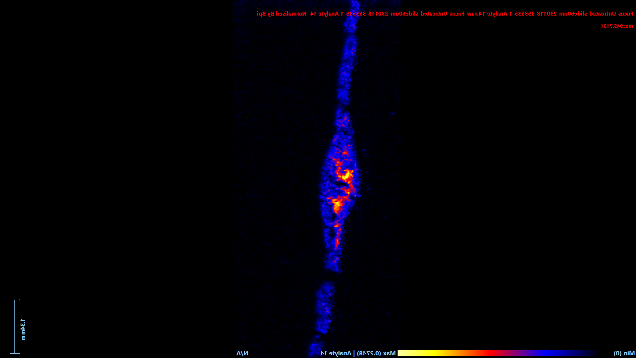


**Samplepreparation for cross section DESI-IMS analyses**


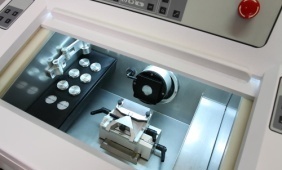


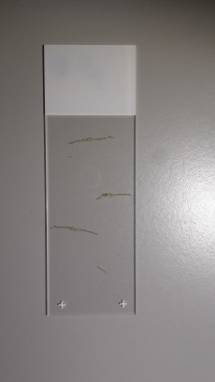


*F. vesiculosus*

Imprints

Cryostat microtome

(Embedding in 2% CMC;

-20°C; thickness 60 µm)

Cross sections

**Figure S10.** Workflow for DESI-IMS analysis. The brown alga was rinsed with artificial seawater for 4 seconds. For preparation of *F. vesiculosus* imprints, several parts (tips and thallus areas) were selected, cut and pressed between 2 microscope slides under weight. The biological material was then removed and imprints were analysed by DESI-IMS in positive mode using MeOH:H_2_O (98:2) as spray solvent. For algal cross section analyses, thallus parts were embedded in 2% CMC (carboxymethyl cellulose) and cut by a cryostat microtome (Leica, -20°C) at a thickness of 60 µm. Cross sections, mounted onto microscope slides, were then also analysed by DESI-IMS under the same conditions as imprints. HDImaging and MassLynx software (version 4.1, Waters) were used for data acquisition and analysis.


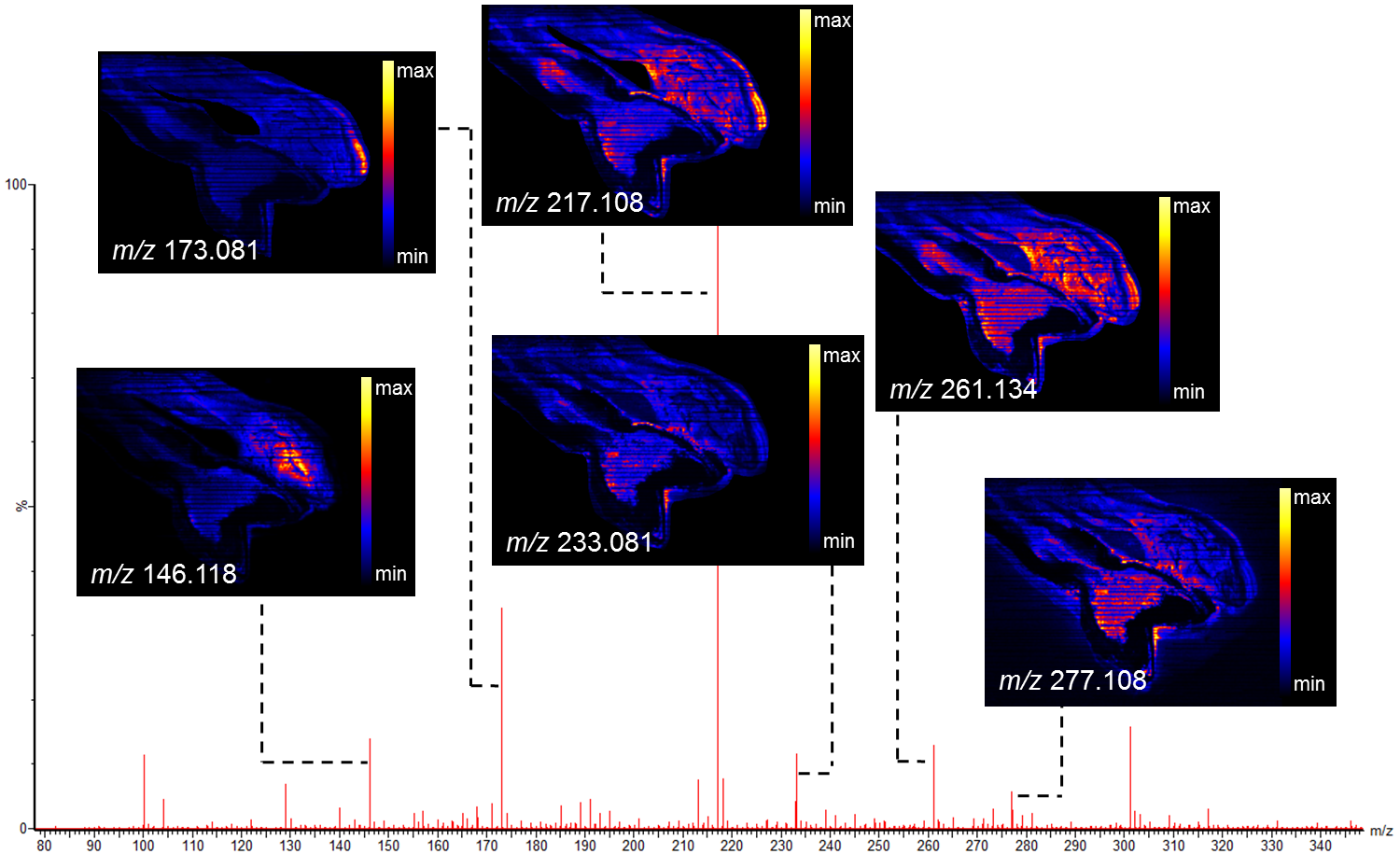


**Figure S11.** DESI-MS images showing the distribution of the major metabolites detected on tip surface imprints of *Fucus vesiculosus* in positive ionisation mode. The spray solvent used is MeOH:H_2_O (98:2).


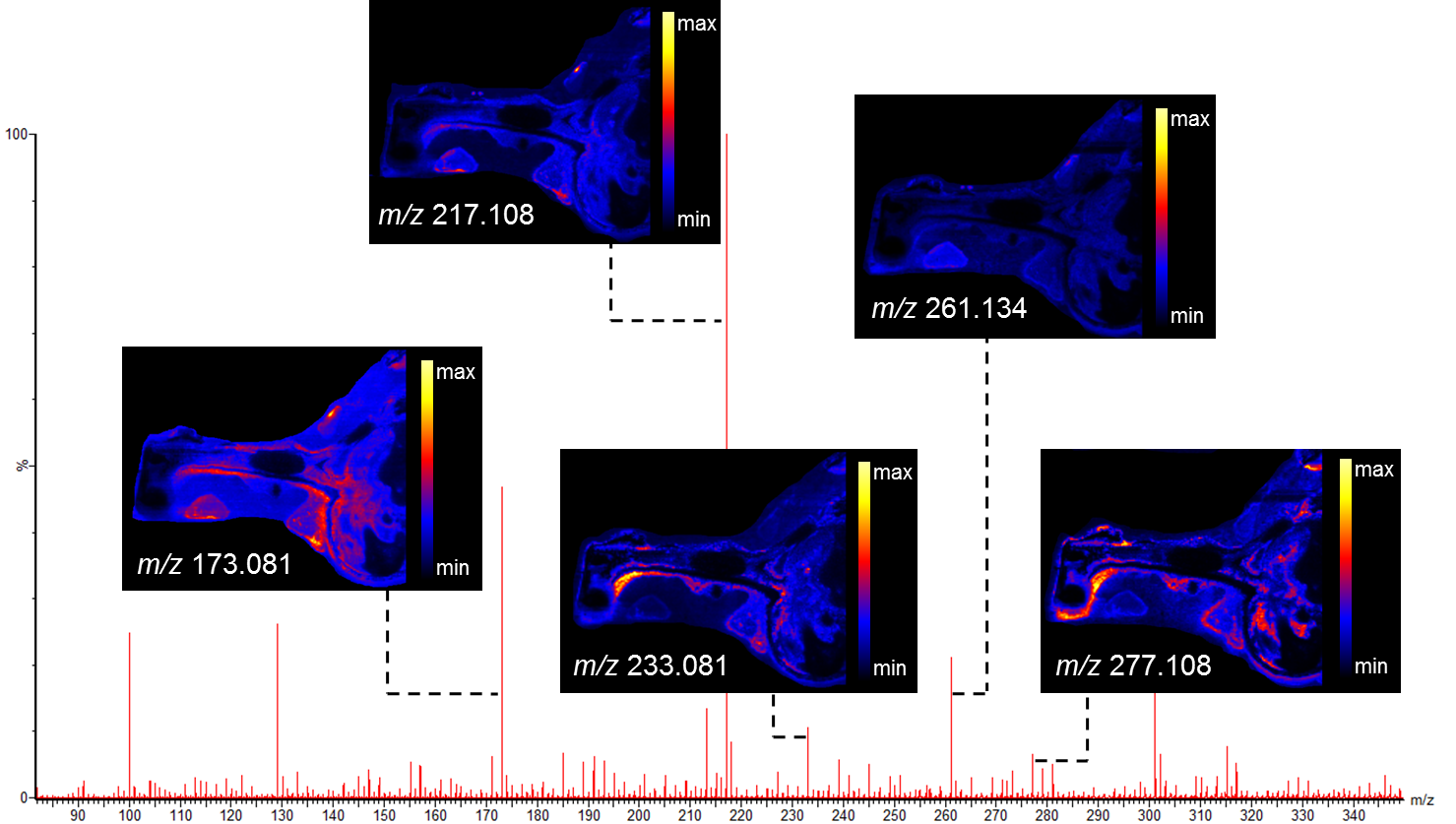


**Figure S12.** DESI-MS images showing the distribution of the major metabolites detected on *Fucus vesiculosus* thallus surface imprints in positive ionisation mode. The spray solvent used is MeOH:H_2_O (98:2).

**References**

1. Sumner, L. W. *et al.* Proposed minimum reporting standards for chemical analysis. Chemical Analysis Working Group (CAWG) Metabolomics Standards Inititative (MSI). *Metabolomics* **3,** 211–221 (2007).

2. Mateus, H., Regenstein, J. M. & Baker, R. C. Studies to improve extraction of mannitol and alginic acid from *Macrocystis pyrifera*, a marine brown alga. *Econ. Bot.* **31,** 24–27 (1977).

3. Iwamoto, K. & Shiraiwa, Y. Salt-regulated mannitol metabolism in algae. *Mar. Biotechnol.* **7,** 407–415 (2005).

4. Ko, R. *et al.* Anti-melanogenesis constituents from the seaweed *Dictyota coriacea*. *Nat. Prod. Commun.* **8,** 427–428 (2013).

5. Heffernan, N., Brunton, N. P., FitzGerald, R. J. & Smyth, T. J. Profiling of the molecular weight and structural isomer abundance of macroalgae-derived phlorotannins. *Mar. Drugs* **13,** 509–528 (2015).

6. Barbosa, M., Valentão, P. & Andrade, P. B. Bioactive compounds from macroalgae in the new millennium: Implications for neurodegenerative diseases. *Mar. Drugs* **12,** 4934–4972 (2014).

7. Singh, I. P. & Sidana, J. Phlorotannins. *In* Dominguez, H. (Ed.): Functional ingredients from algae for foods and nutraceuticals. Woodhead Publishing Series in Food Science, Technology and Nutrition **256**,181-204 (2013)

8. Percot, A. *et al.* Loliolide in marine algae. *Nat. Prod. Res.* **23,** 460–465 (2009).

9. Grabarczyk, M., Wińska, K., Mączka, W., Potaniec, B. & Anioł, M. Loliolide – the most ubiquitous lactone. *Folia Biol. Oecologica* **11,** 1–8 (2015).

10. Okutsu, N., Morohoshi, T., Xie, X., Kato, N. & Ikeda, T. Characterization of *N*-acylhomoserine lactones produced by bacteria isolated from industrial cooling water systems. *Sensors (Switzerland)* **16,** 44 (2016).

11. Smith, R. S. & Iglewski, B. H. *P. aeruginosa* quorum-sensing systems and virulence. *Curr. Opin. Microbiol.* **6,** 56–60 (2003).

12. Glucksam-Galnoy, Y. *et al.* The bacterial quorum-sensing signal molecule *N*-3-oxo-dodecanoyl-L-homoserine lactone reciprocally modulates pro- and anti-inflammatory cytokines in activated macrophages. *J. Immunol.* **191,** 337–344 (2013).

13. Jin, G. *et al.* Two G-protein-coupled-receptor candidates, Cand2 and Cand7, are involved in *Arabidopsis* root growth mediated by the bacterial quorum-sensing signals *N*-acyl-homoserine lactones. *Biochem. Biophys. Res. Commun.* **417,** 991–995 (2012).

14. Hughes, D. T. & Sperandio, V. Inter-kingdom signalling: communication between bacteria and their hosts. *Nature* **6,** 111–120 (2008).

15. Ang, Y. W. *et al.* Quorum sensing signaling molecules involved in the production of violacein by *Pseudoalteromonas*. *Biosci. Biotechnol. Biochem.* **72,** 1958–1961 (2008).

16. Choi, S. Y., Yoon, K., Lee, J. Il & Mitchell, R. J. Violacein: properties and production of a versatile bacterial pigment. *Biomed Res. Int.* ***2015***, 465056 (2015).

17. Hornemam, U., Hurley, L. H., Speedie, M. K. & Floss, H. G. The biosynthesis of indolmycin. *J. Am. Chem. Soc.* **93,** 3028–3035 (1971).

18. Hurdle, J. G., O’Neill, A. J. & Chopra, I. Anti-staphylococcal activity of indolmycin, a potential topical agent for control of staphylococcal infections. *J. Antimicrob. Chemother.* **54,** 549–552 (2004).

19. Zhao, Q. *et al.* Characterization of the Azinomycin B biosynthetic gene cluster revealing a different Iterative type I polyketide synthase for naphthoate biosynthesis. *Chem. Biol.* **15,** 693–705 (2008).

20. Ishizeki, S. et al. Azinomycins A and B, new antitumor antibiotics. *J. Antibiot. (Tokyo)* **40**, 60–65 (1987).

21. Nagaoka, K. *et al.* Azinomycins A and B, new antitumor antibiotics. I. Producing organism, fermentation, isolation, and characterization. *J. Antibiot. (Tokyo)* **39,** 1527–1532 (1986).

22. Inostroza, A. *et al.* Antibiotic activity of emerimicin IV isolated from *Emericellopsis minima* from Talcahuano bay, Chile. *Nat. Prod. Res.* **32,** 1361–1364 (2018).

23. Argoudelis, A. . & Johnson, L. E. Emerimicins II, III and IV, antibiotics produced by *Emericellopsis microspora* in media supplemented with *trans*-4-*n*-propyl-*L*-proline. *J. Antibiot. (Tokyo).* **27,** 274–282 (1974).

24. Eichbenberger, W., Araki, S. & Müller, D. G. Betaine lipids and phospholipids in brown algae. *Phytochemistry* **34,** 1323–1333 (1993).

25. Ding, G. *et al.* Pestalazines and pestalamides, bioactive metabolites from the plant pathogenic fungus *Pestalotiopsis theae*. *J. Nat. Prod.* **71,** 1861–1865 (2008).

26. Giroud, C., Gerber, A. & Eichenberger, W. Lipids of *Chlamydomonas reinhardtii.* Analysis of molecular species and intracellular site(s) of biosynthesis. *Plant Cell Physiol.* **29,** 587–595 (1988).

27. Murakami, H., Nobusawa, T., Hori, K., Shimojima, M. & Ohta, H. Betaine lipid is crucial for adapting to low temperature and phosphate deficiency in *Nannochloropsis*. *Plant Physiol.* **177**, 181-193 (2018).

28. Vogel, G. & Eichenberger, W. Betaine lipids in lower plants. Biosynthesis of DGTS and DGTA in *Ochromonas danica* (Chrysophyceae) and the possible role of DGTS in lipid metabolism. *Plant Cell Physiol.* **33,** 427–436 (2018).

29. de Guzman, F., Bruss, D., Rippentrop, J., Gloer, K. & Gloer, J. Ochrindoles A-D: new bis-indolyl benzenoids from the sclerotia of *Aspergillus ochraceus* NRRL 3519. *J. Nat. Prod.* **57,** 634–639 (1994).

30. Lee, S. K. *et al.* Neopikromycin and novapikromycin from the pikromycin biosynthetic pathway of *Streptomyces venezuelae*. *J. Nat. Prod.* **69,** 847–849 (2006).

31. Cui, Z., Li, Y. S., Liu, H. B., Yuan, D. & Lu, B. R. Sulfoglycolipid from the marine brown alga *Sargassum hemiphylum*. *J. Asian Nat. Prod. Res.* **3,** 117–122 (2001).

32. Johdo, O., Ishikura, T. & Yoshimoto, A. Anthracycline metabolites from *Streptomyces violaceus* A262. I. Isolation of antibiotic-blocked mutants from *Streptomyces violaceu*s A262. *J. Antibiot. (Tokyo).* **44,** 1110–1120 (1991).

33. Johdo, O., Watanabe, Y., Ishikura, T. & Yoshimoto, A. Anthracycline metabolites from *Streptomyces violaceus* A262. III. New anthracycline obelmycins produced by a variant strain SE2-2385. *J. Antibiot. (Tokyo).* **44,** 1030–1140 (1991).

34. Peng, J., Yuan, J. P., Wu, C. F. & Wang, J. H. Fucoxanthin, a marine carotenoid present in brown seaweeds and diatoms: metabolism and bioactivities relevant to human health. *Mar. Drugs* **9,** 1806–1828 (2011).

35. Ratnayake, R., Fremlin, L. J., Lacey, E., Gill, J. H. & Capon, R. J. Acremolides A-D, lipodepsipeptides from an Australian marine-derived fungus, *Acremonium* sp. *J. Nat. Prod.* **71,** 403–408 (2008).

36. Wang, X., Gong, X., Li, P., Lai, D. & Zhou, L. Structural diversity and biological activities of cyclic depsipeptides from fungi. *Molecules* **23,** 169 (2018).

37. Norbert, E., Jenny, T. A., Mooser, V. & Gossauer, A. Chlorophyll catabolism in *Chlorella prorothecoides*. Isolation and structure elucidation of a red bilin derivative. *Fed. Eur. Biochem. Soc.* **293,** 131–133 (1991).

38. Bergy, M. E. & Eble, T. E. The filipin complex. *Biochemistry* **7,** 653–659 (1968).

39. Payero, T. D. *et al.* Functional analysis of filipin tailoring genes from *Streptomyces filipinensis* reveals alternative routes in filipin III biosynthesis and yields bioactive derivatives. *Microb. Cell Fact.* **14,** 1–14 (2015).

40. Konishi, I., Hosokawa, M., Sashima, T., Kobayashi, H. & Miyashita, K. Halocynthiaxanthin and fucoxanthinol isolated from *Halocynthia roretzi* induce apoptosis in human leukemia, breast and colon cancer cells. *Comp. Biochem. Physiol. - C Toxicol. Pharmacol.* **142,** 53–59 (2006).

41. Torregrosa-Crespo, J. *et al.* Exploring the valuable carotenoids for the large-scale production by marine microorganisms. *Mar. Drugs* **16,** 203 (2018).

42. Lee, S. H. *et al.* Bahamaolide A from the marine-derived *Streptomyces* sp. CNQ343 inhibits isocitrate lyase in *Candida albicans*. *Bioorganic Med. Chem. Lett.* **24,** 4291–4293 (2014).

43. Kim, D. G. *et al.* Bahamaolides A and B, antifungal polyene polyol macrolides from the marine actinomycete *Streptomyces* sp. *J. Nat. Prod.* **75,** 959–967 (2012).

44. Yu, Z., Lang, G., Kajahn, I., Schmaljohann, R. & Imhoff, J. F. Scopularides A and B, cyclodepsipeptides from a marine sponge-derived fungus, *Scopulariopsis brevicaulis*. *J. Nat. Prod.* **71,** 1052–1054 (2008).

45. Burke, R.C., Swartz J.H., Chapman, S.S. & Huang, W.Y. Mycoticin, a new antifungal antibiotic. *J. Invest. Dermatol.* **23**,163–169 (1954).

46. Dupont, J. *et al.* Farnesylacetone, a sesquiterpenic hormone of crustacea, inhibits electron transport in isolated rat liver mitochondria. *Biol. Cell* **67,** 141–146 (1989).

47. Shizuri, Y., Matsukawa, S., Ojika, M. & Yamada, K. Two new farnesylacetone derivatives from the brown alga *Sargassum micracanthum*. *Phytochemistry* **21,** 1808–1809 (1982).

48. Hirschman, R., Snaddy, S. C., Hiskey, C. F., Wendler, N. L. & Hayashi, S. Two derivatives of farnesylacetone from the brown alga *Cystophora moniliformis*. *Roche Res. Inst. Mar. Pharmacol.* **15,** 156–157 (1978).

49. Bertin, M. J., Zimba, P. V., Beauchesne, K. R., Huncik, K. M. & Moeller, P. D. R. Identification of toxic fatty acid amides isolated from the harmful alga *Prymnesium parvum carter*. *Harmful Algae* **20,** 111–116 (2012).

50. Trifonov, L. S. *et al.* Isolation and structure elucidation of three metabolites from *Verticillium intertextum*: sorbicillin, dihydrosorbicillin and bisvertinoquinol. *Tetrahedron* **39,** 4243–4256 (1983).

51. Maskey, R. P., Grün-Wollny, I. & Laatsch, H. Sorbicillin analogues and related dimeric compounds from *Penicillium notatum*. *J. Nat. Prod.* **68,** 865–870 (2005).

52. Hsu, C., Chao, P., Hu, S. & Yang, C. The antioxidant and free radical scavenging activities of chlorophylls and pheophytins. *Food Nutr. Sci.* **4,** 1–8 (2013).

53. Yu, C.-C. *et al.* Chemical composition and bioactivities of the marine alga *Isochrysis galbana* from Taiwan. *Nat. Prod. Commun.* **5,** 1941–1944 (2010).

54. Cheng, H. *et al.* Cytotoxic pheophorbide-related compounds from *Clerodendrum calamitosum*. *J. Nat. Prod.* **64,** 915–919 (2001).

55. Tang, P. M. K. *et al.* Pheophorbide a, an active compound isolated from *Scutellaria barbata*, possesses photodynamic activities by inducing apoptosis in human hepatocellular carcinoma. *Cancer Biol. Ther.* **5,** 1111–1116 (2006).

56. Hong, C. O. *et al.* Pheophorbide a from *Capsosiphon fulvescens* inhibits advanced glycation end products mediated endothelial dysfunction. *Planta Med.* **82,** 46–57 (2015).

57. Islam, N. *et al.* Anti-inflammatory activity of edible brown alga *Saccharina japonica* and its constituents pheophorbide a and pheophytin a in LPS-stimulated RAW 264.7 macrophage cells. *Food Chem. Toxicol.* **55,** 541–548 (2013).

58. Betina, V., Micekova, D. & Nemec, P. Antimicrobial properties of cytochalasins and their alteration of fungal morphology. *Microbiology* **71,** 343–349 (1972).

59. Glombitza, K., Keusgen, M. & Hauperich, S. Fucophloretols from the brown algae *Sargassum spinuligerum* and *Cystophora torulosa*. *Phytochemistry* **46,** 1417–1422 (1997).

60. Atta-Ur-Rahman, S. Z., Choudhary, M. I., Abbas, S. A. & Shameel, M. Stockerine a novel linear metabolite from *Stockeyia indica*. *Fitoterapia* **62,** 77–80 (1991).

61. Ina, A. & Kamei, Y. Vitamin B12, a chlorophyll-related analog to pheophytin a from marine brown algae, promotes neurite outgrowth and stimulates differentiation in PC12 cells. *Cytotechnology* **52,** 181–187 (2006).

62. Okai, Y. & Higashi-Okai, K. Potent anti-inflammatory activity of pheophytin A derived from edible green alga, *Enteromorpha prolifera* (Sujiao-Nori). *Int. J. Immunopharmacol.* **19,** 355–358 (1997).
